# Supplementary material for: Individualized prevention of proton pump inhibitor related adverse events by risk stratification
Source: Nat Commun. 2024 Apr 27;15:3591. doi: 10.1038/s41467-024-48007-8 (PMC11055952; doi:10.1038/s41467-024-48007-8)
Supplement: Supplementary file 1 — Supplementary Information [file 41467_2024_48007_MOESM1_ESM.pdf]

Individualized Prevention of Proton Pump Inhibitor related Adverse Events by Risk Stratification

Bin Xia, PhD [1,2*]; Qiangsheng He, MM [1,2*]; Fang Gao Smith, MD, PhD [3,4*]; Georgios Gkoutos, PhD [5,6,7]; Krish Nirantharakumar, PhD [6,7]; Zi Chong Kuo, MD [8]; Danni Wang, MS [1,2]; Qi Feng, PhD [9]; Eddie C. Cheung, MD [8,10]; Lunzhi Dai, PhD [11], Junjie Huang, PhD [12]; Yuanyuan Yu, MD, PhD [13]; Wenbo Meng, MD, PhD [14 #]; Xiwen Qin, PhD [15,16,17 #]; Jinqiu Yuan, PhD [1,2,8 #]

Supplemental Materials

Content

Supplementary Table 1. Baseline characteristics of the included participants in the UK Biobank 5
Supplementary Table 2. Baseline characteristics of the included participants in the NHS cohort 6
Supplementary Table 3. Baseline characteristics of the included participants in the NHS II cohort 7
Supplementary Table 4. Baseline characteristics of the included participants in the HPFS cohort 8
Supplementary Table 5. Baseline characteristics of the included participants in the CDARS database 9
Supplementary Table 6. Association between regular PPI use and risk of ischemic heart disease 10
Supplementary Table 7. Association between regular PPI use and risk of stroke 11
Supplementary Table 8. Association between regular PPI use and risk of chronic obstructive pulmonary disease 12
Supplementary Table 9. Association between regular PPI use and risk of alzheimer's disease and other dementias 13
Supplementary Table 10. Association between regular PPI use and risk of diabetes mellitus 14
Supplementary Table 11. Association between regular PPI use and risk of lower respiratory infections 15
Supplementary Table 12. Association between regular PPI use and risk of tracheal, bronchus, and lung cancer 16
Supplementary Table 13. Association between regular PPI use and risk of falls 17
Supplementary Table 14. Association between regular PPI use and risk of chronic kidney disease 18
Supplementary Table 15. Association between regular PPI use and risk of age-related hearing loss 19
Supplementary Table 16. Association between regular PPI use and risk of

hypertensive heart disease 20
Supplementary Table 17. Association between regular PPI use and risk of diarrheal diseases 21
Supplementary Table 18. Association between regular PPI use and risk of low back pain 22
Supplementary Table 19. Association between regular PPI use and risk of colon and rectum cancer 23
Supplementary Table 20. Association between regular PPI use and risk of blindness and vision loss 24
Supplementary Table 21. Association between regular PPI use and risk of atrial fibrillation and flutter 25
Supplementary Table 22. Association between regular PPI use and risk of stomach cancer 26
Supplementary Table 23. Association between regular PPI use and risk of prostate cancer 27
Supplementary Table 24. Association between regular PPI use and risk of cirrhosis and other chronic liver diseases 28
Supplementary Table 25. Association between regular PPI use and risk of parkinson's disease 29
Supplementary Table 26. Association between regular PPI use and risk of osteoarthritis 30
Supplementary Table 27. Association between regular PPI use and risk of tuberculosis 31
Supplementary Table 28. Association between regular PPI use and risk of asthma 32
Supplementary Table 29. Association between regular PPI use and risk of road injuries 33
Supplementary Table 30. Association between regular PPI use and risk of pancreatic cancer 34
Supplementary Table 31. Association between regular PPI use and risk of depressive disorders 35
Supplementary Table 32. Association between regular PPI use and risk of breast cancer 36
Supplementary Table 33. Association between regular PPI use and risk of esophageal cancer 37
Supplementary Table 34. Association between regular PPI use and risk of liver cancer 38
Supplementary Table 35. Association between regular PPI use and risk of cardiomyopathy and myocarditis 39
Supplementary Table 36. Performance of the prediction models for any of 15 unintended outcomes in derived population and validation population 40
Supplementary Figure 1. Sensitive analyses for the associations between regular PPI use and risk of the major high-burden diseases 41
Supplementary Figure 2. Value of the joint minimum strength of association on the risk ratio scale that an unmeasured confounder must have with the exposure and

the outcome to fully explain away an observed HR between PPI and the outcome. 42
Supplementary Figure 3. Pooled hazards ratios by individual class of PPIs in UK Biobank and CDARS. 43
Supplementary Figure 4. Pooled associations between regular PPI use with risk of the major diseases stratified by sex 44
Supplementary Figure 5. Pooled associations between regular PPI use with risk of the major diseases stratified by age 45
Supplementary Figure 6. Pooled associations between regular PPI use with risk of the major diseases stratified by BMI 46
Supplementary Figure 7. Nomogram for predicting the 1-, 5- and 10-year probability of any of 15 unintended outcomes. 47
Supplementary Figure 8. Predicted and observed 10-year risk of 15 major diseases by 10th of predicted risk in derived population and validation population 48
Supplementary Figure 9. Sensitivity analysis: PPI-related absolute risk for major high-burden diseases according to the distribution of the baseline predicted risk after excluding osteoarthritis. 49
Supplementary Figure 10. Study design of the present study. 50
Supplementary Figure 11. Framework for ischemic heart disease in the present study. 51
Supplementary Figure 12. Framework for stroke in the present study. 52
Supplementary Figure 13. Framework for chronic obstructive pulmonary disease in the present study. 53
Supplementary Figure 14. Framework for alzheimer's disease and other dementias in the present study. 54
Supplementary Figure 15. Framework for diabetes mellitus in the present study. 55
Supplementary Figure 16. Framework for lower respiratory infections in the present study. 56
Supplementary Figure 17. Framework for tracheal, bronchus, and lung cancer in the present study. 57
Supplementary Figure 18. Framework for falls in the present study. 58
Supplementary Figure 19. Framework for chronic kidney disease in the present study. 59
Supplementary Figure 20. Framework for age-related hearing loss in the present study. 60
Supplementary Figure 21. Framework for hypertensive heart disease in the present study. 61
Supplementary Figure 22. Framework for diarrheal diseases in the present study. 62
Supplementary Figure 23. Framework for low back pain in the present study. 63
Supplementary Figure 24. Framework for colon and rectum cancer (CRC) in the present study. 64
Supplementary Figure 25. Framework for blindness and vision loss in the present study. 65
Supplementary Figure 26. Framework for atrial fibrillation and flutter in the present study. 66

Supplementary Figure 27. Framework for stomach cancer in the present study. 67
Supplementary Figure 28. Framework for prostate cancer in the present study. 68
Supplementary Figure 29. Framework for cirrhosis and other chronic liver diseases in the present study. 69
Supplementary Figure 30. Framework for Parkinson's disease in the present study. 70
Supplementary Figure 31. Framework for osteoarthritis in the present study. 71
Supplementary Figure 32. Framework for tuberculosis in the present study. 72
Supplementary Figure 33. Framework for asthma in the present study. 73
Supplementary Figure 34. Framework for road injuries in the present study. 74
Supplementary Figure 35. Framework for pancreatic cancer in the present study. 75
Supplementary Figure 36. Framework for depressive disorders in the present study. 76
Supplementary Figure 37. Framework for breast cancer in the present study. 77
Supplementary Figure 38. Framework for esophageal cancer in the present study. 78
Supplementary Figure 39. Framework for liver cancer in the present study. 79
Supplementary Figure 40. Framework for cardiomyopathy and myocarditis in the present study. 80
STROBE Statement—Checklist of items that should be included in reports of *cohort studies* 81

Supplementary Table 1. Baseline characteristics of the included participants in the UK Biobank

Characteristics	Non-regular PPI user (n=449 868)	Regular PPI user (n=51 241)	Overall (N=501 109)
Mean (SD) age (years)	56.7 (8.1)	60.1 (7.2)	57.0 (8.1)
Female, No. (%)	244 492 (54.3)	28 140 (54.9)	272 632 (54.4)
White, No. (%)	425 442 (94.6)	48 761 (95.2)	474 203 (94.6)
Postmenopausal, No. (%)	170 093 (37.8)	24 016 (46.9)	194 109 (38.7)
Mean (SD) BMI, kg/m^2	27.2 (4.72)	29.2 (5.17)	27.4 (4.80)
Never smoker, No. (%)	251 778 (56.0)	23 970 (46.8)	275 748 (55.0)
No-alcohol drinkers, No. (%)	34 124 (7.6)	6386 (12.5)	40 510 (8.1)
Median (IQR) physical activity, MET hours/week	29.8 (45.9)	25.5 (45.6)	29.5 (46.0)
> 5 portions of fruit and vegetable per day, No. (%)	169 930 (37.8)	19 416 (37.9)	189 346 (37.8)
Prevalent comorbidities, No. (%)			
GERD	13 730 (3.1)	22 458 (43.8)	36 188 (7.2)
Gastric or duodenal ulcer	6625 (1.5)	5200 (10.1)	11 825 (2.4)
Upper gastrointestinal tract bleeding	2468 (0.5)	1627 (3.2)	4095 (0.8)
Cancer	24 374 (5.4)	4316 (8.4)	28 690 (5.7)
Hypertension	257 606 (57.3)	36 723 (71.7)	294 329 (58.7)
Hypercholesterolaemia	74 650 (16.6)	18 946 (37.0)	93 596 (18.7)
Diabetes	23 907 (5.3)	6060 (11.8)	29 967 (6.0)
Current medication use, No. (%)			
Multivitamin	66 388 (14.8)	9119 (17.8)	75 507 (15.1)
Hormone	16 986 (3.8)	2727 (5.3)	19 713 (3.9)
Aspirin	59 259 (13.2)	12 669 (24.7)	71 928 (14.4)
NSAIDs	134 074 (29.8)	21 574 (42.1)	15 5648 (31.1)
Statin	64 784 (14.4)	16 944 (33.1)	81 728 (16.3)
ACEIs	40 294 (9.0)	9159 (17.9)	49 453 (9.9)
Beta-blockers	25 191 (5.6)	7208 (14.1)	32 399 (6.5)
Calcium channel blockers	25 242 (5.6)	6083 (11.9)	31 325 (6.3)
Thiazide diuretics	25 751 (5.7)	5354 (10.4)	31 105 (6.2)
Good health rating, No. (%)	344 916(76.7)	24 910 (48.6)	369 826(73.8)
Longstanding illness, No. (%)	128 772 (28.6)	30 735 (60.0)	159 507 (31.8)

Abbreviation: SD, standard deviation; IQR, interquartile range; MET, Metabolic equivalent of task; GERD, gastroesophageal reflux disease; NSAIDs, Non-steroidal anti-inflammatory drugs; ACEIs, Angiotensin-converting enzyme inhibitors.

Supplementary Table 2. Baseline characteristics of the included participants in the NHS cohort

Characteristics	Non-regular PPI user (N=86 182)	Regular PPI user (N=5 526)	Overall (N=91 708)
Mean (SD) age (years)	68.4 (7.1)	68.9 (7.1)	68.4 (7.1)
Female, No. (%)	86 182 (100.0)	5526 (100.0)	91 708 (100.0)
White, No. (%)	83 743 (97.2)	5415 (98)	89 158 (97.2)
Postmenopausal, No. (%)	85 798 (99.6)	5509 (99.7)	91 307 (99.6)
Mean (SD) BMI, kg/m^2	26.8 (5.4)	28.3 (5.7)	26.9 (5.4)
Never smoker, No. (%)	39 714 (46.1)	2412 (43.7)	42 126 (45.9)
No-alcohol drinkers, No. (%)	30 799 (35.7)	2497 (45.2)	33 296 (36.3)
Median (IQR) physical activity, MET hours/week	13.6 (31.4)	7.7 (16.8)	12.9 (30.3)
> 5 portions of fruit and vegetable per day, No. (%)	18 583 (21.6)	1160 (21)	19 743 (21.5)
Mean (SD) AHEI	50.8 (10.4)	50 (10.4)	50.8 (10.4)
Prevalent comorbidities, No. (%)			
GERD	23 016 (26.7)	4164 (75.4)	27 180 (29.6)
Gastric or duodenal ulcer	2138 (2.5)	681 (12.3)	2819 (3.1)
Upper gastrointestinal tract bleeding	1471 (1.7)	247 (4.5)	1718 (1.9)
Cancer	12 696 (14.7)	979 (17.7)	13 675 (14.9)
Hypertension	41 524 (48.2)	3520 (63.7)	45 044 (49.1)
Hypercholesterolaemia	51 708 (60)	4073 (73.7)	55 781 (60.8)
Diabetes	7600 (8.8)	657 (11.9)	8257 (9)
Current medication use, No. (%)			
Multivitamin	49 060 (56.9)	3333 (60.3)	52 393 (57.1)
Hormone	26 869 (31.2)	2026 (36.7)	28 895 (31.5)
Aspirin	15 078 (17.5)	948 (17.2)	16 026 (17.5)
NSAIDs	55 378 (64.3)	4118 (74.5)	59 496 (64.9)
Statin	17 321 (20.1)	1767 (32.0)	19 088 (20.8)
ACEIs	9806 (11.4)	893 (16.2)	10 699 (11.7)
Beta-blockers	13 130 (15.2)	1305 (23.6)	14 435 (15.7)
Calcium channel blockers	7174 (8.3)	734 (13.3)	7908 (8.6)
Thiazide diuretics	11 104 (12.9)	1075 (19.5)	12 179 (13.3)
Antidepressants	7966 (9.2)	1109 (20.1)	9075 (9.9)
Antibiotics	70 809 (82.2)	4783 (86.6)	75 592 (82.4)

Abbreviation: SD, standard deviation; IQR, interquartile range; MET, Metabolic equivalent of task; AHEI, Alternate Healthy Eating Index; GERD, gastroesophageal reflux disease; NSAIDs, Non-steroidal anti-inflammatory drugs; ACEIs, Angiotensin-converting enzyme inhibitors.

Supplementary Table 3. Baseline characteristics of the included participants in the NHS II cohort

Characteristics	Non-regular PPI user (N=94 419)	Regular PPI user (N=5222)	Overall (N=99 641)
Mean (SD) age (years)	48.7 (4.6)	49.8 (4.4)	48.8 (4.6)
Female, No. (%)	94 419 (100.0)	5,222 (100.0)	99 641 (100.0)
White, No. (%)	90 653 (96.0)	5068 (97.1)	95 721 (96.1)
Postmenopausal, No. (%)	45 848 (48.6)	3334 (63.9)	49 182 (49.4)
Mean (SD) BMI, kg/m^2	26.8 (6.3)	30.1 (7.5)	27 (6.4)
Never smoker, No. (%)	61 745 (65.4)	3144 (60.2)	64 889 (65.1)
No-alcohol drinkers, No. (%)	23 950 (25.4)	1727 (33.1)	25 677 (25.8)
Median (IQR) physical activity, MET hours/week	17.4 (42.8)	8.2 (18.6)	16.7 (40.5)
> 5 portions of fruit and vegetable per day, No. (%)	16 741 (17.7)	859 (16.5)	17 600 (17.7)
Mean (SD) AHEI score	51.6 (10.2)	50.6 (10.4)	51.5 (10.3)
Prevalent comorbidities, No. (%)			
GERD	23 750 (25.2)	3898 (74.7)	27 648 (27.8)
Gastric or duodenal ulcer	1719 (1.8)	722(13.8)	2441 (2.4)
Upper gastrointestinal tract bleeding	464 (0.5)	131 (2.5)	595 (0.6)
Cancer	2639 (2.8)	232 (4.4)	2871 (2.9)
Hypertension	15 792 (16.7)	1679 (32.2)	17 471 (17.5)
Hypercholesterolaemia	26 946 (28.5)	2463 (47.2)	29 409 (29.5)
Diabetes	2675 (2.8)	365 (7)	3040 (3.1)
Current medication use, No. (%)			
Multivitamin	45 900 (48.6)	3038 (58.2)	48 938 (49.1)
Hormone	15 827 (16.8)	1507 (28.9)	17 334 (17.4)
Aspirin	8421 (8.9)	615 (11.8)	9036 (9.1)
NSAIDs	46 687 (49.5)	3353 (64.2)	50 040 (50.2)
Statin	5691 (6.0)	808 (15.5)	6499 (6.5)
ACEIs	3740 (4.0)	407 (7.8)	4147 (4.2)
Beta-blockers	5140 (5.4)	662 (12.7)	5802 (5.8)
Calcium channel blockers	1773 (1.9)	263 (5.0)	2036 (2.0)
Thiazide diuretics	4659 (4.9)	608 (11.6)	5267 (5.3)
Antidepressants	13 865 (14.7)	1691 (32.4)	15 556 (15.6)
Antibiotics	80 519 (85.3)	4725 (90.5)	85 244 (85.6)

Abbreviation: SD, standard deviation; IQR, interquartile range; MET, Metabolic equivalent of task; AHEI, Alternate Healthy Eating Index; GERD, gastroesophageal reflux disease; NSAIDs, Non-steroidal anti-inflammatory drugs; ACEIs, Angiotensin-converting enzyme inhibitors.

Supplementary Table 4. Baseline characteristics of the included participants in the HPFS cohort

Characteristics	Non-regular PPI user (n=26 383)	Regular PPI user (n=4550)	Overall (N=30 933)
Mean (SD) age (years)	71.2 (8.4)	72.6 (8.4)	71.4 (8.4)
Female, No. (%)	0 (0)	0 (0)	0 (0)
White, No. (%)	24 066 (91.2)	4177 (91.8)	28 243 (91.3)
Mean (SD) BMI, kg/m^2	23.4 (8.9)	23.8 (8.7)	23.5 (8.8)
Never smoker, No. (%)	12 463 (47.2)	2044 (44.9)	14 507 (46.9)
No-alcohol drinkers, No. (%)	4884 (18.5)	943 (20.7)	5827 (18.8)
Median (IQR) physical activity, MET hours/week	39.5 (71.5)	33.3 (60.5)	38.4 (69.9)
> 5 portions of fruit and vegetable per day, No. (%)	5428 (20.6)	970 (21.3)	6398 (20.7)
Mean (SD) AHEI score	56.2 (10)	55.5 (10.2)	56.1 (10)
Prevalent comorbidities, No. (%)			
GERD	5431 (20.6)	3290 (72.3)	8721 (28.2)
Gastric or duodenal ulcer	738 (2.8)	467 (10.3)	1205 (3.9)
Upper gastrointestinal tract bleeding	537 (2)	200 (4.4)	737 (2.4)
Cancer	3499 (13.3)	735 (16.2)	4234 (13.7)
Hypertension	12 934 (49)	2739 (60.2)	15 673 (50.7)
Hypercholesterolaemia	15 643 (59.3)	3102 (68.2)	18 745 (60.6)
Diabetes	2663 (10.1)	492 (10.8)	3155 (10.2)
Current medication use, No. (%)			
Multivitamin	16 154 (61.2)	3254 (71.5)	19 408 (62.7)
Aspirin	13 316 (50.5)	2521 (55.4)	15 837 (51.2)
NSAIDs	16 241 (61.6)	3149 (69.2)	19 390 (62.7)
Statin	9182 (34.8)	2113 (46.4)	11 295 (36.5)
ACEIs	3847 (14.6)	920 (20.2)	4767 (15.4)
Beta-blockers	4057 (15.4)	1021 (22.4)	5078 (16.4)
Calcium channel blockers	2028 (7.7)	530 (11.7)	2558 (8.3)
Thiazide diuretics	2546 (9.7)	622 (13.7)	3168 (10.2)
Antidepressants	366 (1.4)	116 (2.6)	482 (1.6)
Antibiotics	16 557 (62.8)	2838 (62.4)	19 395 (62.7)

Abbreviation: SD, standard deviation; IQR, interquartile range; MET, Metabolic equivalent of task; AHEI, Alternate Healthy Eating Index; GERD, gastroesophageal reflux disease; NSAIDs, Non-steroidal anti-inflammatory drugs; ACEIs, Angiotensin-converting enzyme inhibitors.

Supplementary Table 5. Baseline characteristics of the included participants in the CDARS database

Characteristics	Regular H2RA user (n=633 498)	Regular PPI user (n=722 835)	Overall (n=1 356 333)
Mean (SD) age (years)	67.3 (7.0)	67.8 (7.0)	67.5 (7.0)
Female, No. (%)	190 050 (30%)	274 677 (38%)	464 727 (34.3)
Prevalent comorbidities, No. (%)			
GERD	511 233 (80.7)	615 855 (85.2)	1 127 088 (83.1)
Gastric or duodenal ulcer	489 060 (77.2)	596 339 (82.5)	1 085 399 (80.0)
Upper gastrointestinal tract bleeding	517 568 (81.7)	638 263 (88.3)	1 155 831 (85.2)
Cancer	74 119 (11.7)	149 627 (20.7)	223 746 (16.5)
Hypertension	502 997 (79.4)	578 990 (80.1)	1 081 987 (79.8)
Hypercholesterolaemia	94 391 (14.9)	199 502 (27.6)	293 893 (21.7)
Diabetes	193 850 (30.6)	259 498 (35.9)	453 348 (33.4)
Current medication use, No. (%)			
Aspirin	22 7426 (35.9)	509 599 (70.5)	737 025 (54.3)
NSAIDs	359 827(56.8)	442 375(61.2)	802 202 (59.1)
Statin	131 134(20.7)	217 573 (30.1)	348 707 (25.7)
ACEIs	522 635 (82.5)	617 301 (85.4)	1 139 936 (84.0)
Beta-blockers	501 730 (79.2)	513 936 (71.1)	1 015 666 (74.9)
Calcium channel blockers	499 823 (78.9)	538 119 (74.4)	1 037 942 (76.5)
Thiazide diuretics	461 092 (72.8)	462 230 (63.9)	923 322 (68.1)
Antidepressants	10 945 (1.7)	20 777 (2.9)	31 722 (2.3)
Antibiotics	9011 (1.4)	10 775 (1.5)	19 786 (1.5)

Abbreviation: H2RA, Histamine Type-2 Receptor Antagonists; SD, standard deviation; GERD, gastroesophageal reflux disease; NSAIDs, Non-steroidal anti-inflammatory drugs; ACEIs, Angiotensin-converting enzyme inhibitors.

Supplementary Table 6. Association between regular PPI use and risk of ischemic heart disease

Cohorts	Cases	Person-years	IR in non-PPI users *	IR in PPI users *	M-H, Random HR [95%CI]
UKB	30 104	5 584 474	494.5	1023	1.25 [1.2,1.29] [a]
NHS	3900	893 588	424.6	511.6	1.21 [1.1,1.33] [b]
NHS II	1033	1 185 935	78.9	146.3	1.17 [0.99,1.38] [b]
HPFS	518	200 340	254.5	284.3	1.15 [0.88,1.5] [c]
CDARS	8663	5 762 730	106.4	226.4	1.10 [1.02, 1.18] [d]
Combined	44 218	13 627 067	313.5	365.6	1.19 [1.11,1.27] [e]

Abbreviation: IR, incidence rate; M-H, Mantel-Haenszel; HR, hazard ratio; CI, confidence interval; UKB, UK Biobank; NHS, Nurses' Health Study; HPFS, Health Professionals Follow-Up Study; CDARS, Clinical Data Analysis and Reporting System.

* Per 100 000 person years.

[a] assessed by multivariable-adjusted Cox proportional hazards model stratified by age, sex and UK assessment centres, and additionally adjusted for ethnicity (white or other), BMI (<18.5, 18.5–24.9, 25–30, ≥30), smoking status (never smoked, previous smoker, current smoker), alcohol consumption (never or special occasions only, 1–3 times/mo, 1–4 times/wk, daily or almost daily), physical activity (metabolic equivalent of task <9 h/wk, 9–27 h/wk, ≥27 h/wk, unknown/missing), daily sleep duration (<8, 8, 8-9, ≥9 hours), portions of fruit and vegetable intake (<5 portions/d, ≥5 portions/d), family history of heart disease (yes or no), clinical indication for PPI use (GERD, gastric or duodenal ulcer, gastrointestinal bleeding), medications (multivitamin use, NSAID, aspirin, statin, ACEIs, beta-blockers, calcium-channel blockers, thiazide diuretics, metformin, antibiotic), comorbidities (diabetes, hypertension, hypercholesterolaemia), overall health rating (poor, fair, good, excellent) and longstanding illness (Yes, No).

[b] assessed by multivariable-adjusted Cox proportional hazards model stratified by age and time period (in 2-year intervals) and additionally adjusted for ethnicity (white or other), BMI (<18.5, 18.5–24.9, 25–30, ≥30), menopausal status (premenopausal or postmenopausal), parity (0, 1–2, ≥3 children), smoking status (never smoked, previous smoker, current smoker), alcohol consumption (never or special occasions only, 1–3 times/mo, 1–4 times/wk, daily or almost daily), physical activity (metabolic equivalent of task <9 h/wk, 9–27 h/wk, ≥27 h/wk, unknown/missing), overall diet quality (Alternative Healthy Eating Index score <30, 30–60, ≥60), portions of fruit and vegetable intake (<5 portions/d, ≥5 portions/d), family history (yes or no), clinical indication for PPI use (GERD, gastric or duodenal ulcer, gastrointestinal bleeding), medications (multivitamin use, NSAID, aspirin, statin, ACEIs, beta-blockers, calcium-channel blockers, thiazide diuretics, metformin, antibiotic, oral steroids), comorbidities (diabetes, hypertension, hypercholesterolaemia) and postmenopausal hormone use (never, past, current menopausal hormone user, unknown/missing).

[c] assessed by multivariable-adjusted Cox proportional hazards model stratified by age and time period (in 2-year intervals) and additionally adjusted for ethnicity (white or other), BMI (<18.5, 18.5–24.9, 25–30, ≥30), smoking status (never smoked, previous smoker, current smoker), alcohol consumption (never or special occasions only, 1–3 times/mo, 1–4 times/wk, daily or almost daily), physical activity (metabolic equivalent of task <9 h/wk, 9–27 h/wk, ≥27 h/wk, unknown/missing), overall diet quality (Alternative Healthy Eating Index score <30, 30–60, ≥60), portions of fruit and vegetable intake (<5 portions/d, ≥5 portions/d), family history (yes or no), clinical indication for PPI use (GERD, gastric or duodenal ulcer, gastrointestinal bleeding), medications (multivitamin use, NSAID, aspirin, statin, ACEIs, beta-blockers, calcium-channel blockers, thiazide diuretics, metformin, antibiotic), comorbidities (diabetes, hypertension, hypercholesterolaemia).

[d] assessed by multivariable-adjusted Cox proportional hazards model stratified by age and sex, and additionally adjusted for clinical indication for PPI use (GERD, gastric or duodenal ulcer, gastrointestinal bleeding), medications (multivitamin use, NSAID, aspirin, statin, ACEIs, beta-blockers, calcium-channel blockers, thiazide diuretics, metformin), and comorbidities (diabetes, hypertension, hypercholesterolaemia).

[e] assessed by pooling the estimates of each cohort with inverse variance-weighted, random-effect meta-analyses using the method by DerSimonian and Laird.

Supplementary Table 7. Association between regular PPI use and risk of stroke

Cohorts	Cases	Person-years	IR in non-PPI users *	IR in PPI users *	M-H, Random HR [95%CI] #
UKB	11 395	5 673 711	191	305.9	1.17 [1.10, 1.25]
NHS	887	1 000 020	87.2	97.7	1.04 [0.85, 1.26]
NHS II	156	1 214 042	12.7	13.9	0.71 [0.43, 1.17]
HPFS	311	245 257	120.2	165.7	1.24 [0.91, 1.70]
CDARS	5392	5 851 825	61	156.4	1.12 [1.05, 1.19]
Combined	18 141	13 984 855	119.4	172.2	1.13 [1.06, 1.20]

Abbreviation: IR, incidence rate; M-H, Mantel-Haenszel; HR, hazard ratio; CI, confidence interval; UKB, UK Biobank; NHS, Nurses' Health Study; HPFS, Health Professionals Follow-Up Study; CDARS, Clinical Data Analysis and Reporting System.

* Per 100 000 person years.

# the estimates were assessed based on the fully adjusted models (see the footnote in eTable 6).

Supplementary Table 8. Association between regular PPI use and risk of chronic obstructive pulmonary disease

Cohorts	Cases	Person-years	IR in non-PPI users *	IR in PPI users *	M-H, Random HR [95%CI] #
UKB	12 207	5 330 482	198.5	538.2	1.34 [1.27, 1.41]
NHS	2428	856 235	270.7	366.9	1.34 [1.19, 1.51]
NHS II	1852	1 061 845	154.4	334.1	1.46 [1.29, 1.65]
HPFS	540	187 934	258.6	470.0	1.50 [1.19, 1.88]
CDARS	15 392	6 494 595	167.3	297.9	1.13 [1.06, 1.20]
Combined	32 419	13 931 091	191	329.2	1.33 [1.19, 1.47]

Abbreviation: IR, incidence rate; M-H, Mantel-Haenszel; HR, hazard ratio; CI, confidence interval; UKB, UK Biobank; NHS, Nurses' Health Study; HPFS, Health Professionals Follow-Up Study; CDARS, Clinical Data Analysis and Reporting System.

* Per 100 000 person years.

# the estimates were assessed based on the fully adjusted models (see the footnote in eTable 6).

Supplementary Table 9. Association between regular PPI use and risk of alzheimer's disease and other dementias

Cohorts	Cases	Person-years	IR in non-PPI users *	IR in PPI users *	M-H, Random HR [95%CI] #
UKB	8150	6 185 620	118.1	255.7	1.07 [1.00, 1.14]
NHS	3815	1 014 570	380.4	350.4	1.01 [0.91, 1.11]
NHS II	165	1 117 505	13.5	24.1	1.00 [0.65, 1.52]
HPFS	NA	NA	NA	NA	NA
CDARS	10 375	9 363 967	110.9	108.4	1.01 [0.88, 1.14]
Combined	22 505	17 681 662	121.8	191.8	1.04 [0.99, 1.10]

Abbreviation: IR, incidence rate; M-H, Mantel-Haenszel; HR, hazard ratio; CI, confidence interval; UKB, UK Biobank; NHS, Nurses' Health Study; HPFS, Health Professionals Follow-Up Study; CDARS, Clinical Data Analysis and Reporting System; NA, not available.

* Per 100 000 person years.

# the estimates were assessed based on the fully adjusted models (see the footnote in eTable 6).

Supplementary Table 10. Association between regular PPI use and risk of diabetes mellitus

Cohorts	Cases	Person-years	IR in non-PPI users *	IR in PPI users *	M-H, Random HR [95%CI] #
UKB	18 597	5 809 125	280.6	709.3	1.23 [1.18, 1.28]
NHS	4707	806 924	550.5	795.4	1.22 [1.12, 1.32]
NHS II	5382	1 170 549	406.8	858	1.20 [1.11, 1.28]
HPFS	740	221 525	316.4	439.4	1.11 [0.90, 1.36]
CDARS	18 605	5 812 084	211.8	454.8	1.20 [1.09, 1.31]
Combined	48 031	13 820 207	290.6	521.8	1.22 [1.18, 1.26]

Abbreviation: IR, incidence rate; M-H, Mantel-Haenszel; HR, hazard ratio; CI, confidence interval; UKB, UK Biobank; NHS, Nurses' Health Study; HPFS, Health Professionals Follow-Up Study; CDARS, Clinical Data Analysis and Reporting System.

* Per 100 000 person years.

# the estimates were assessed based on the fully adjusted models (see the footnote in eTable 6).

Supplementary Table 11. Association between regular PPI use and risk of lower respiratory infections

Cohorts	Cases	Person-years	IR in non-PPI users *	IR in PPI users *	M-H, Random HR [95%CI] #
UKB	25 821	5 938 148	388.9	866.2	1.19 [1.14, 1.23]
NHS	3185	453 358	691.5	805.4	1.23 [1.09, 1.38]
NHS II	2837	472 242	572.1	955.5	1.33 [1.17, 1.51]
HPFS	233	103 772	203.7	359.2	1.34 [0.94, 1.89]
CDARS	33 228	7 607 397	310.9	619.8	1.22 [1.14, 1.30]
Combined	65 304	14 574 917	373.7	661.6	1.21 [1.17, 1.25]

Abbreviation: IR, incidence rate; M-H, Mantel-Haenszel; HR, hazard ratio; CI, confidence interval; UKB, UK Biobank; NHS, Nurses' Health Study; HPFS, Health Professionals Follow-Up Study; CDARS, Clinical Data Analysis and Reporting System.

* Per 100 000 person years.

# the estimates were assessed based on the fully adjusted models (see the footnote in eTable 6).

Supplementary Table 12. Association between regular PPI use and risk of tracheal, bronchus, and lung cancer

Cohorts	Cases	Person-years	IR in non-PPI users *	IR in PPI users *	M-H, Random HR [95%CI] #
UKB	3459	5 601 855	55.8	118.0	1.17 [1.06, 1.30]
NHS	729	839 900	85.8	93.1	1.11 [0.89, 1.38]
NHS II	162	1 156 511	12.4	25.2	1.45 [0.96, 2.20]
HPFS	60	195 325	26.2	59.0	2.15 [1.12, 4.15]
CDARS	3210	7 952 280	42.6	38.9	0.96 [0.84, 1.08]
Combined	7620	15 745 871	49	47.3	1.15 [0.98, 1.35]

Abbreviation: IR, incidence rate; M-H, Mantel-Haenszel; HR, hazard ratio; CI, confidence interval; UKB, UK Biobank; NHS, Nurses' Health Study; HPFS, Health Professionals Follow-Up Study; CDARS, Clinical Data Analysis and Reporting System.

* Per 100 000 person years.

# the estimates were assessed based on the fully adjusted models (see the footnote in eTable 6).

Supplementary Table 13. Association between regular PPI use and risk of falls

Cohorts	Cases	Person-years	IR in non-PPI users *	IR in PPI users *	M-H, Random HR [95%CI] #
UKB	29 571	5 922 853	461.4	851.6	1.16 [1.12, 1.20]
NHS	NA	NA	NA	NA	NA
NHS II	NA	NA	NA	NA	NA
HPFS	NA	NA	NA	NA	NA
CDARS	22 508	8 240 580	181.0	370.9	1.18 [1.05, 1.31]
Combined	52 079	14 163 433	337.3	431.4	1.16 [1.12, 1.20]

Abbreviation: IR, incidence rate; M-H, Mantel-Haenszel; HR, hazard ratio; CI, confidence interval; UKB, UK Biobank; NHS, Nurses' Health Study; HPFS, Health Professionals Follow-Up Study; CDARS, Clinical Data Analysis and Reporting System; NA, not available.

* Per 100 000 person years.

# the estimates were assessed based on the fully adjusted models (see the footnote in eTable 6).

Supplementary Table 14. Association between regular PPI use and risk of chronic kidney disease

Cohorts	Cases	Person-years	IR in non-PPI users *	IR in PPI users *	M-H, Random HR [95%CI] #
UKB	15 998	589 8427	236.9	607.7	1.29 [1.23, 1.35]
NHS	NA	NA	NA	NA	NA
NHS II	NA	NA	NA	NA	NA
HPFS	303	168 179	166.7	265.1	1.21 [0.88, 1.65]
CDARS	5951	7 711 979	52.8	142.9	1.47 [1.33, 1.61]
Combined	22 252	13 778 585	142.9	239.3	1.35 [1.21, 1.51]

Abbreviation: IR, incidence rate; M-H, Mantel-Haenszel; HR, hazard ratio; CI, confidence interval; UKB, UK Biobank; NHS, Nurses' Health Study; HPFS, Health Professionals Follow-Up Study; CDARS, Clinical Data Analysis and Reporting System; NA, not available.

[*] Per 100 000 person years.

[#] the estimates were assessed based on the fully adjusted models (see the footnote in eTable 6).

Supplementary Table 15. Association between regular PPI use and risk of age-related hearing loss

Cohorts	Cases	Person-years	IR in non-PPI users *	IR in PPI users *	M-H, Random HR [95%CI] #
UKB	NA	NA	NA	NA	NA
NHS	NA	NA	NA	NA	NA
NHS II	NA	NA	NA	NA	NA
HPFS	NA	NA	NA	NA	NA
CDARS	8632	5 865 433	150.5	143.6	0.98 [0.84, 1.13]
Combined	8632	5 865 433	150.5	143.6	0.98 [0.84, 1.13]

Abbreviation: IR, incidence rate; M-H, Mantel-Haenszel; HR, hazard ratio; CI, confidence interval; UKB, UK Biobank; NHS, Nurses' Health Study; HPFS, Health Professionals Follow-Up Study; CDARS, Clinical Data Analysis and Reporting System; NA, not available.

* Per 100 000 person years.

# the estimates were assessed based on the fully adjusted models (see the footnote in eTable 6).

Supplementary Table 16. Association between regular PPI use and risk of hypertensive heart disease

Cohorts	Cases	Person-years	IR in non-PPI users *	IR in PPI users *	M-H, Random HR [95%CI] #
UKB	417	5 723 560	6.7	13.2	1.02 [0.75, 1.39]
NHS	NA	NA	NA	NA	NA
NHS II	NA	NA	NA	NA	NA
HPFS	NA	NA	NA	NA	NA
CDARS	571	5 849 317	9.0	11.0	1.01 [0.88, 1.14]
Combined	988	11 572 877	7.7	11.4	1.01 [0.90, 1.14]

Abbreviation: IR, incidence rate; M-H, Mantel-Haenszel; HR, hazard ratio; CI, confidence interval; UKB, UK Biobank; NHS, Nurses' Health Study; HPFS, Health Professionals Follow-Up Study; CDARS, Clinical Data Analysis and Reporting System; NA, not available.

* Per 100 000 person years.

# the estimates were assessed based on the fully adjusted models (see the footnote in eTable 6).

Supplementary Table 17. Association between regular PPI use and risk of diarrheal diseases

Cohorts	Cases	Person-years	IR in non-PPI users *	IR in PPI users *	M-H, Random HR [95%CI] #
UKB	23 217	6 078 077	339.7	776.6	1.27 [1.22, 1.32]
NHS	NA	NA	NA	NA	NA
NHS II	NA	NA	NA	NA	NA
HPFS	NA	NA	NA	NA	NA
CDARS	19 652	3 678 559	409.2	630.7	1.17 [1.08, 1.26]
Combined	42 869	9 756 636	355.4	662.9	1.20 [1.06, 1.35]

Abbreviation: IR, incidence rate; M-H, Mantel-Haenszel; HR, hazard ratio; CI, confidence interval; UKB, UK Biobank; NHS, Nurses' Health Study; HPFS, Health Professionals Follow-Up Study; CDARS, Clinical Data Analysis and Reporting System; NA, not available.

* Per 100 000 person years.

# the estimates were assessed based on the fully adjusted models (see the footnote in eTable 6).

Supplementary Table 18. Association between regular PPI use and risk of low back pain

Cohorts	Cases	Person-years	IR in non-PPI users *	IR in PPI users *	M-H, Random HR [95%CI] #
UKB	890	1 634 913	52.5	103.7	1.16 [0.86, 1.57]
NHS	NA	NA	NA	NA	NA
NHS II	NA	NA	NA	NA	NA
HPFS	NA	NA	NA	NA	NA
CDARS	NA	NA	NA	NA	NA
Combined	890	1 634 913	52.5	103.7	1.16 [0.86, 1.57]

Abbreviation: IR, incidence rate; M-H, Mantel-Haenszel; HR, hazard ratio; CI, confidence interval; UKB, UK Biobank; NHS, Nurses' Health Study; HPFS, Health Professionals Follow-Up Study; CDARS, Clinical Data Analysis and Reporting System; NA, not available.

* Per 100 000 person years.

# the estimates were assessed based on the fully adjusted models (see the footnote in eTable 6).

Supplementary Table 19. Association between regular PPI use and risk of colon and rectum cancer

Cohorts	Cases	Person-years	IR in non-PPI users *	IR in PPI users *	M-H, Random HR [95%CI] #
UKB	4972	5 601 855	87.6	99.6	0.94 [0.86, 1.03]
NHS	791	838 771	97.6	74.7	0.79 [0.62, 1.01]
NHS II	331	1 155 983	29.0	25.9	0.90 [0.62, 1.31]
HPFS	74	92 510	79.9	80.4	0.82 [0.40, 1.68]
CDARS	3222	8 863 789	30.7	40.9	1.03 [0.87, 1.19]
Combined	9390	16 552 908	61.9	46.9	0.94 [0.88, 1.01]

Abbreviation: IR, incidence rate; M-H, Mantel-Haenszel; HR, hazard ratio; CI, confidence interval; UKB, UK Biobank; NHS, Nurses' Health Study; HPFS, Health Professionals Follow-Up Study; CDARS, Clinical Data Analysis and Reporting System.

* Per 100 000 person years.

# the estimates were assessed based on the fully adjusted models (see the footnote in eTable 6).

Supplementary Table 20. Association between regular PPI use and risk of blindness and vision loss

Cohorts	Cases	Person-years	IR in non-PPI users *	IR in PPI users *	M-H, Random HR [95%CI] #
UKB	2172	6 125 827	31.7	69.4	1.11 [0.98, 1.26]
NHS	NA	NA	NA	NA	NA
NHS II	NA	NA	NA	NA	NA
HPFS	NA	NA	NA	NA	NA
CDARS	NA	NA	NA	NA	NA
Combined	2172	6 125 827	31.7	69.4	1.11 [0.98, 1.26]

Abbreviation: IR, incidence rate; M-H, Mantel-Haenszel; HR, hazard ratio; CI, confidence interval; UKB, UK Biobank; NHS, Nurses' Health Study; HPFS, Health Professionals Follow-Up Study; CDARS, Clinical Data Analysis and Reporting System; NA, not available.

* Per 100 000 person years.

# the estimates were assessed based on the fully adjusted models (see the footnote in eTable 6).

Supplementary Table 21. Association between regular PPI use and risk of atrial fibrillation and flutter

Cohorts	Cases	Person-years	IR in non-PPI users *	IR in PPI users *	M-H, Random HR [95%CI] #
UKB	22 613	5 647 821	376.8	652.5	1.10 [1.05, 1.15]
NHS	10 876	8 985 82	1158.5	1528.3	1.12 [1.06, 1.19]
NHS II	2179	1 095 712	179.0	343.7	1.18 [1.05, 1.32]
HPFS	874	66 795	1276.4	1505.3	1.09 [0.89,1.34]
CDARS	16 982	5 976 212	201.7	371.7	1.23 [1.09, 1.37]
Combined	53 524	13 685 122	369.4	450.8	1.12 [1.09, 1.16]

Abbreviation: IR, incidence rate; M-H, Mantel-Haenszel; HR, hazard ratio; CI, confidence interval; UKB, UK Biobank; NHS, Nurses' Health Study; HPFS, Health Professionals Follow-Up Study; CDARS, Clinical Data Analysis and Reporting System.

* Per 100 000 person years.

# the estimates were assessed based on the fully adjusted models (see the footnote in eTable 6).

Supplementary Table 22. Association between regular PPI use and risk of stomach cancer

Cohorts	Cases	Person-years	IR in non-PPI users *	IR in PPI users *	M-H, Random HR [95%CI] #
UKB	633	5 601 855	10.6	18.2	1.14 [0.89, 1.48]
NHS	65	764 171	8.5	8.7	1.03 [0.48, 2.23]
NHS II	NA	NA	NA	NA	NA
HPFS	16	176 112	8.6	12.4	1.52 [0.34, 6.82]
CDARS	6121	7 977 048	40.2	122.2	2.47 [2.38, 2.56]
Combined	6835	14 519 186	23.1	105.5	1.50 [0.82, 2.75]

Abbreviation: IR, incidence rate; M-H, Mantel-Haenszel; HR, hazard ratio; CI, confidence interval; UKB, UK Biobank; NHS, Nurses' Health Study; HPFS, Health Professionals Follow-Up Study; CDARS, Clinical Data Analysis and Reporting System; NA, not available.

* Per 100 000 person years.

# the estimates were assessed based on the fully adjusted models (see the footnote in eTable 6).

Supplementary Table 23. Association between regular PPI use and risk of prostate cancer

Cohorts	Cases	Person-years	IR in non-PPI users *	IR in PPI users *	M-H, Random HR [95%CI] #
UKB	9251	2 546 316	359.4	401.5	0.94 [0.87, 1.02]
NHS	NA	NA	NA	NA	NA
NHS II	NA	NA	NA	NA	NA
HPFS	1757	210 754	836.2	818.2	1.01 [0.87, 1.17]
CDARS	19 827	5 299 378	408.3	339.6	1.06 [0.93, 1.19]
Combined	30 835	8 056 448	401.4	349.6	0.99 [0.92, 1.07]

Abbreviation: IR, incidence rate; M-H, Mantel-Haenszel; HR, hazard ratio; CI, confidence interval; UKB, UK Biobank; NHS, Nurses' Health Study; HPFS, Health Professionals Follow-Up Study; CDARS, Clinical Data Analysis and Reporting System; NA, not available.

* Per 100 000 person years.

# the estimates were assessed based on the fully adjusted models (see the footnote in eTable 6).

Supplementary Table 24. Association between regular PPI use and risk of cirrhosis and other chronic liver diseases

Cohorts	Cases	Person-years	IR in non-PPI users *	IR in PPI users *	M-H, Random HR [95%CI] #
UKB	11 321	6 109 979	164.0	382.8	1.28 [1.21,1.35]
NHS	NA	NA	NA	NA	NA
NHS II	3727	1 061 447	296.6	769.7	1.39 [1.28,1.51]
HPFS	NA	NA	NA	NA	NA
CDARS	27 019	7 206 326	290.9	445.8	2.05 [1.93, 2.20]
Combined	42 067	14 377 752	219.7	446.3	1.54 [1.14, 2.08]

Abbreviation: IR, incidence rate; M-H, Mantel-Haenszel; HR, hazard ratio; CI, confidence interval; UKB, UK Biobank; NHS, Nurses' Health Study; HPFS, Health Professionals Follow-Up Study; CDARS, Clinical Data Analysis and Reporting System; NA, not available.
* Per 100 000 person years.
# the estimates were assessed based on the fully adjusted models (see the footnote in eTable 6).

Supplementary Table 25. Association between regular PPI use and risk of parkinson's disease

Cohorts	Cases	Person-years	IR in non-PPI users *	IR in PPI users *	M-H, Random HR [95%CI] #
UKB	2784	6 189 793	41.7	74.7	1.13 [1.00,1.27]
NHS	880	800 338	104.7	145.1	1.26 [1.04,1.52]
NHS II	144	945 857	13.1	32.3	1.43 [0.93,2.21]
HPFS	507	288	187.7	288.0	1.41 [1.11,1.79]
CDARS	9855	7 283 940	114.0	164.2	1.09 [1.02, 1.16]
Combined	14 170	15 220 216	72.4	147.4	1.17 [1.07,1.28]

Abbreviation: IR, incidence rate; M-H, Mantel-Haenszel; HR, hazard ratio; CI, confidence interval; UKB, UK Biobank; NHS, Nurses' Health Study; HPFS, Health Professionals Follow-Up Study; CDARS, Clinical Data Analysis and Reporting System.

* Per 100 000 person years.

# the estimates were assessed based on the fully adjusted models (see the footnote in eTable 6).

Supplementary Table 26. Association between regular PPI use and risk of osteoarthritis

Cohorts	Cases	Person-years	IR in non-PPI users *	IR in PPI users *	M-H, Random HR [95%CI] #
UKB	43 514	4 823 422	842.5	1647.9	1.27 [1.23,1.32]
NHS	6656	429 893	1548.1	1550.4	1.05 [0.96,1.15]
NHS II	NA	NA	NA	NA	NA
HPFS	NA	NA	NA	NA	NA
CDARS	36 099	5 490 890	509.0	789.9	1.66 [1.53, 1.79]
Combined	86 269	10 744 205	763.5	892.0	1.30 [1.06,1.61]

Abbreviation: IR, incidence rate; M-H, Mantel-Haenszel; HR, hazard ratio; CI, confidence interval; UKB, UK Biobank; NHS, Nurses' Health Study; HPFS, Health Professionals Follow-Up Study; CDARS, Clinical Data Analysis and Reporting System; NA, not available.
* Per 100 000 person years.
# the estimates were assessed based on the fully adjusted models (see the footnote in eTable 6).

Supplementary Table 27. Association between regular PPI use and risk of tuberculosis

Cohorts	Cases	Person-years	IR in non-PPI users *	IR in PPI users *	M-H, Random HR [95%CI] #
UKB	210	6 205 985	3.1	5.5	0.91 [0.59,1.40]
NHS	NA	NA	NA	NA	NA
NHS II	NA	NA	NA	NA	NA
HPFS	NA	NA	NA	NA	NA
CDARS	289	7 048 153	3.6	4.5	1.01 [0.94, 1.08]
Combined	499	13 254 138	3.3	4.6	1.01 [0.94,1.08]

Abbreviation: IR, incidence rate; M-H, Mantel-Haenszel; HR, hazard ratio; CI, confidence interval; UKB, UK Biobank; NHS, Nurses' Health Study; HPFS, Health Professionals Follow-Up Study; CDARS, Clinical Data Analysis and Reporting System; NA, not available.

* Per 100 000 person years.

# the estimates were assessed based on the fully adjusted models (see the footnote in eTable 6).

Supplementary Table 28. Association between regular PPI use and risk of asthma

Cohorts	Cases	Person-years	IR in non-PPI users *	IR in PPI users *	M-H, Random HR [95%CI] #
UKB	10 684	5 316 075	181.2	401.2	1.36 [1.28,1.44]
NHS	2396	854 166	256.9	435.6	1.49 [1.33,1.66]
NHS II	4931	1 023 637	449.4	750.2	1.36 [1.25,1.47]
HPFS	387	188 429	186.6	324.9	1.40 [1.07,1.84]
CDARS	21 219	4 710 993	408.5	497.9	1.08 [1.02, 1.14]
Combined	39 617	12 093 300	276.3	487.7	1.32 [1.16,1.51]

Abbreviation: IR, incidence rate; M-H, Mantel-Haenszel; HR, hazard ratio; CI, confidence interval; UKB, UK Biobank; NHS, Nurses' Health Study; HPFS, Health Professionals Follow-Up Study; CDARS, Clinical Data Analysis and Reporting System.

* Per 100 000 person years.

# the estimates were assessed based on the fully adjusted models (see the footnote in eTable 6).

Supplementary Table 29. Association between regular PPI use and risk of road injuries

Cohorts	Cases	Person-years	IR in non-PPI users *	IR in PPI users *	M-H, Random HR [95%CI] #
UKB	4593	6 133 714	74.9	74.3	1.02 [0.91,1.14]
NHS	NA	NA	NA	NA	NA
NHS II	NA	NA	NA	NA	NA
HPFS	NA	NA	NA	NA	NA
CDARS	NA	NA	NA	NA	NA
Combined	4593	6 133 714	74.9	74.3	1.02 [0.91,1.14]

Abbreviation: IR, incidence rate; M-H, Mantel-Haenszel; HR, hazard ratio; CI, confidence interval; UKB, UK Biobank; NHS, Nurses' Health Study; HPFS, Health Professionals Follow-Up Study; CDARS, Clinical Data Analysis and Reporting System; NA, not available.

[*] Per 100 000 person years.

[#] the estimates were assessed based on the fully adjusted models (see the footnote in eTable 6).

Supplementary Table 30. Association between regular PPI use and risk of pancreatic cancer

Cohorts	Cases	Person-years	IR in non-PPI users *	IR in PPI users *	M-H, Random HR [95%CI] #
UKB	1167	5 601 855	20.0	28.2	0.93 [0.76,1.13]
NHS	116	841 348	12.5	21.8	2.22 [1.33,3.68]
NHS II	38	1 156 812	3.3	3.5	1.33 [0.47,3.80]
HPFS	NA	NA	NA	NA	NA
CDARS	3159	8 071 533	37.5	40.3	0.93 [0.85, 1.01]
Combined	4480	15 671 548	23.6	37.8	1.10 [0.84,1.44]

Abbreviation: IR, incidence rate; M-H, Mantel-Haenszel; HR, hazard ratio; CI, confidence interval; UKB, UK Biobank; NHS, Nurses' Health Study; HPFS, Health Professionals Follow-Up Study; CDARS, Clinical Data Analysis and Reporting System; NA, not available.

* Per 100 000 person years.

# the estimates were assessed based on the fully adjusted models (see the footnote in eTable 6).

Supplementary Table 31. Association between regular PPI use and risk of depressive disorders

Cohorts	Cases	Person-years	IR in non-PPI users *	IR in PPI users *	M-H, Random HR [95%CI] #
UKB	13 352	5 421 462	219.8	514.1	1.32 [1.25, 1.39]
NHS	6480	873 255	705.6	983.4	1.32 [1.23, 1.42]
NHS II	12 949	952 950	1303.7	1863.6	1.39 [1.32, 1.47]
HPFS	NA	NA	NA	NA	NA
CDARS	26 903	7 016 563	299.3	490.2	1.87 [1.74, 2.02]
Combined	59 684	14 264 230	373.7	542.3	1.46 [1.26, 1.68]

Abbreviation: IR, incidence rate; M-H, Mantel-Haenszel; HR, hazard ratio; CI, confidence interval; UKB, UK Biobank; NHS, Nurses' Health Study; HPFS, Health Professionals Follow-Up Study; CDARS, Clinical Data Analysis and Reporting System; NA, not available.

* Per 100 000 person years.

# the estimates were assessed based on the fully adjusted models (see the footnote in eTable 6).

Supplementary Table 32. Association between regular PPI use and risk of breast cancer

Cohorts	Cases	Person-years	IR in non-PPI users *	IR in PPI users *	M-H, Random HR [95%CI] #
UKB	7491	3 055 539	244.6	250.3	0.95 [0.87, 1.04]
NHS	3890	828 583	472.1	453.4	0.91 [0.80, 1.02]
NHS II	3506	1 146 459	305.8	305.9	0.93 [0.83, 1.04]
HPFS	NA	NA	NA	NA	NA
CDARS	3772	2 698 183	137.0	142.4	1.04 [0.95, 1.13]
Combined	18 659	7 728 764	258.9	189.7	0.96 [0.91, 1.02]

Abbreviation: IR, incidence rate; M-H, Mantel-Haenszel; HR, hazard ratio; CI, confidence interval; UKB, UK Biobank; NHS, Nurses' Health Study; HPFS, Health Professionals Follow-Up Study; CDARS, Clinical Data Analysis and Reporting System; NA, not available.

* Per 100 000 person years.

# the estimates were assessed based on the fully adjusted models (see the footnote in eTable 6).

Supplementary Table 33. Association between regular PPI use and risk of esophageal cancer

Cohorts	Cases	Person-years	IR in non-PPI users *	IR in PPI users *	M-H, Random HR [95%CI] #
UKB	910	5 601 855	14.2	35.3	1.63 [1.34, 1.98]
NHS	45	764 381	5.3	9.6	1.83 [0.83, 4.03]
NHS II	NA	NA	NA	NA	NA
HPFS	15	195 432	6.5	14.8	2.69 [0.70, 10.27]
CDARS	1684	8 046 415	17.7	23.1	1.10 [1.05, 1.15]
Combined	2654	14 608 083	14.7	24	1.44 [1.02,2.03]

Abbreviation: IR, incidence rate; M-H, Mantel-Haenszel; HR, hazard ratio; CI, confidence interval; UKB, UK Biobank; NHS, Nurses' Health Study; HPFS, Health Professionals Follow-Up Study; CDARS, Clinical Data Analysis and Reporting System; NA, not available.

* Per 100 000 person years.

# the estimates were assessed based on the fully adjusted models (see the footnote in eTable 6).

Supplementary Table 34. Association between regular PPI use and risk of liver cancer

Cohorts	Cases	Person-years	IR in non-PPI users *	IR in PPI users *	M-H, Random HR [95%CI] #
UKB	551	5 601 855	8.8	19.5	1.10 [0.85, 1.41]
NHS	34	841 482	4.2	3.3	0.64 [0.21, 1.96]
NHS II	NA	NA	NA	NA	NA
HPFS	11	216 740	5.4	3.3	0.52 [0.05, 5.25]
CDARS	777	8 036 433	9.2	10.0	1.04 [0.86, 1.22]
Combined	1373	14 696 510	8.5	10.8	1.05 [0.91,1.21]

Abbreviation: IR, incidence rate; M-H, Mantel-Haenszel; HR, hazard ratio; CI, confidence interval; UKB, UK Biobank; NHS, Nurses' Health Study; HPFS, Health Professionals Follow-Up Study; CDARS, Clinical Data Analysis and Reporting System; NA, not available.

* Per 100 000 person years.

# the estimates were assessed based on the fully adjusted models (see the footnote in eTable 6).

Supplementary Table 35. Association between regular PPI use and risk of cardiomyopathy and myocarditis

Cohorts	Cases	Person-years	IR in non-PPI users *	IR in PPI users *	M-H, Random HR [95%CI] #
UKB	1133	5 900 529	18.1	30.5	1.06 [0.85,1.31]
NHS	NA	NA	NA	NA	NA
NHS II	NA	NA	NA	NA	NA
HPFS	NA	NA	NA	NA	NA
CDARS	4103	5 876 640	55.1	85.4	1.02 [0.84, 1.20]
Combined	5236	11 777 169	31.4	76.8	1.04 [0.90, 1.19]

Abbreviation: IR, incidence rate; M-H, Mantel-Haenszel; HR, hazard ratio; CI, confidence interval; UKB, UK Biobank; NHS, Nurses' Health Study; HPFS, Health Professionals Follow-Up Study; CDARS, Clinical Data Analysis and Reporting System; NA, not available.

* Per 100 000 person years.

# the estimates were assessed based on the fully adjusted models (see the footnote in eTable 6).

Supplementary Table 36. Performance of the prediction models for any of 15 unintended outcomes in derived population and validation population

C-index	Derived population	Validation population
One year	0.684	0.689
Five years	0.680	0.685
Ten years	0.673	0.683

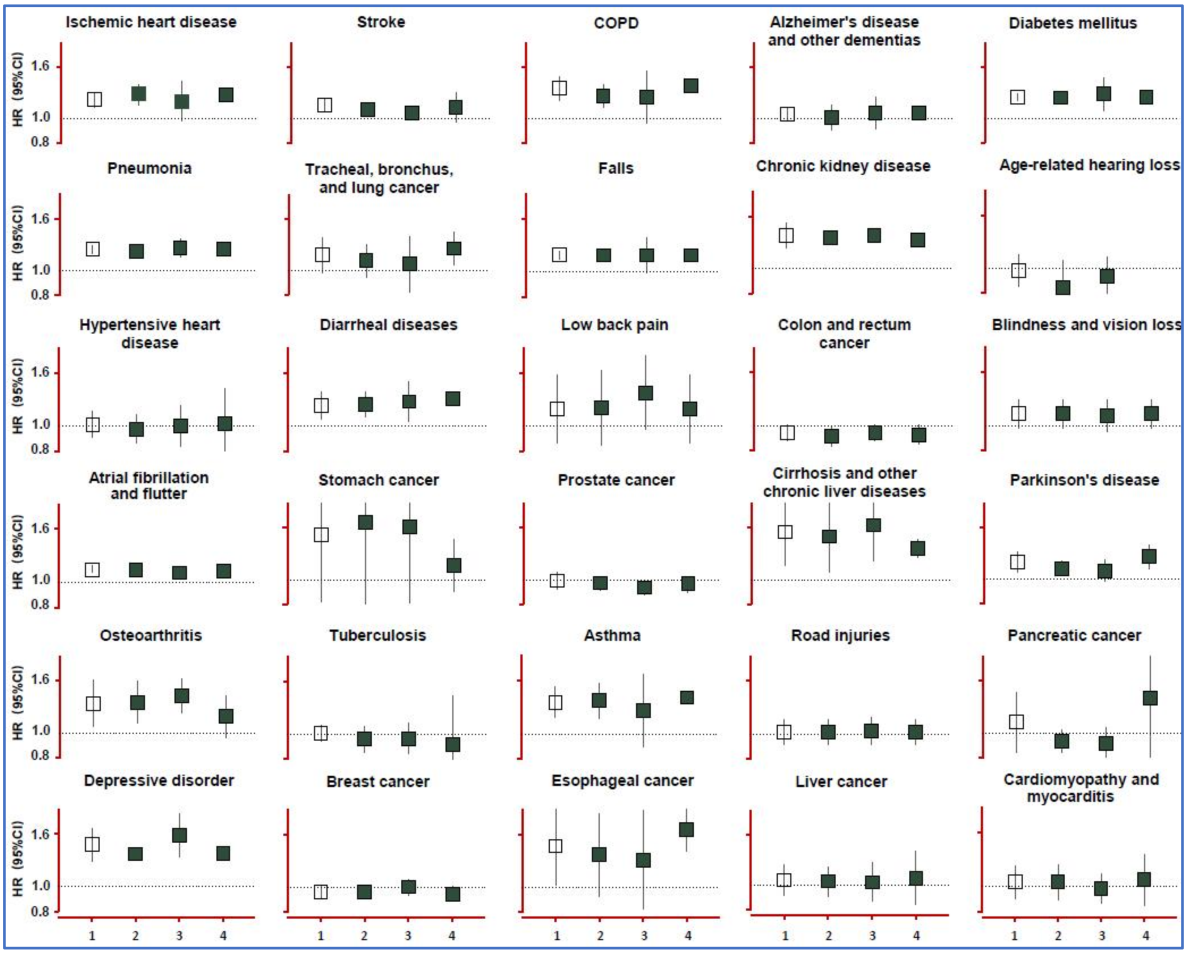

Supplementary Figure 1. Sensitive analyses for the associations between regular PPI use and risk of the major high-burden diseases

Abbreviation: COPD, chronic obstructive pulmonary disease; HR, hazard ratio; CI, confidence interval.
The estimates were assessed based on the fully adjusted models (see the footnote in eTable 6).
Analyses 1 to 4 were as follows:
(1) estimates derived from the main analyses.
(2) pooled the estimates of each cohort after lagging the exposure for 4 years;
(3) pooled the estimates from propensity score analysis using the inverse probability treatment weighting method in the UK Biobank and CDARS database;
(4) pooled the estimates of each cohort after excluding the CDARS database.
Source data are provided as a Source Data file.

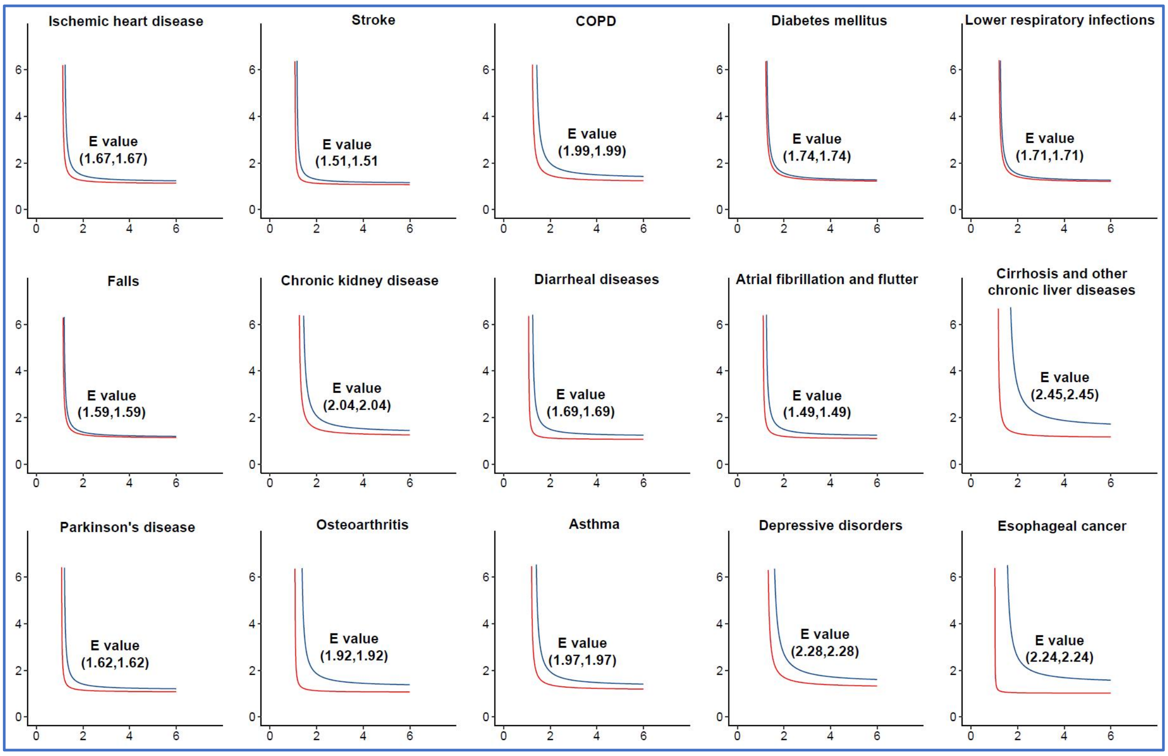

Supplementary Figure 2. Value of the joint minimum strength of association on the risk ratio scale that an unmeasured confounder must have with the exposure and the outcome to fully explain away an observed HR between PPI and the outcome.

Abbreviation: COPD, chronic obstructive pulmonary disease; PPI, proton pump inhibitor; HR, hazard ratio. The blue line represents e-values, and the red line represents the lower limit of e-values.

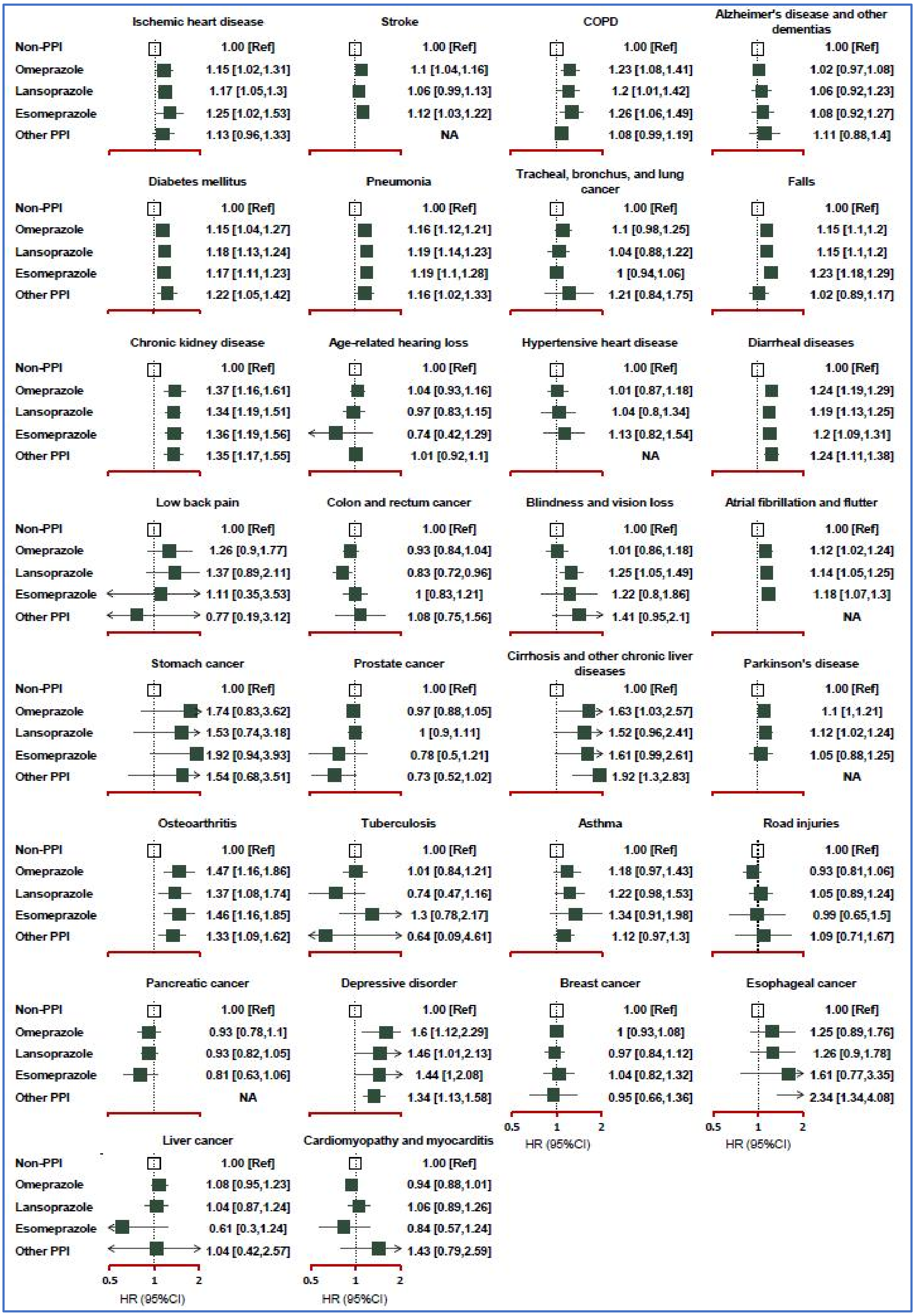

Supplementary Figure 3. Pooled hazards ratios by individual class of PPIs in UK Biobank and CDARS.

Abbreviation: COPD, chronic obstructive pulmonary disease; HR, hazard ratio; CI, confidence interval; PPI, proton pump inhibitor; NA, not available.
The estimates were assessed based on the fully adjusted models (see the footnote in eTable 6).

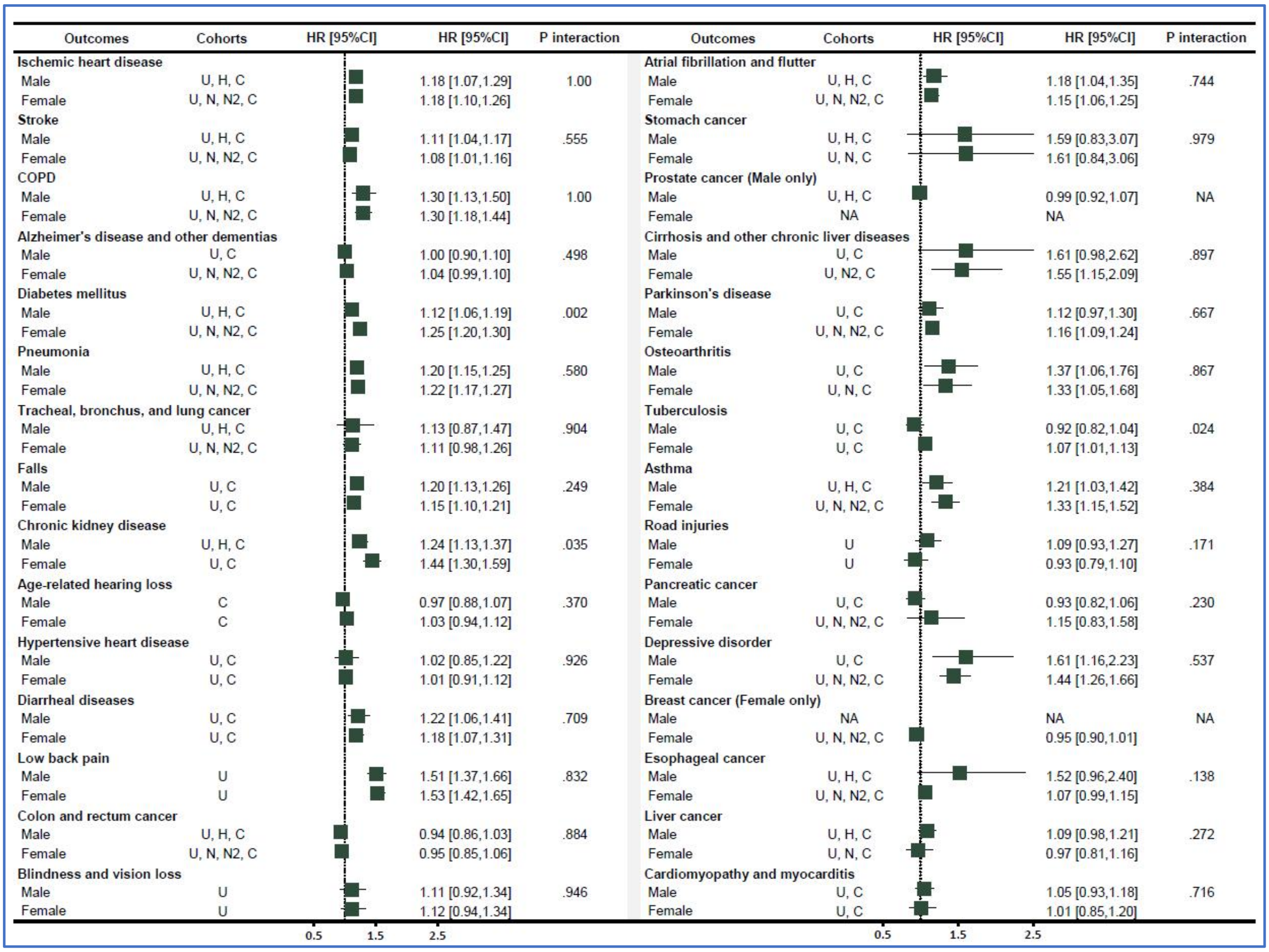

Supplementary Figure 4. Pooled associations between regular PPI use with risk of the major diseases stratified by sex

Abbreviation: U, UK Biobank; N, NHS; N2, NHS II; H, HPFS; C, CDARS; COPD, chronic obstructive pulmonary disease; PPI, proton pump inhibitor; HR, hazard ratio; CI, confidence interval; NA, not available.
The estimates were assessed based on the fully adjusted models (see the footnote in eTable 6).

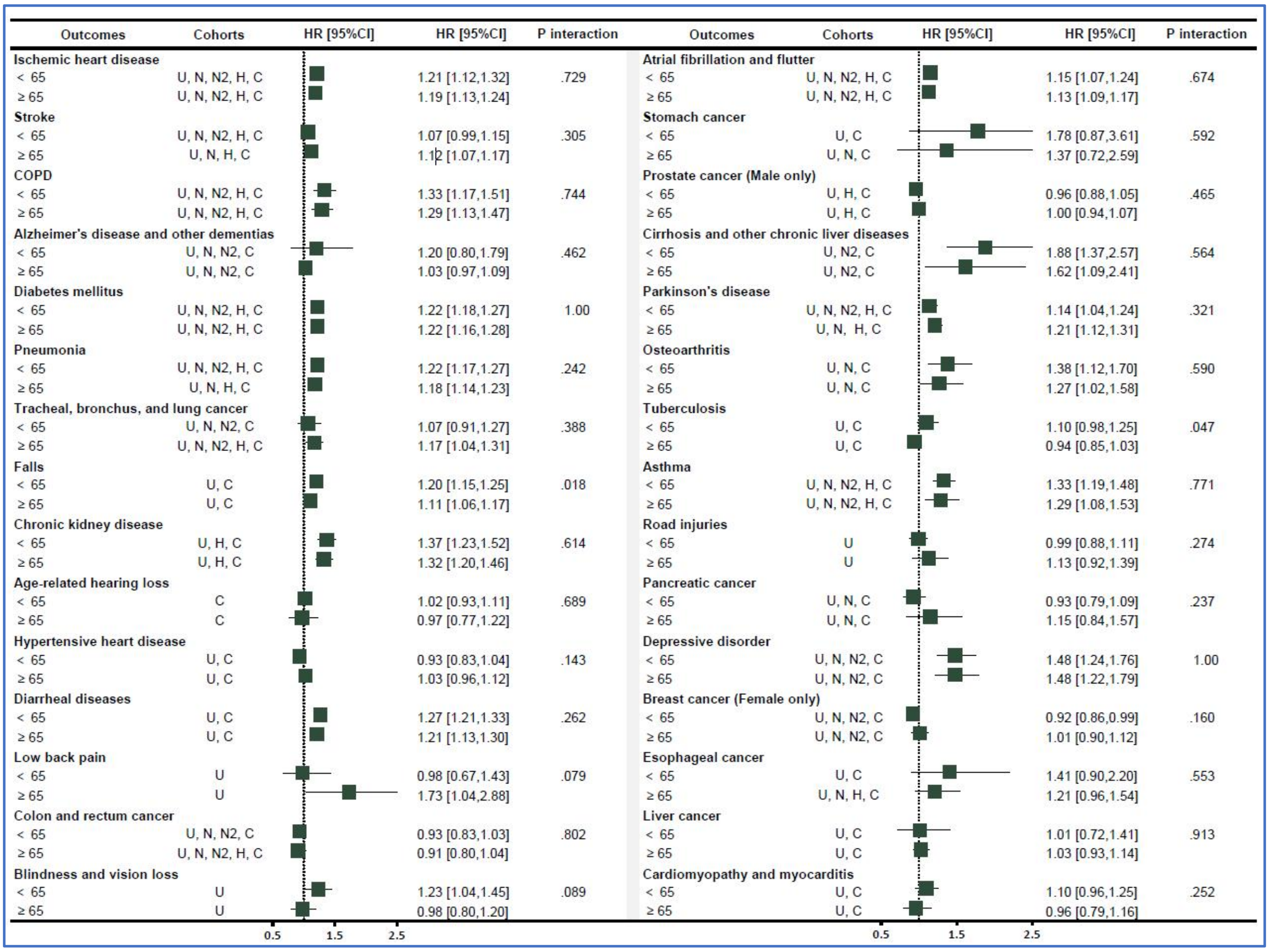

Supplementary Figure 5. Pooled associations between regular PPI use with risk of the major diseases stratified by age

Abbreviation: U, UK Biobank; N, NHS; N2, NHS II; H, HPFS; C, CDARS; COPD, chronic obstructive pulmonary disease; PPI, proton pump inhibitor; HR, hazard ratio; CI, confidence interval.
The estimates were assessed based on the fully adjusted models (see the footnote in eTable 6).

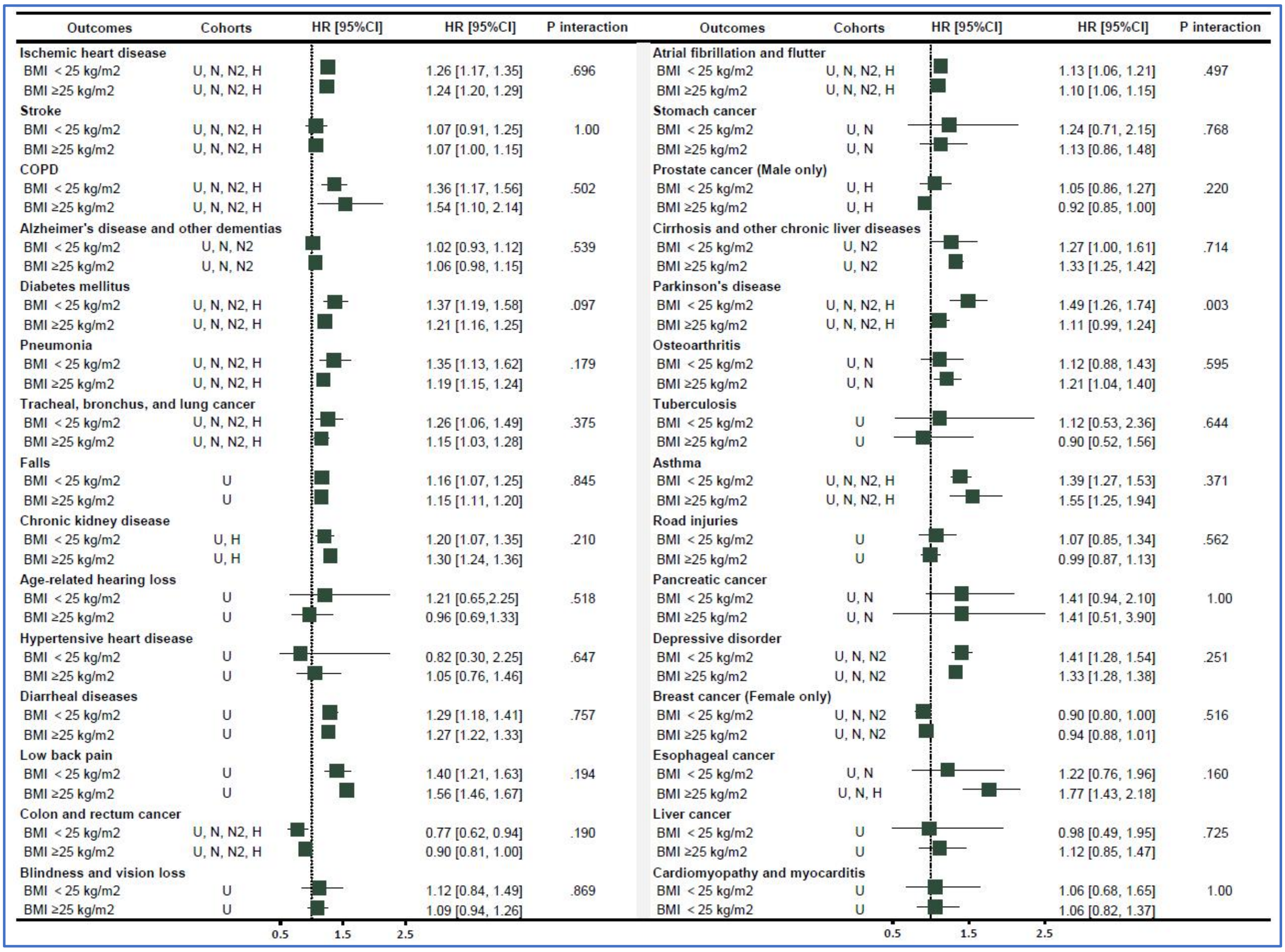

Supplementary Figure 6. Pooled associations between regular PPI use with risk of the major diseases stratified by BMI

Abbreviation: U, UK Biobank; N, NHS; N2, NHS II; H, HPFS; C, CDARS; BMI, body mass index; COPD, chronic obstructive pulmonary disease; PPI, proton pump inhibitor; HR, hazard ratio; CI, confidence interval.
The estimates were assessed based on the fully adjusted models (see the footnote in eTable 6).

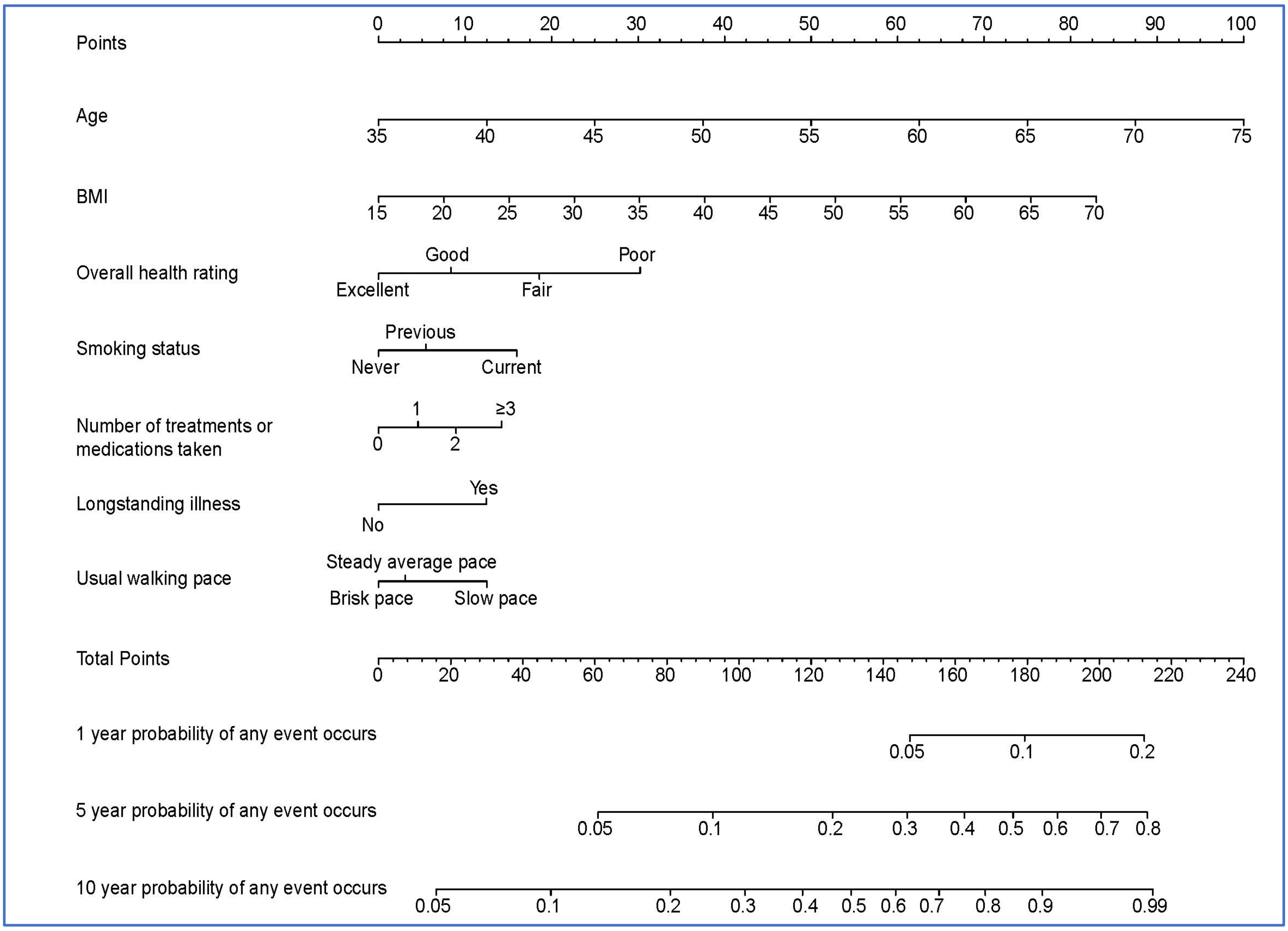

Supplementary Figure 7. Nomogram for predicting the 1-, 5- and 10-year probability of any of 15 unintended outcomes.

By drawing an upward line from the corresponding values of the seven predictors in the figure to the “points” line, the sum of all these scores, plotted on the “Total points” line, corresponds to prediction of any of 15 unintended outcomes in the “1/5/10 year probability of any event occurs” lines. Source data are provided as a Source Data file.

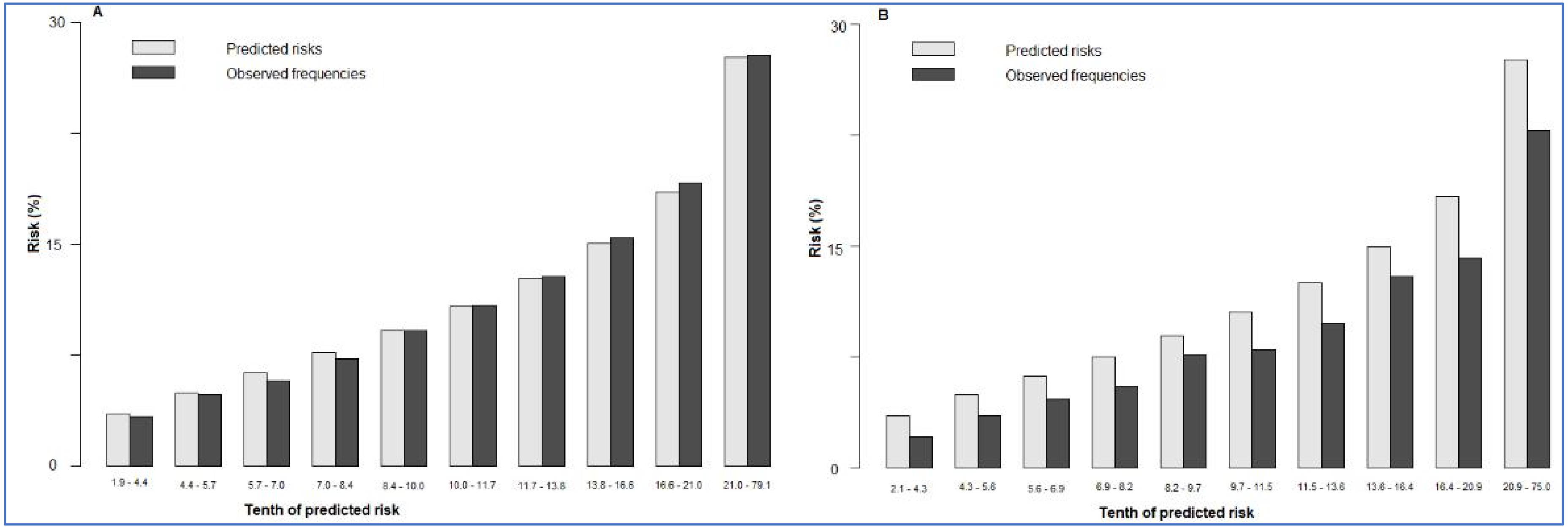

Supplementary Figure 8. Predicted and observed 10-year risk of 15 major diseases by 10th of predicted risk in derived population and validation population

A. Derived population; B. Validation population. Source data are provided as a Source Data file.

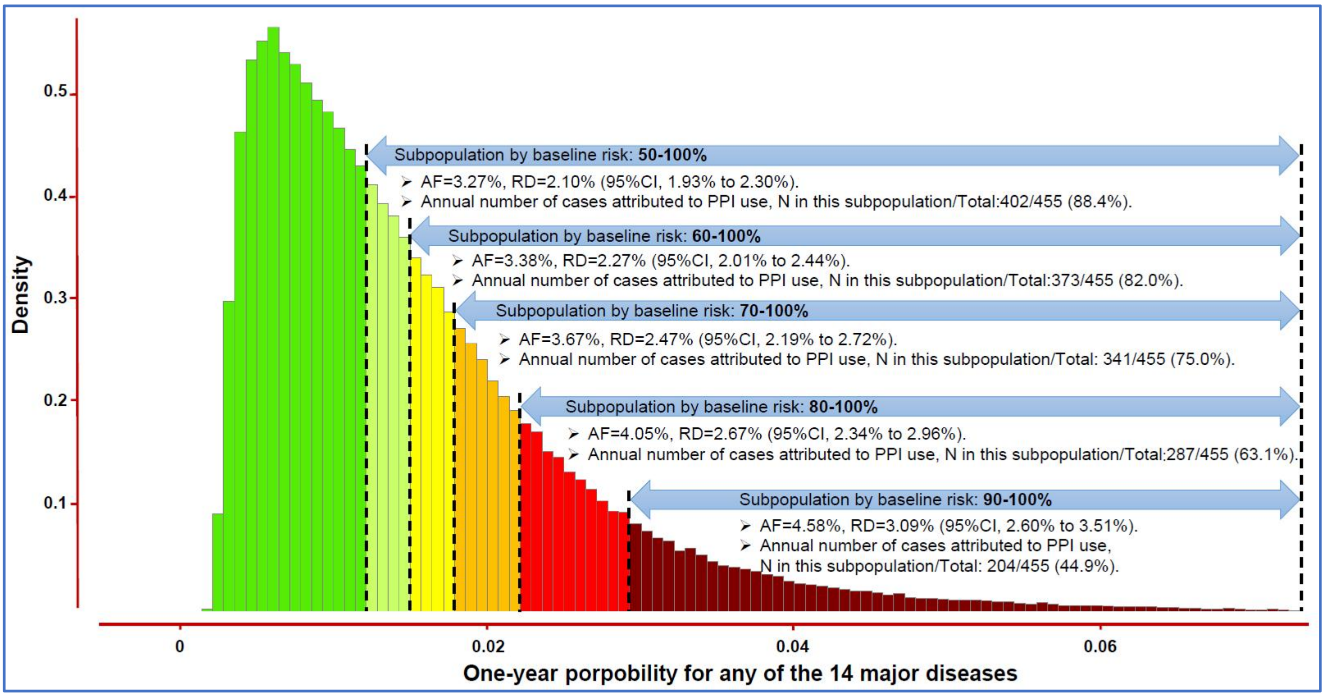

Supplementary Figure 9. Sensitivity analysis: PPI-related absolute risk for major high-burden diseases according to the distribution of the baseline predicted risk after excluding osteoarthritis.

Abbreviation: AF, attributable fraction; PPI, proton pump inhibitor; RD, risk difference.
This histogram presented the distribution of baseline predicted risk for any of the 15 PPI-related diseases. The performance and nomogram for the prediction model is available in S**upplementary Table S38 and Supplementary Fig. S7-8**. The RD and AF of PPI use for one year was calculated. Annual number of cases attributed to PPI use in each strata (i.e., n) was calculated based on attributable risk and exposure, and then summed as the total annual number of cases attributed to PPI use in all populations (i.e., total). The results showed that most cases were occurred in the individuals with high baseline predicted risk, and those who with low baseline risk do not need to be over panicked and should adhere to PPI treatment. Source data are provided as a Source Data file.

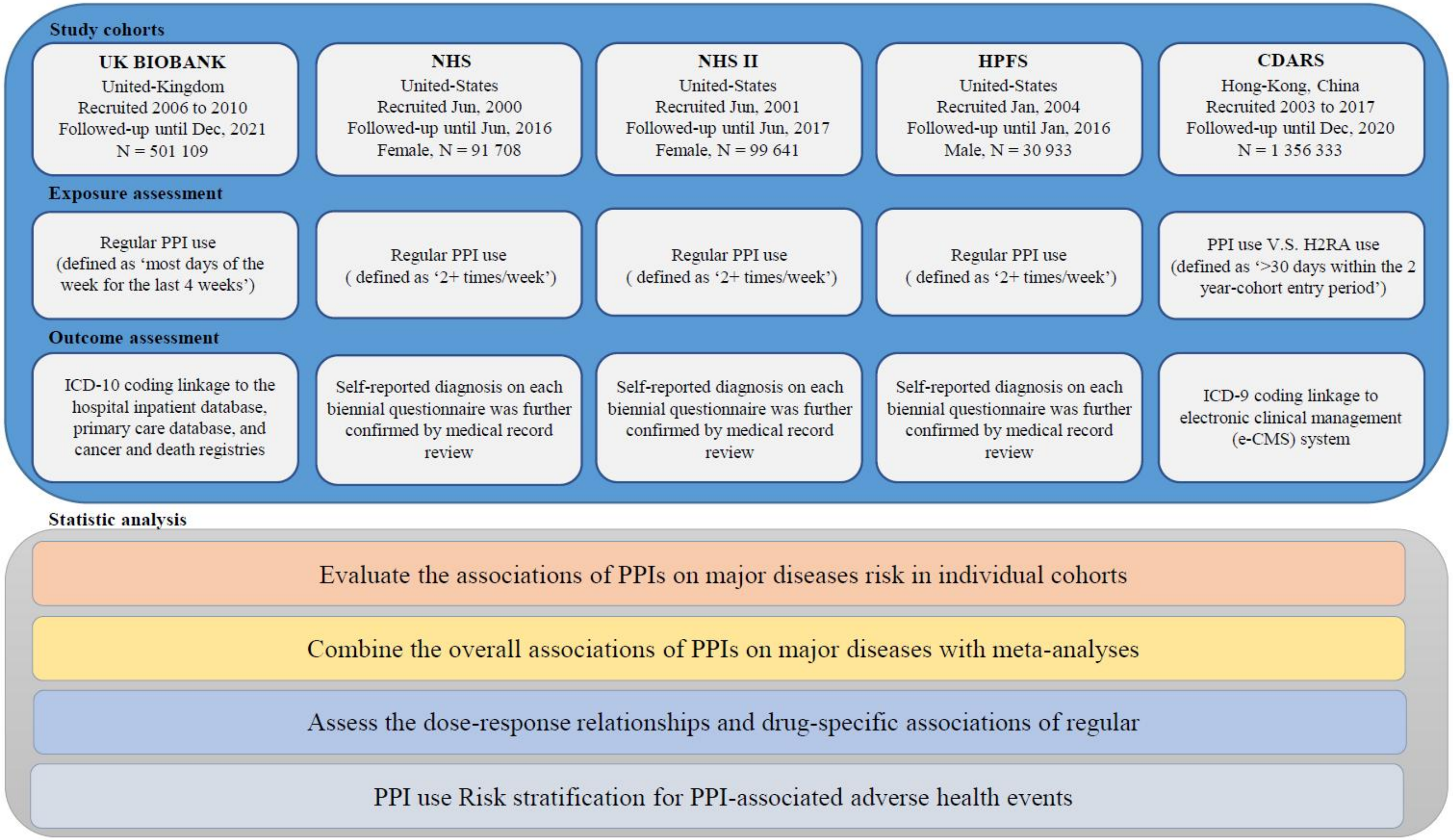

Supplementary Figure 10. Study design of the present study.

Abbreviation: PPI, proton pump inhibitor; H2RA, H2 receptor antagonist; NHS, nurses' health study; HPFS, health professionals follow-up study; CDARS, clinical data analysis and reporting system; ICD, international classification of diseases.

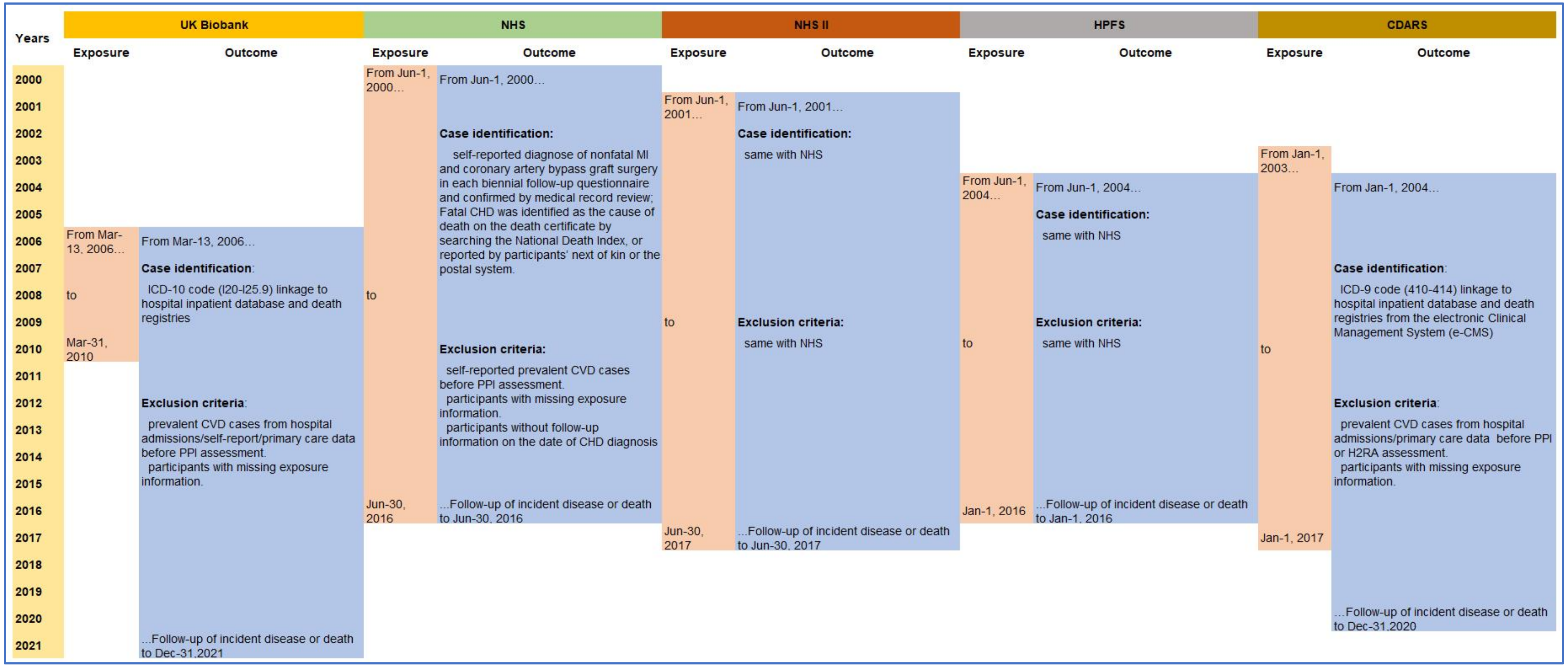

Supplementary Figure 11. Framework for ischemic heart disease in the present study.

Abbreviation: PPI, proton pump inhibitor; H2RA, H2 receptor antagonist; NHS, nurses' health study; HPFS, health professionals follow-up study; CDARS, clinical data analysis and reporting system; ICD, international classification of diseases; CVD, cardiovascular disease; CHD, coronary heart disease.

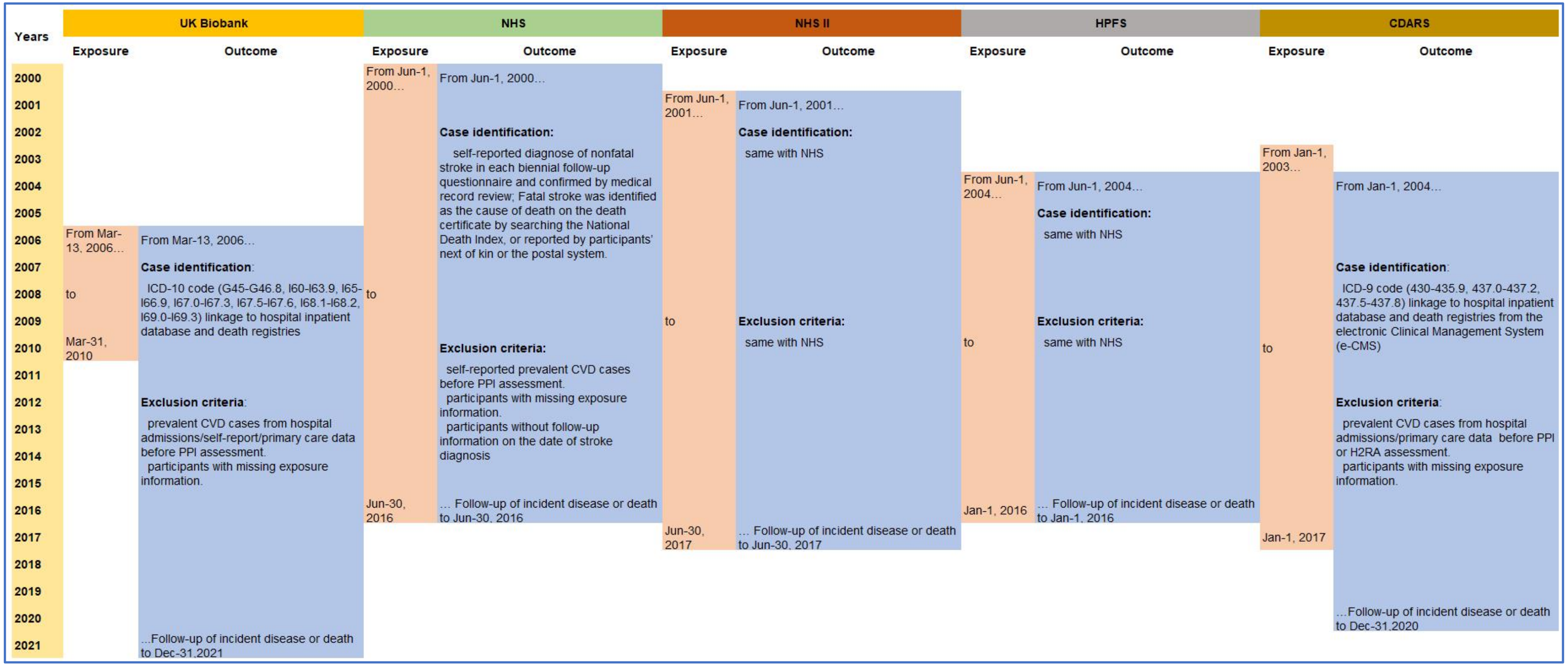

Supplementary Figure 12. Framework for stroke in the present study.

Abbreviation: PPI, proton pump inhibitor; H2RA, H2 receptor antagonist; NHS, nurses' health study; HPFS, health professionals follow-up study; CDARS, clinical data analysis and reporting system; ICD, international classification of diseases; CVD, cardiovascular disease.

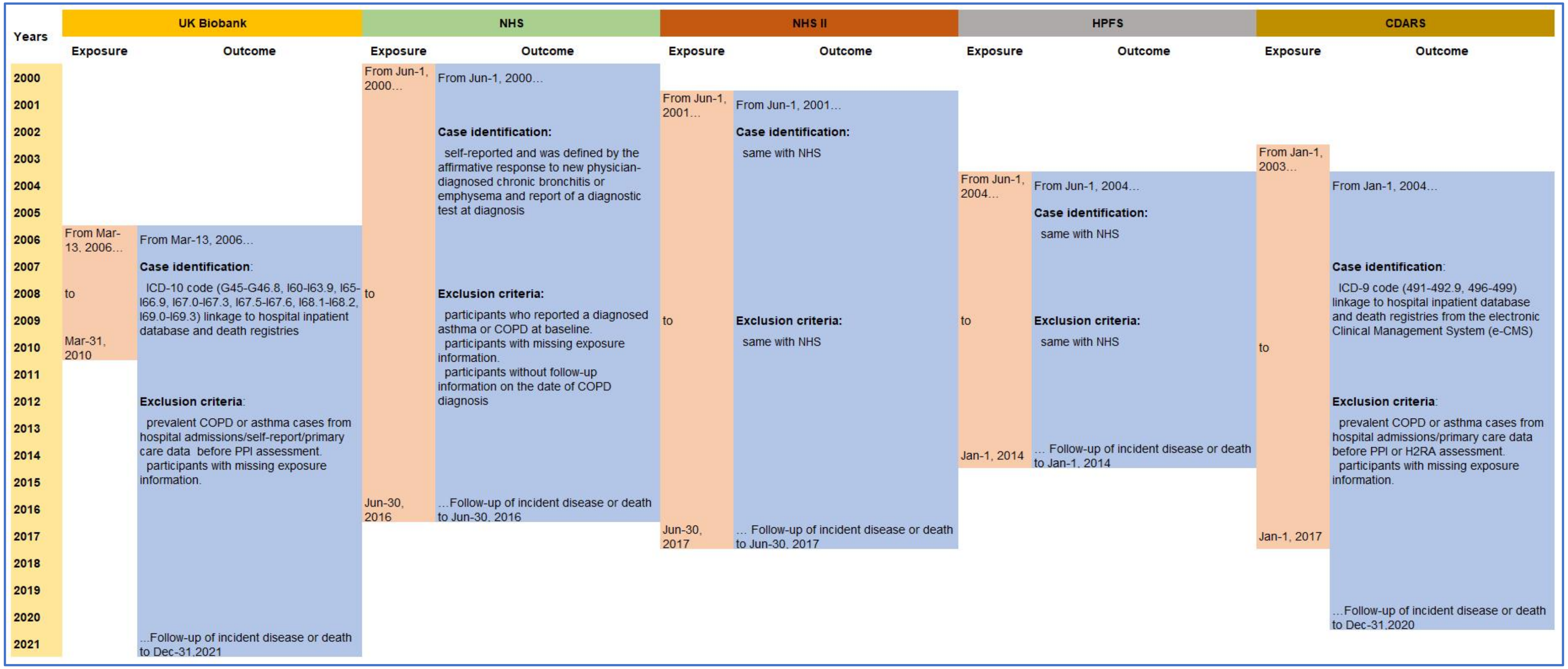

Supplementary Figure 13. Framework for chronic obstructive pulmonary disease in the present study.

Abbreviation: PPI, proton pump inhibitor; H2RA, H2 receptor antagonist; NHS, nurses' health study; HPFS, health professionals follow-up study; CDARS, clinical data analysis and reporting system; ICD, international classification of diseases; COPD, chronic obstructive pulmonary disease.

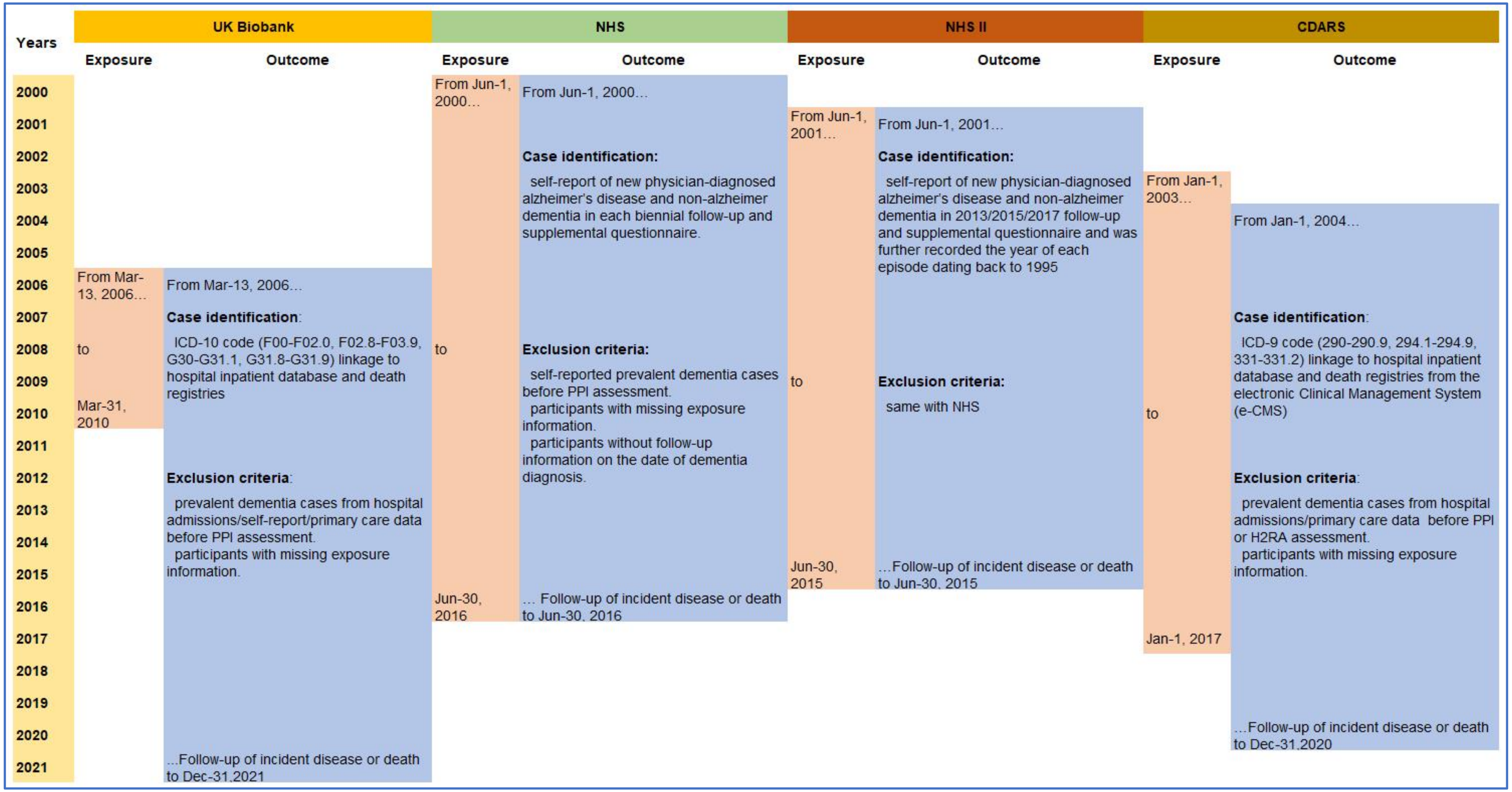

Supplementary Figure 14. Framework for alzheimer's disease and other dementias in the present study.

Abbreviation: PPI, proton pump inhibitor; H2RA, H2 receptor antagonist; NHS, nurses' health study; HPFS, health professionals follow-up study; CDARS, clinical data analysis and reporting system; ICD, international classification of diseases.

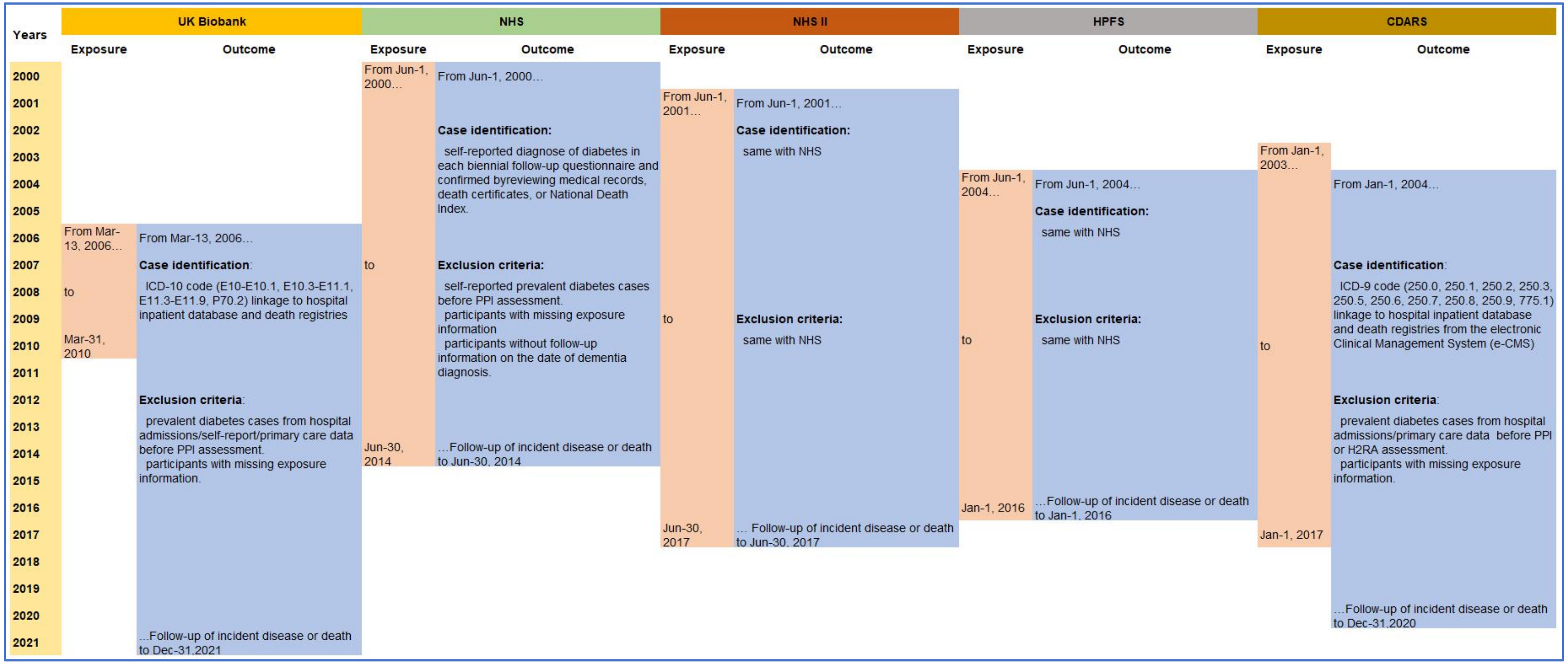

Supplementary Figure 15. Framework for diabetes mellitus in the present study.

Abbreviation: PPI, proton pump inhibitor; H2RA, H2 receptor antagonist; NHS, nurses' health study; HPFS, health professionals follow-up study; CDARS, clinical data analysis and reporting system; ICD, international classification of disease.

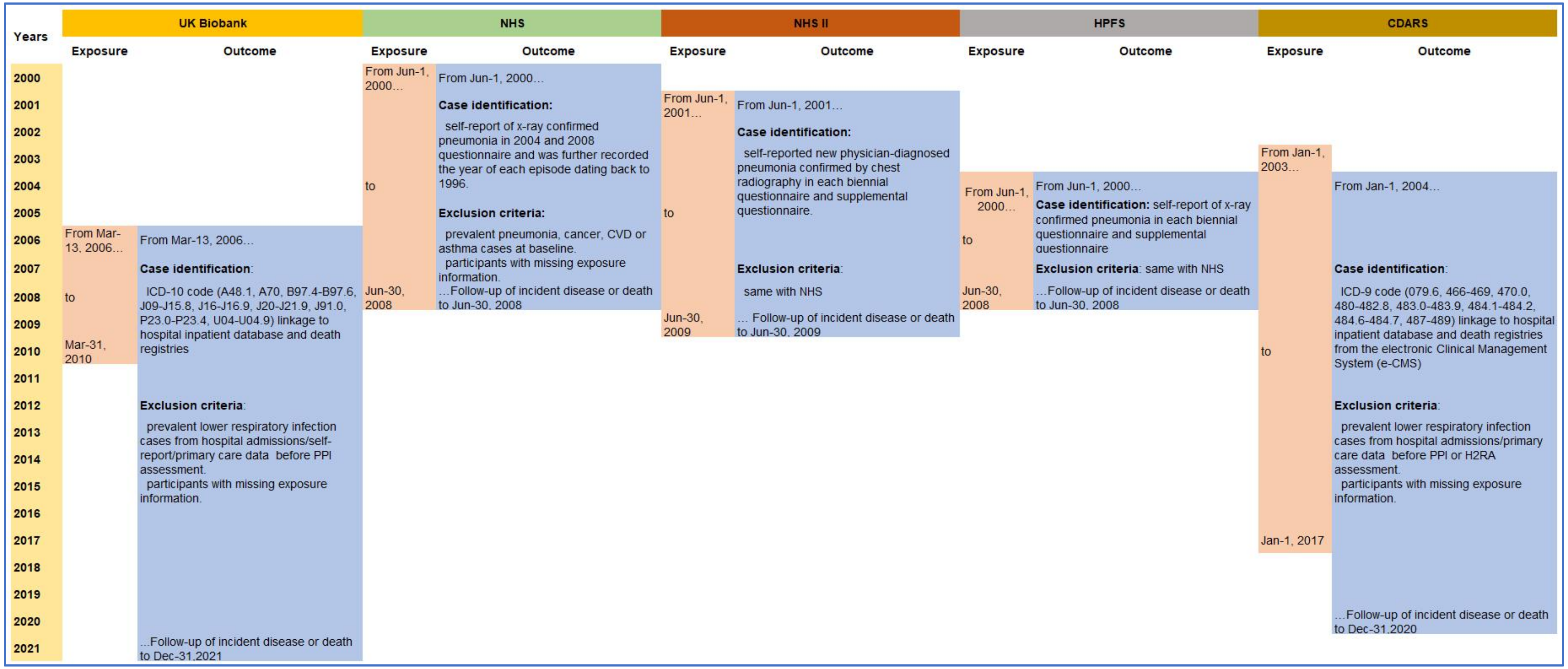

Supplementary Figure 16. Framework for lower respiratory infections in the present study.

Abbreviation: PPI, proton pump inhibitor; H2RA, H2 receptor antagonist; NHS, nurses' health study; HPFS, health professionals follow-up study; CDARS, clinical data analysis and reporting system; ICD, international classification of diseases.

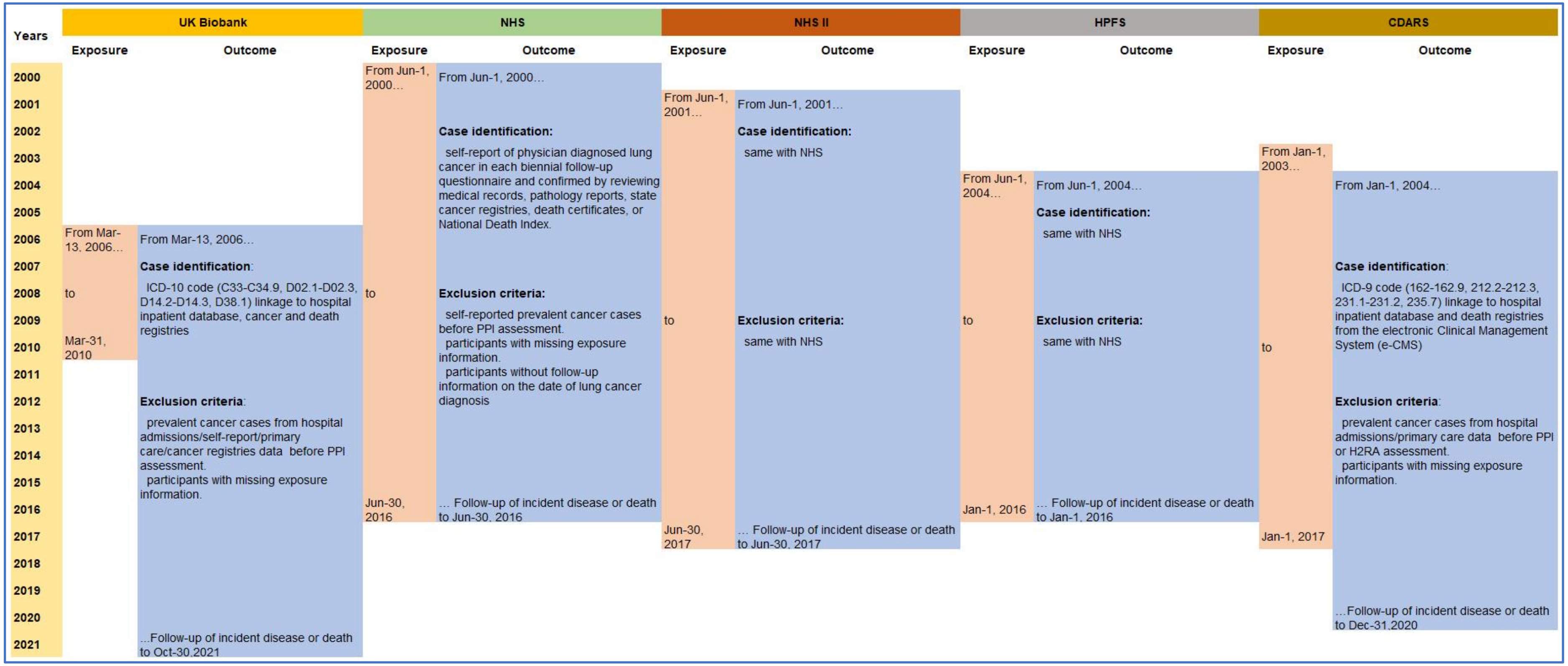

Supplementary Figure 17. Framework for tracheal, bronchus, and lung cancer in the present study.

Abbreviation: PPI, proton pump inhibitor; H2RA, H2 receptor antagonist; NHS, nurses' health study; HPFS, health professionals follow-up study; CDARS, clinical data analysis and reporting system; ICD, international classification of disease.

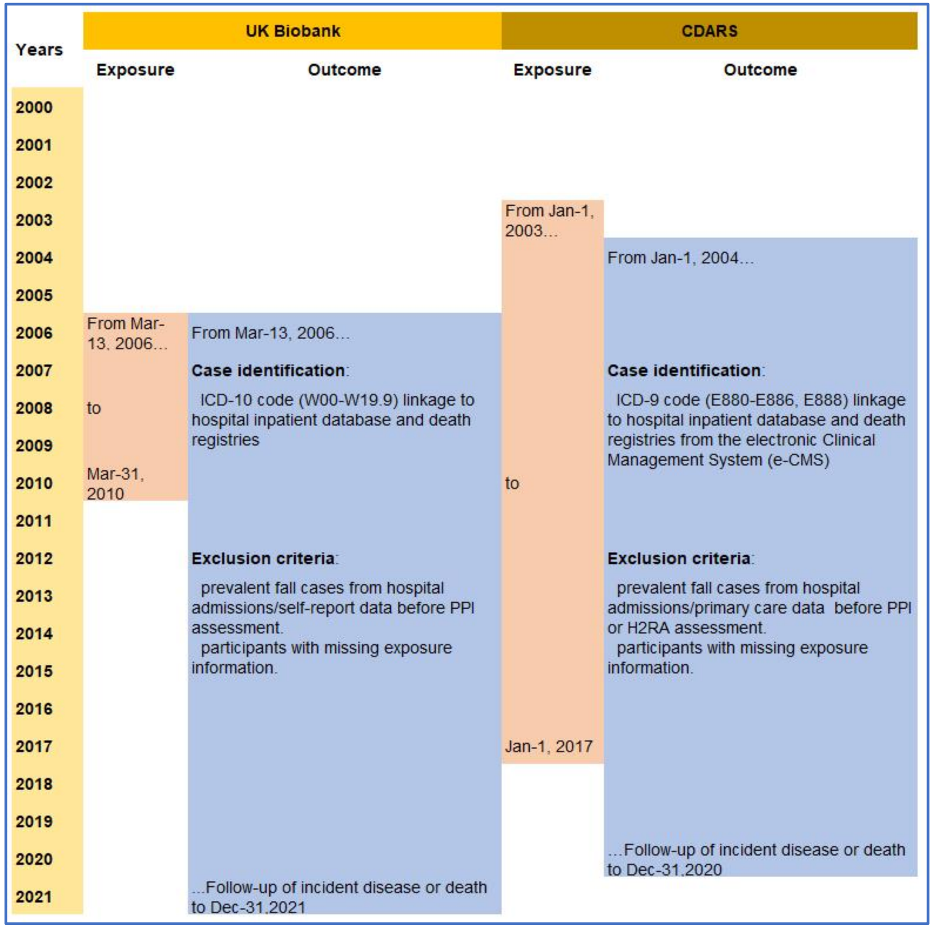

Supplementary Figure 18. Framework for falls in the present study.

Abbreviation: PPI, proton pump inhibitor; H2RA, H2 receptor antagonist; CDARS, clinical data analysis and reporting system; ICD, international classification of disease.

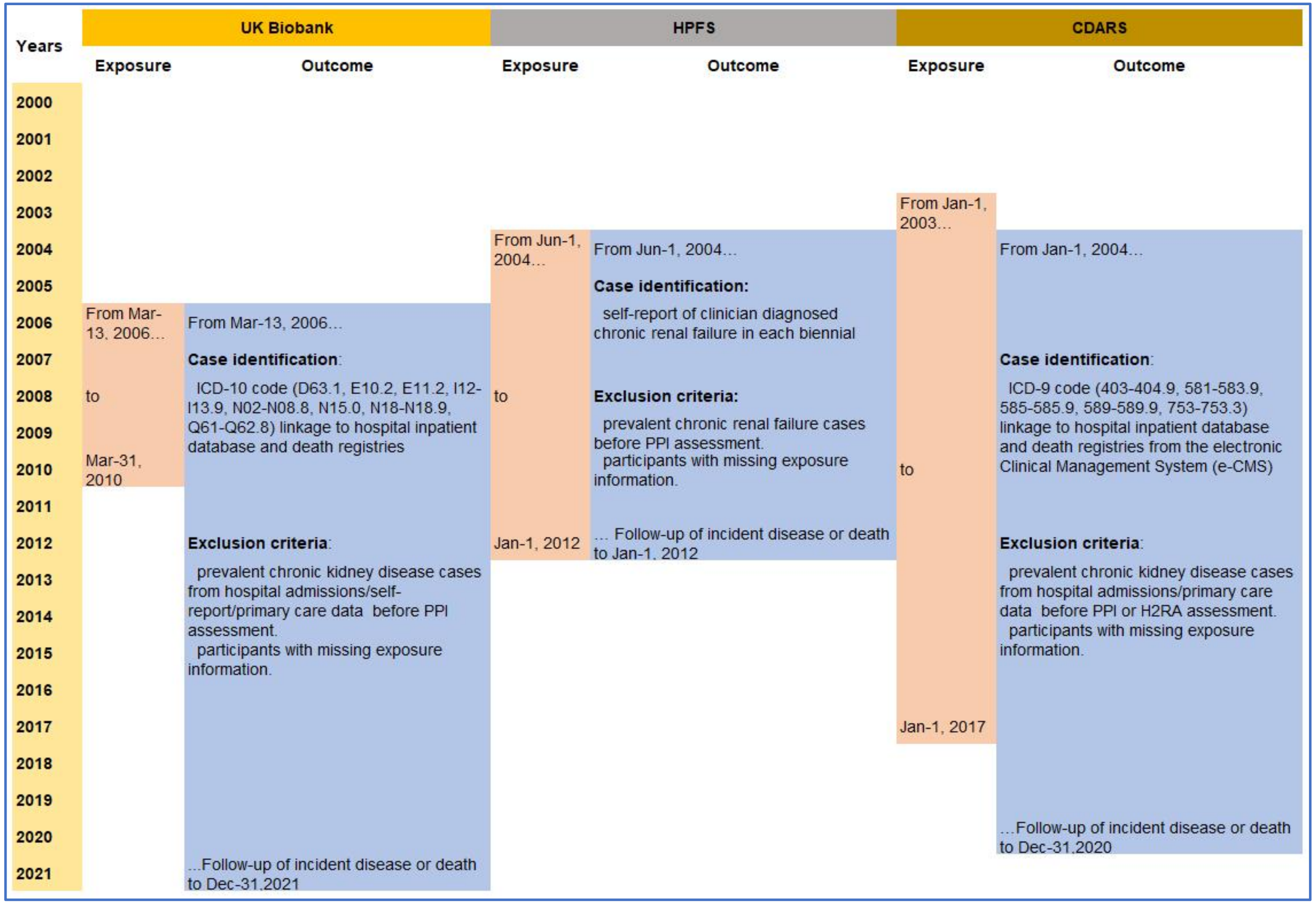

Supplementary Figure 19. Framework for chronic kidney disease in the present study.

Abbreviation: PPI, proton pump inhibitor; H2RA, H2 receptor antagonist; HPFS, health professionals follow-up study; CDARS, clinical data analysis and reporting system; ICD, international classification of disease.

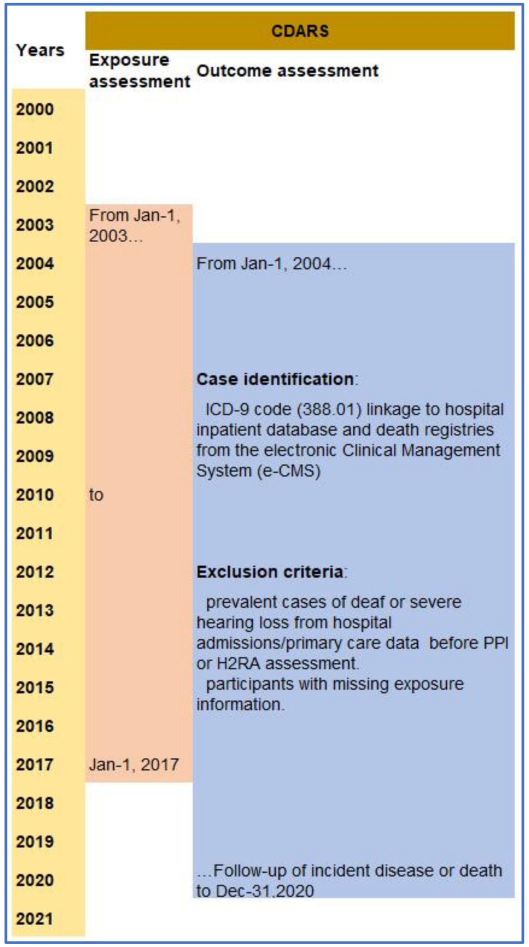

Supplementary Figure 20. Framework for age-related hearing loss in the present study.

Abbreviation: PPI, proton pump inhibitor; H2RA, H2 receptor antagonist; CDARS, clinical data analysis and reporting system; ICD, international classification of disease.

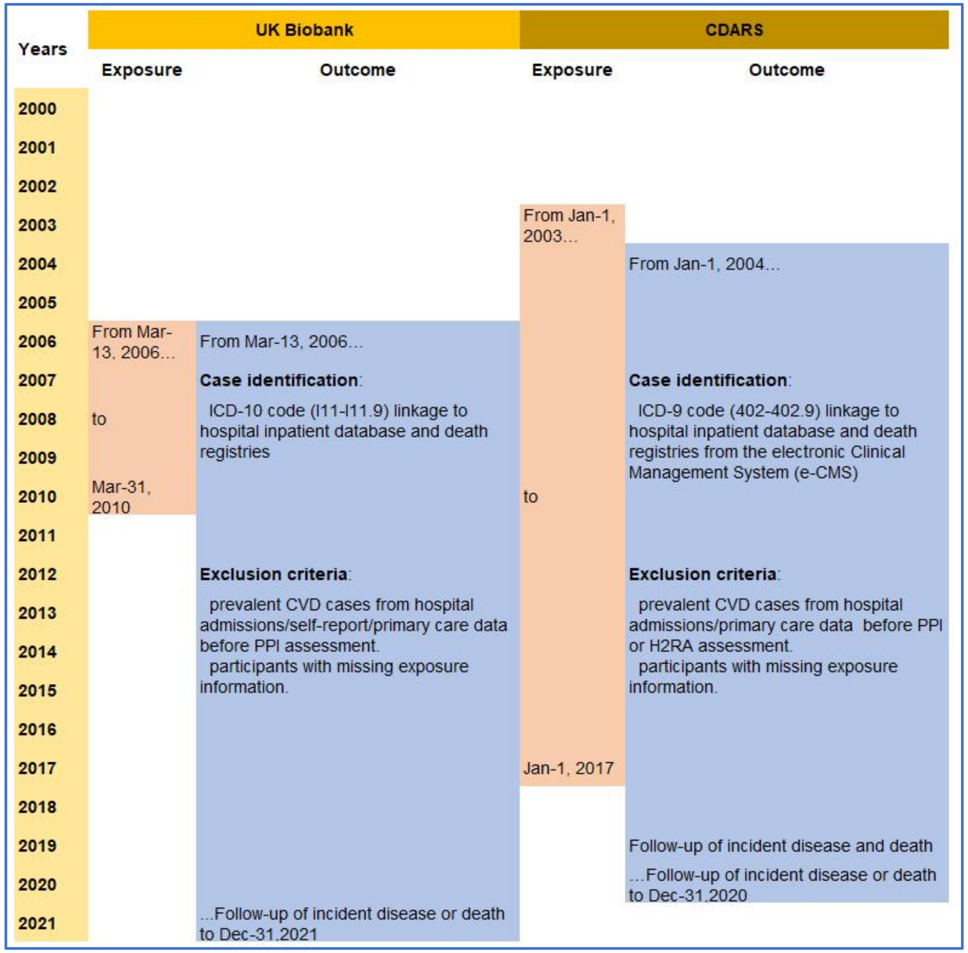

Supplementary Figure 21. Framework for hypertensive heart disease in the present study.

Abbreviation: PPI, proton pump inhibitor; H2RA, H2 receptor antagonist; CDARS, clinical data analysis and reporting system; ICD, international classification of disease.

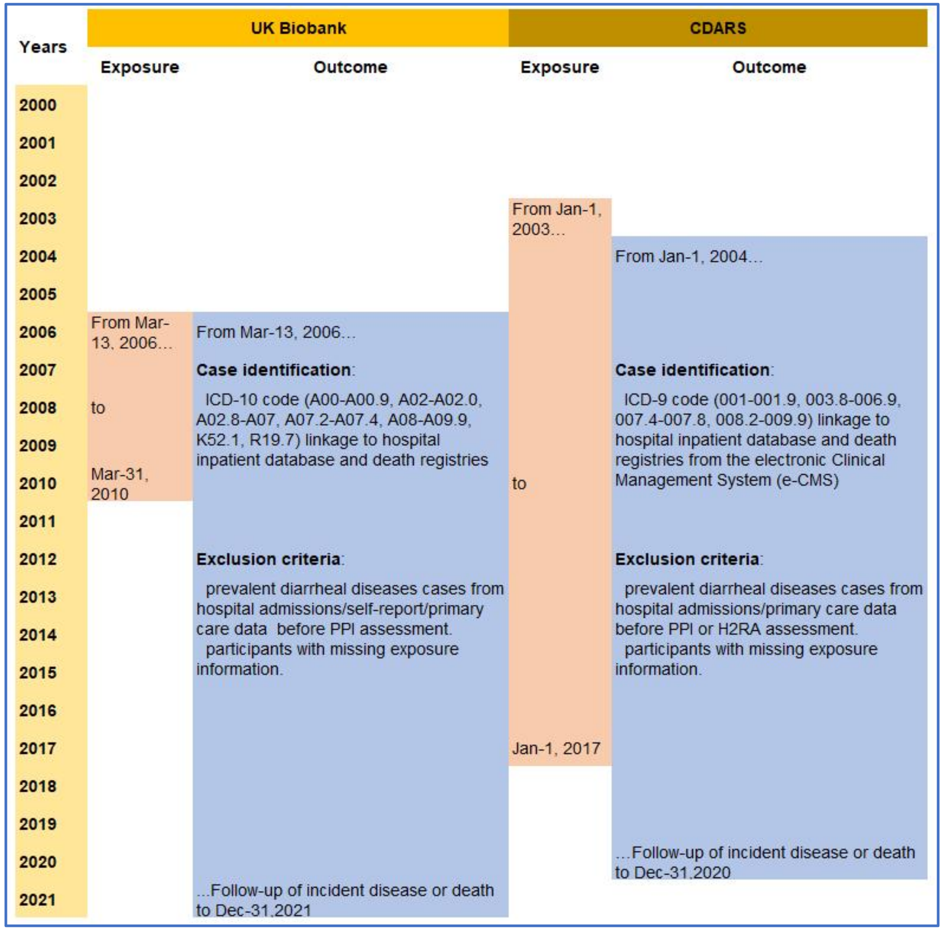

Supplementary Figure 22. Framework for diarrheal diseases in the present study.

Abbreviation: PPI, proton pump inhibitor; H2RA, H2 receptor antagonist; CDARS, clinical data analysis and reporting system; ICD, international classification of disease.

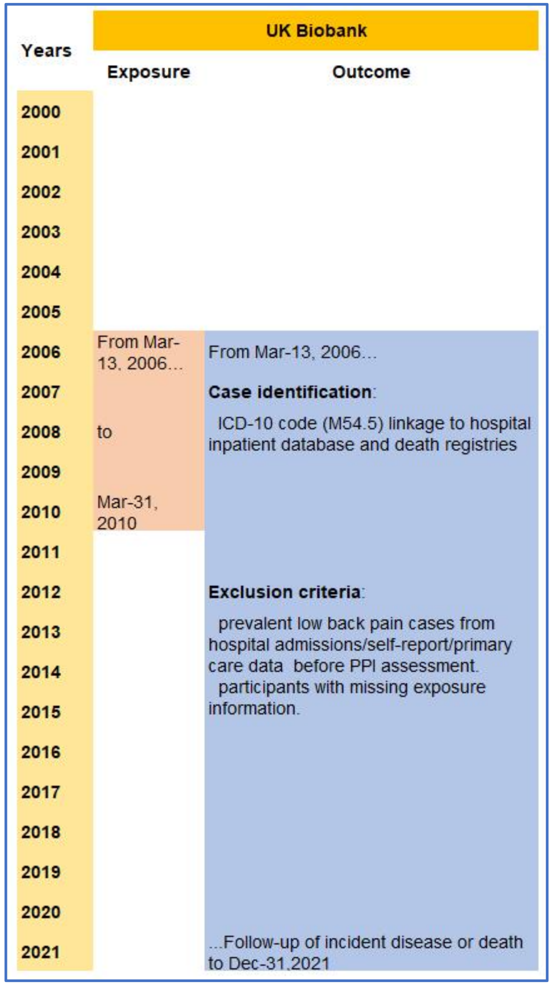

Supplementary Figure 23. Framework for low back pain in the present study.

Abbreviation: PPI, proton pump inhibitor; ICD, international classification of disease.

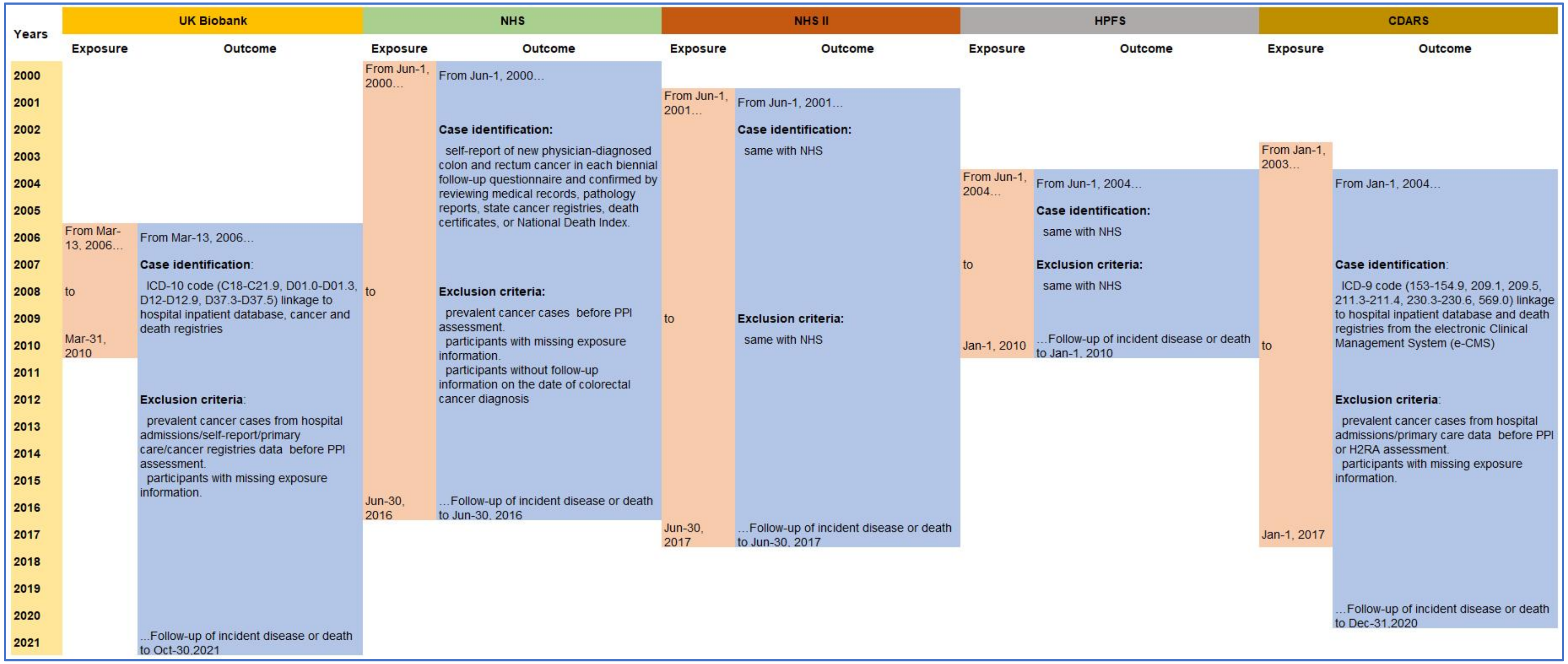

Supplementary Figure 24. Framework for colon and rectum cancer (CRC) in the present study.

Abbreviation: PPI, proton pump inhibitor; H2RA, H2 receptor antagonist; NHS, nurses' health study; HPFS, health professionals follow-up study; CDARS, clinical data analysis and reporting system; ICD, international classification of disease.

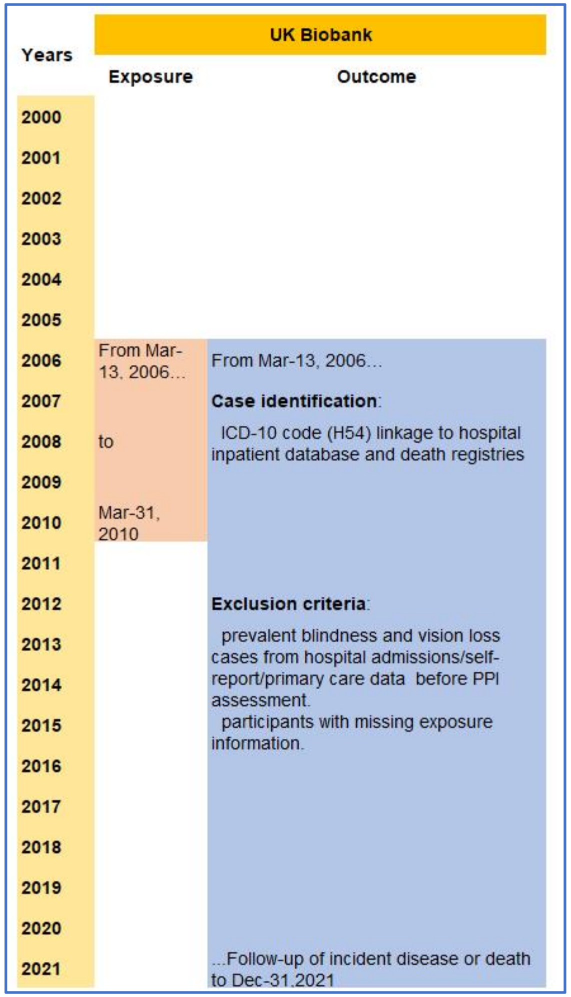

Supplementary Figure 25. Framework for blindness and vision loss in the present study.

Abbreviation: PPI, proton pump inhibitor; ICD, international classification of disease.

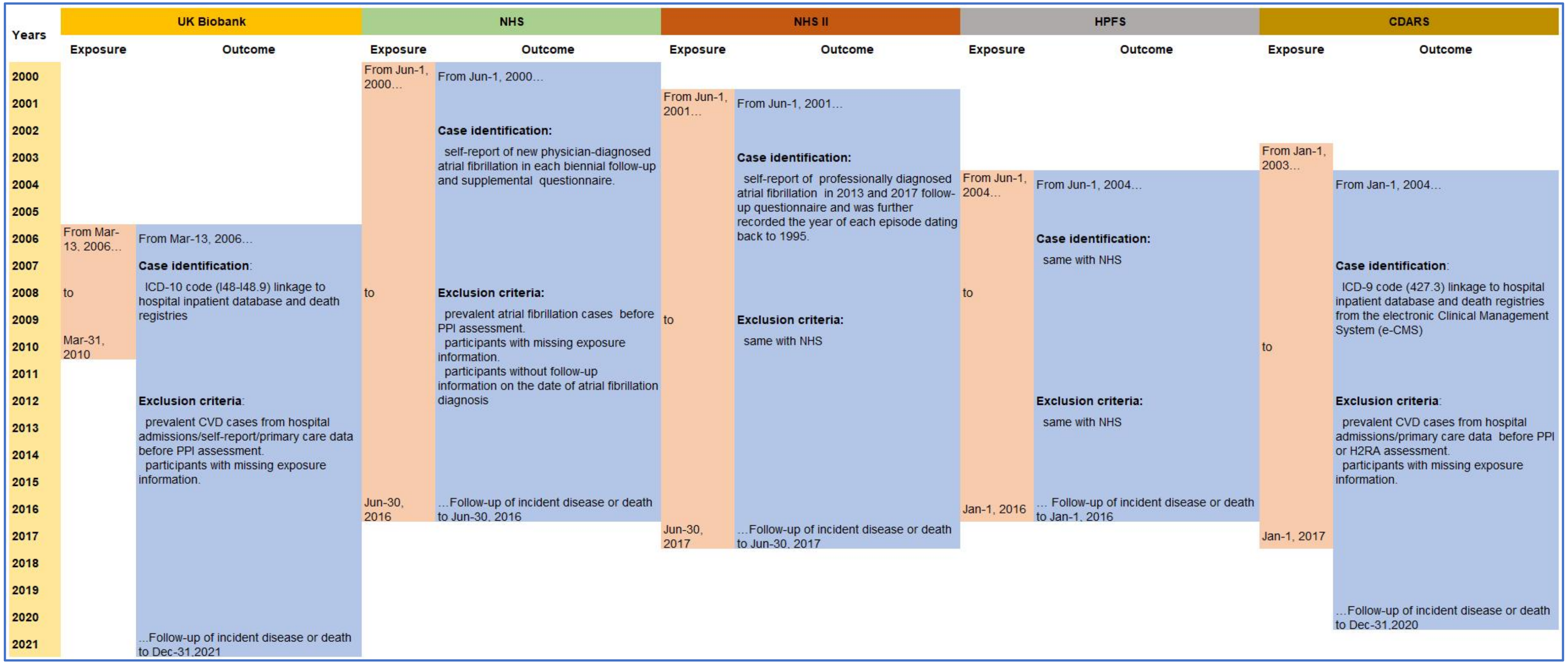

Supplementary Figure 26. Framework for atrial fibrillation and flutter in the present study.

Abbreviation: PPI, proton pump inhibitor; H2RA, H2 receptor antagonist; NHS, nurses' health study; HPFS, health professionals follow-up study; CDARS, clinical data analysis and reporting system; ICD, international classification of disease.

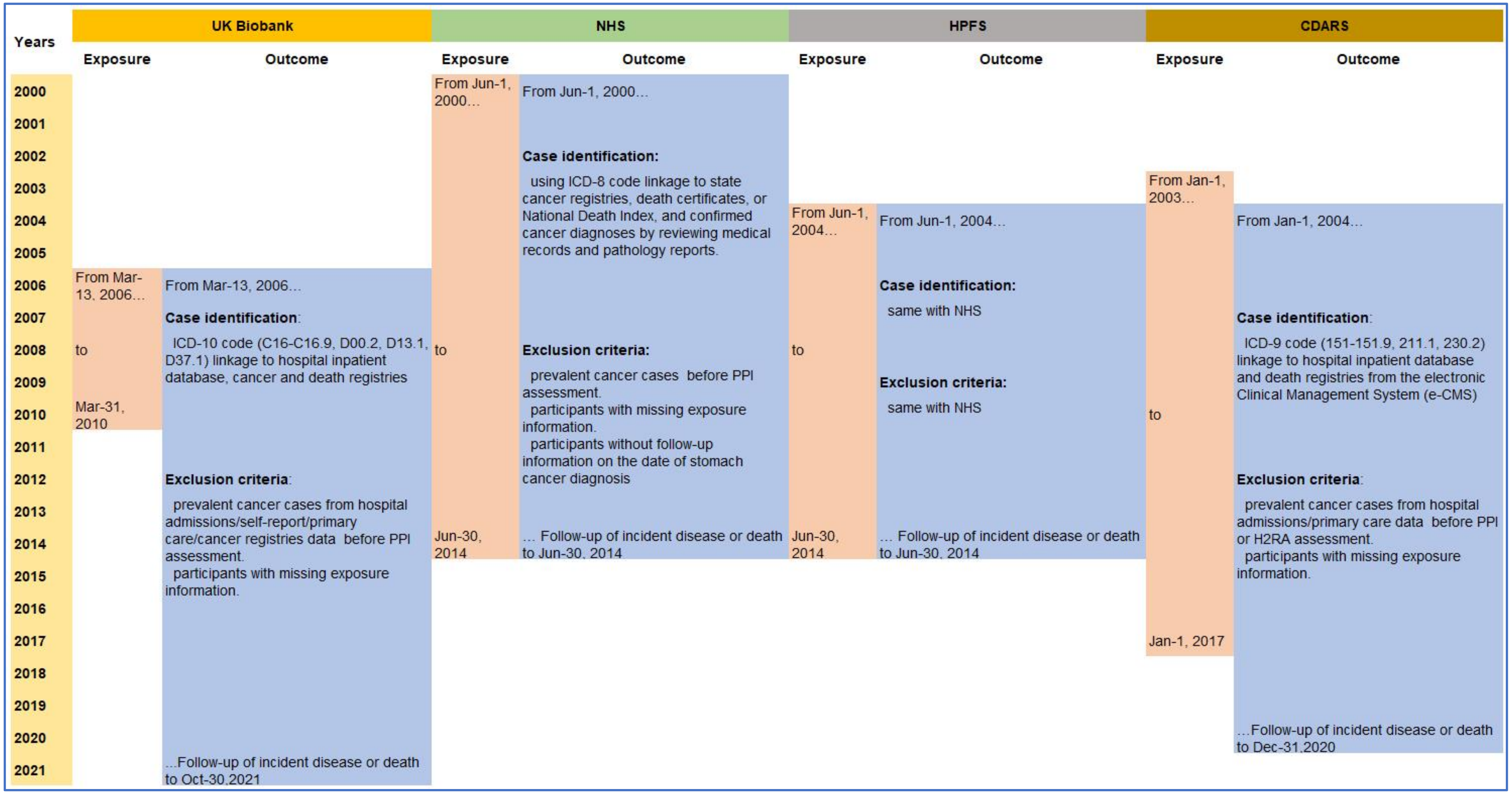

Supplementary Figure 27. Framework for stomach cancer in the present study.

Abbreviation: PPI, proton pump inhibitor; H2RA, H2 receptor antagonist; NHS, nurses' health study; HPFS, health professionals follow-up study; CDARS, clinical data analysis and reporting system; ICD, international classification of disease.

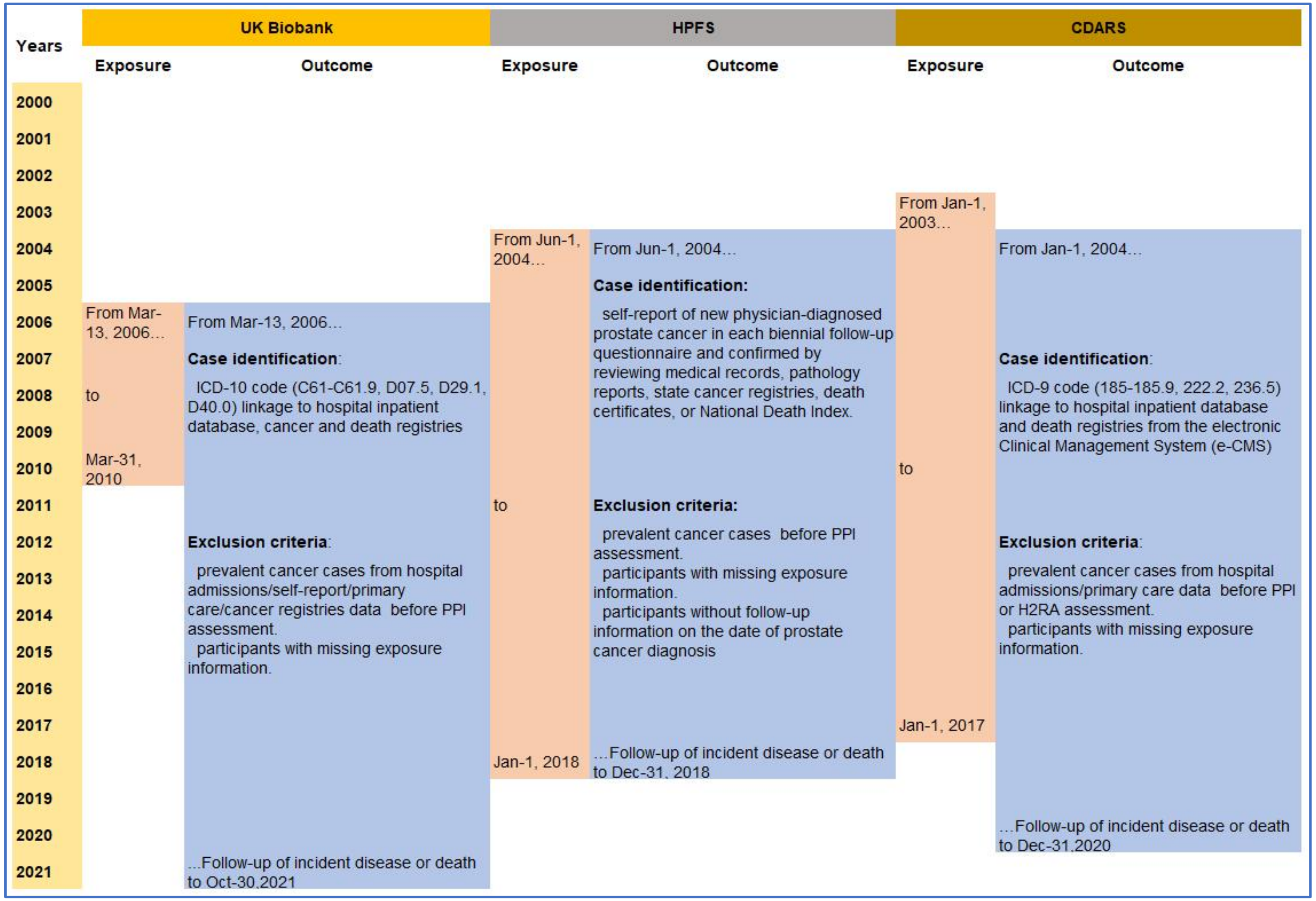

Supplementary Figure 28. Framework for prostate cancer in the present study.

Abbreviation: PPI, proton pump inhibitor; H2RA, H2 receptor antagonist; HPFS, health professionals follow-up study; CDARS, clinical data analysis and reporting system; ICD, international classification of disease.

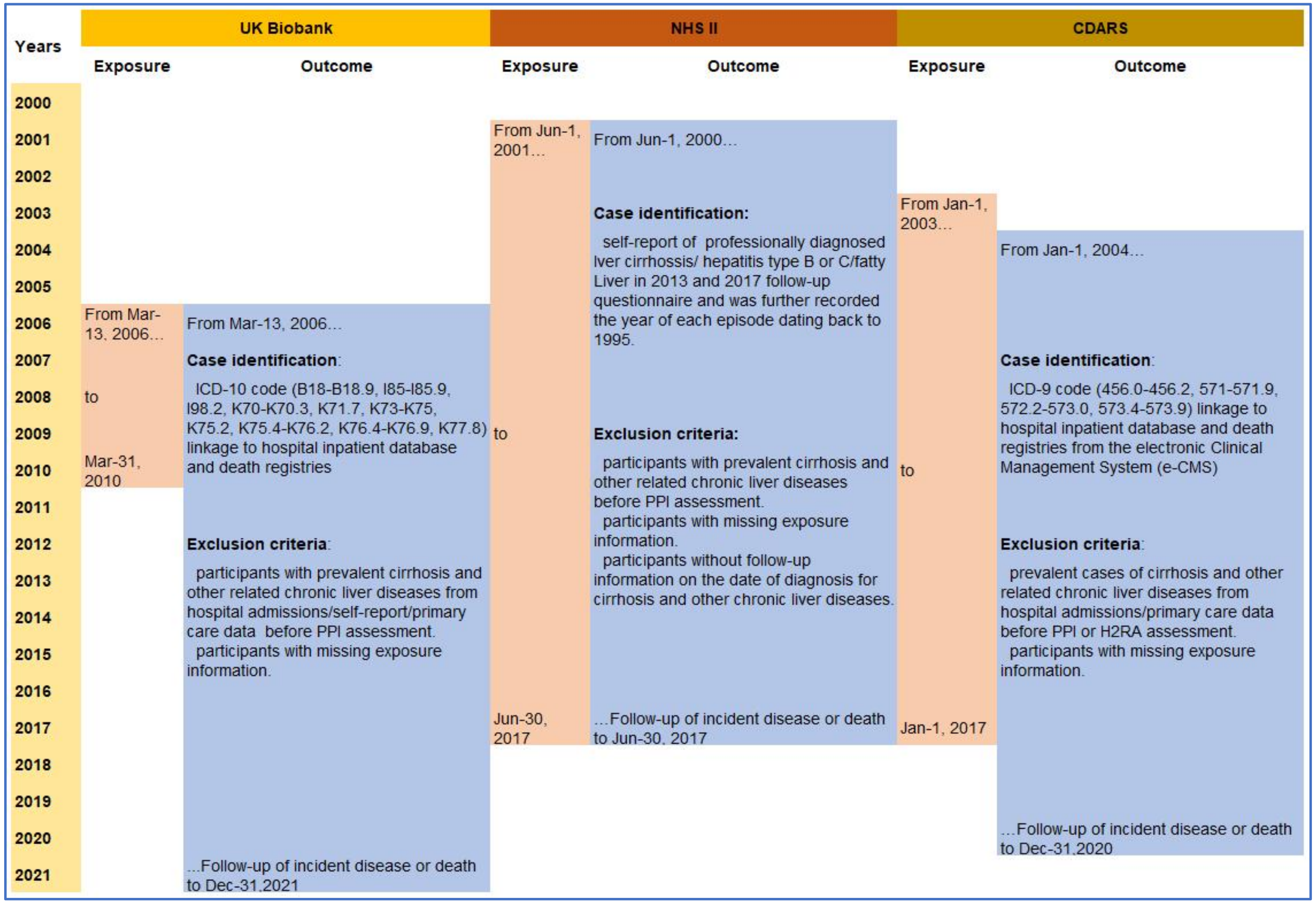

Supplementary Figure 29. Framework for cirrhosis and other chronic liver diseases in the present study.

Abbreviation: PPI, proton pump inhibitor; H2RA, H2 receptor antagonist; NHS, nurses' health study; CDARS, clinical data analysis and reporting system; ICD, international classification of disease.

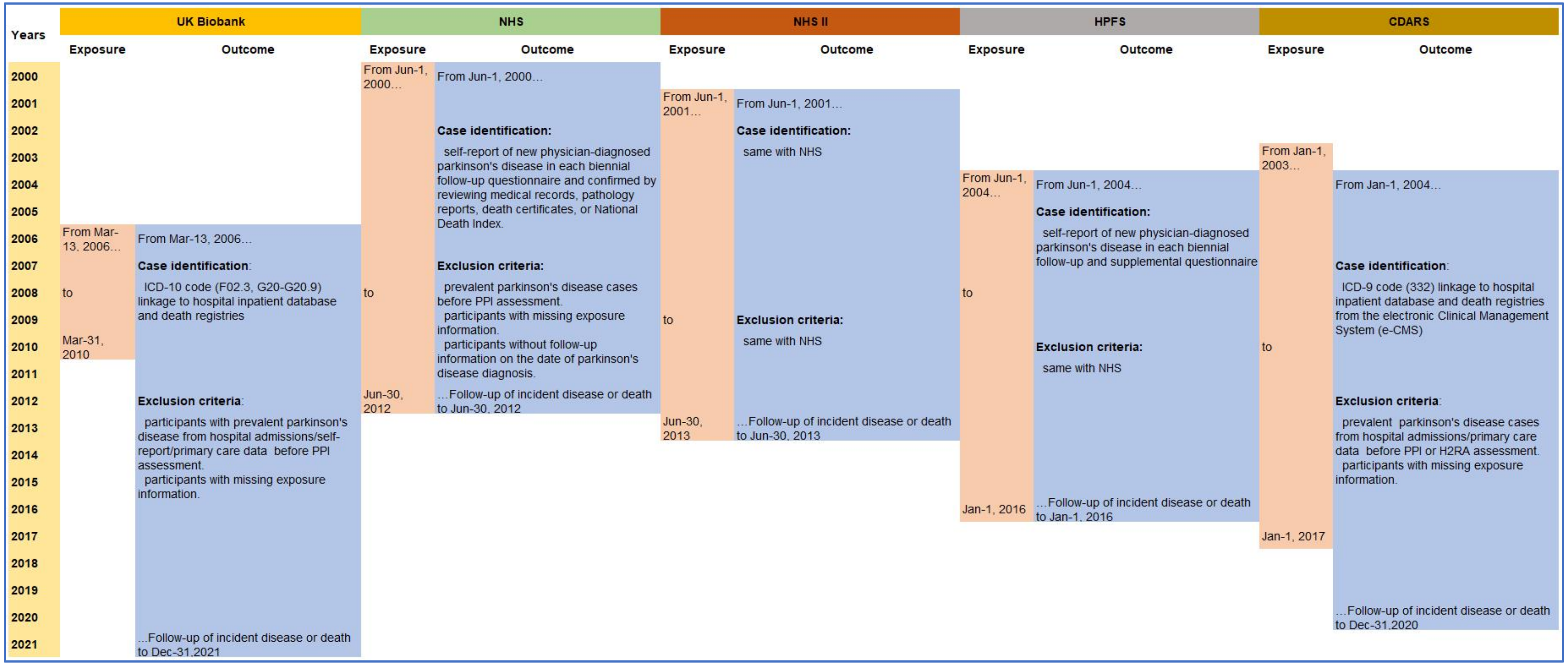

Supplementary Figure 30. Framework for Parkinson's disease in the present study.

Abbreviation: PPI, proton pump inhibitor; H2RA, H2 receptor antagonist; NHS, nurses' health study; HPFS, health professionals follow-up study; CDARS, clinical data analysis and reporting system; ICD, international classification of disease.

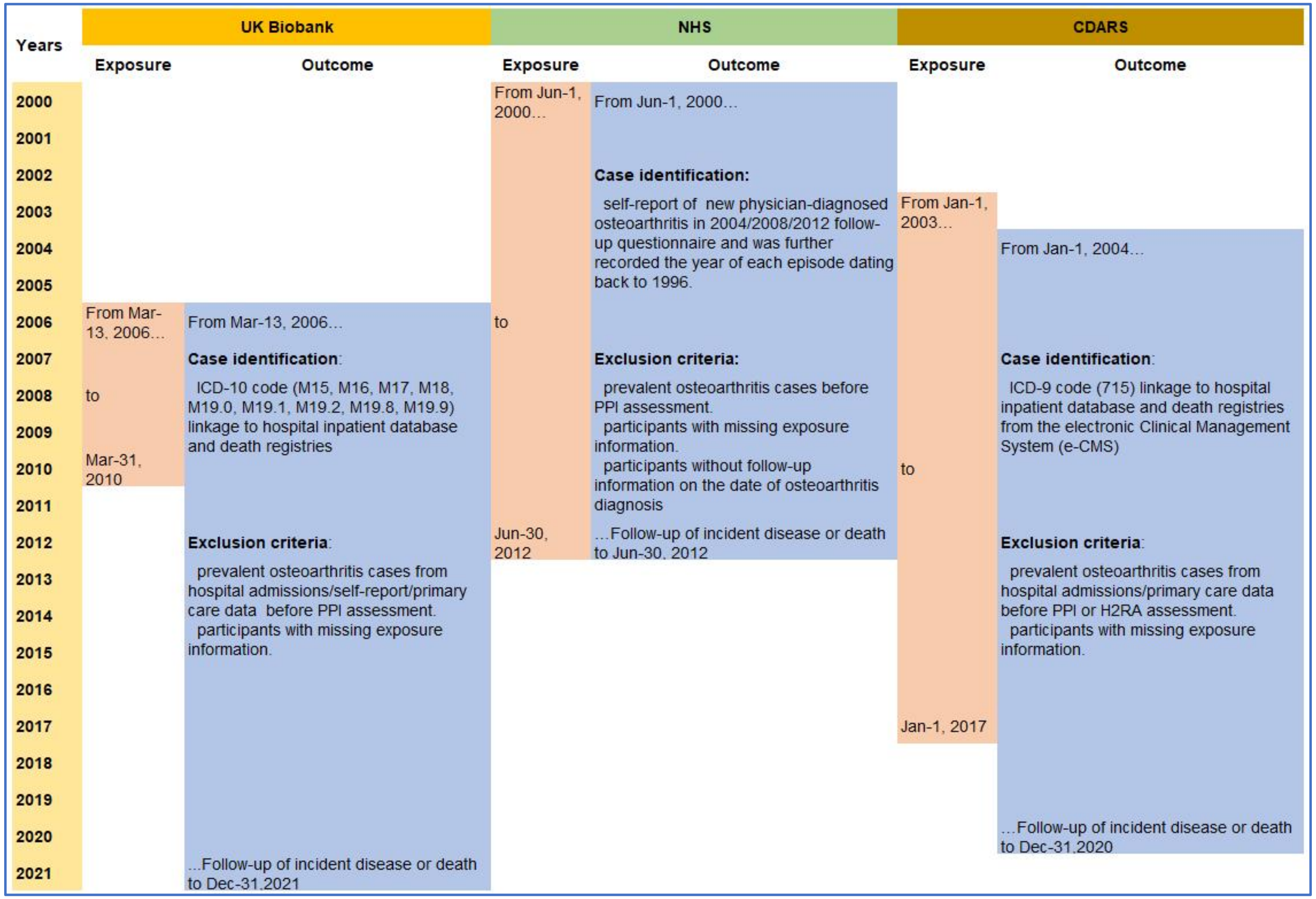

Supplementary Figure 31. Framework for osteoarthritis in the present study.

Abbreviation: PPI, proton pump inhibitor; H2RA, H2 receptor antagonist; NHS, nurses' health study; CDARS, clinical data analysis and reporting system; ICD, international classification of disease.

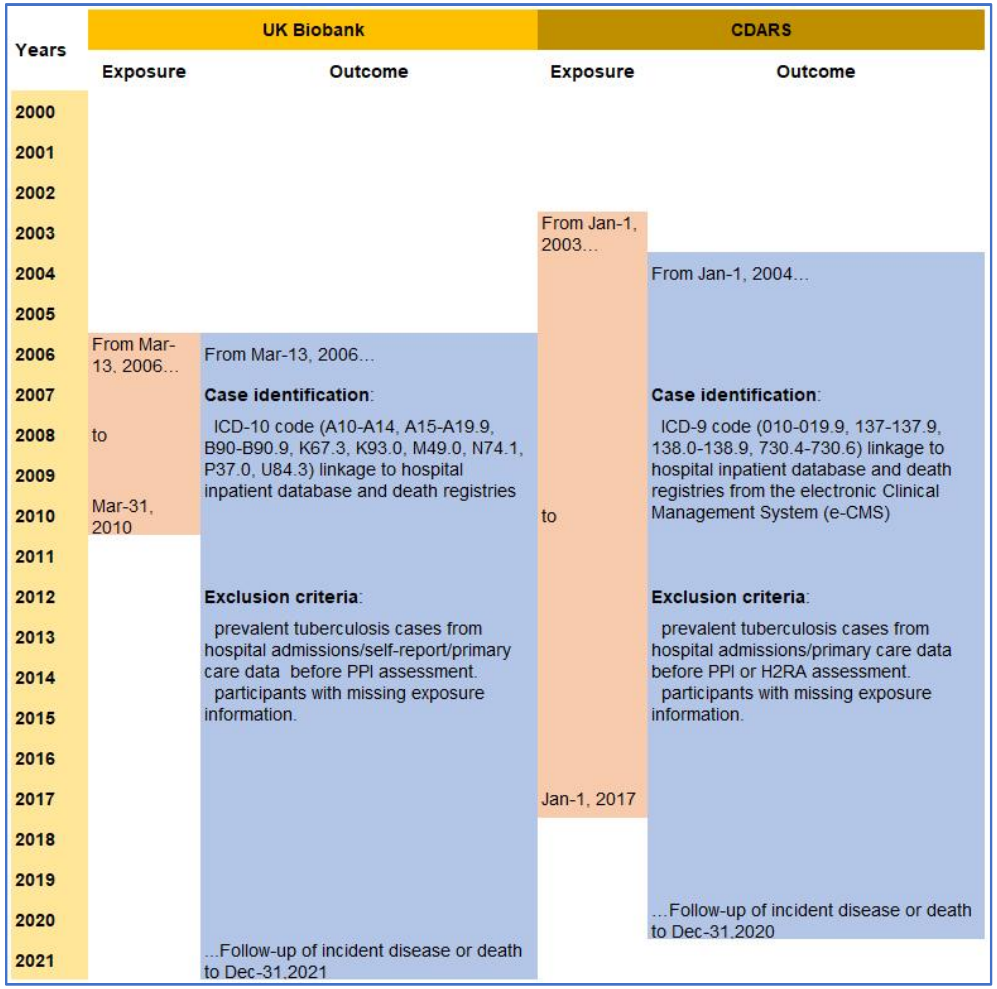

Supplementary Figure 32. Framework for tuberculosis in the present study.

Abbreviation: PPI, proton pump inhibitor; H2RA, H2 receptor antagonist; CDARS, clinical data analysis and reporting system; ICD, international classification of disease.

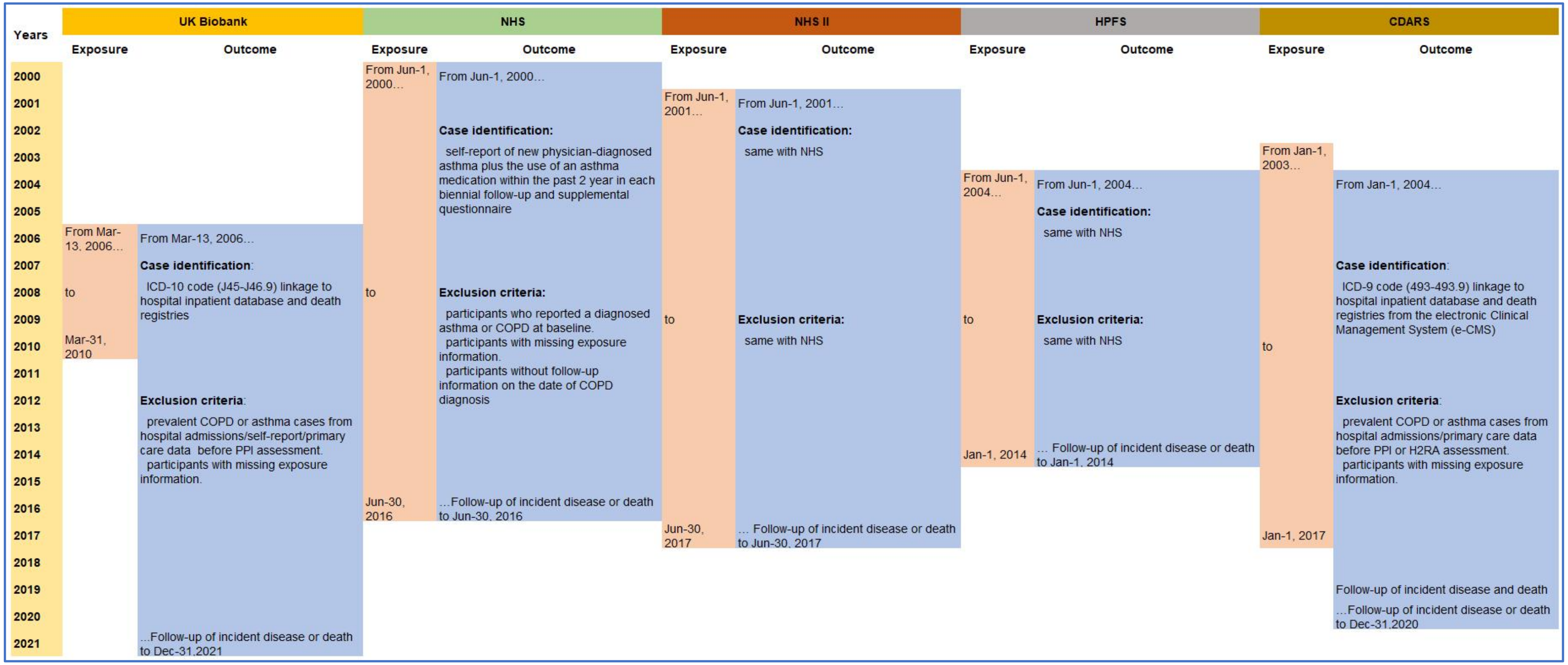

Supplementary Figure 33. Framework for asthma in the present study.

Abbreviation: PPI, proton pump inhibitor; H2RA, H2 receptor antagonist; NHS, nurses' health study; HPFS, health professionals follow-up study; CDARS, clinical data analysis and reporting system; ICD, international classification of disease.

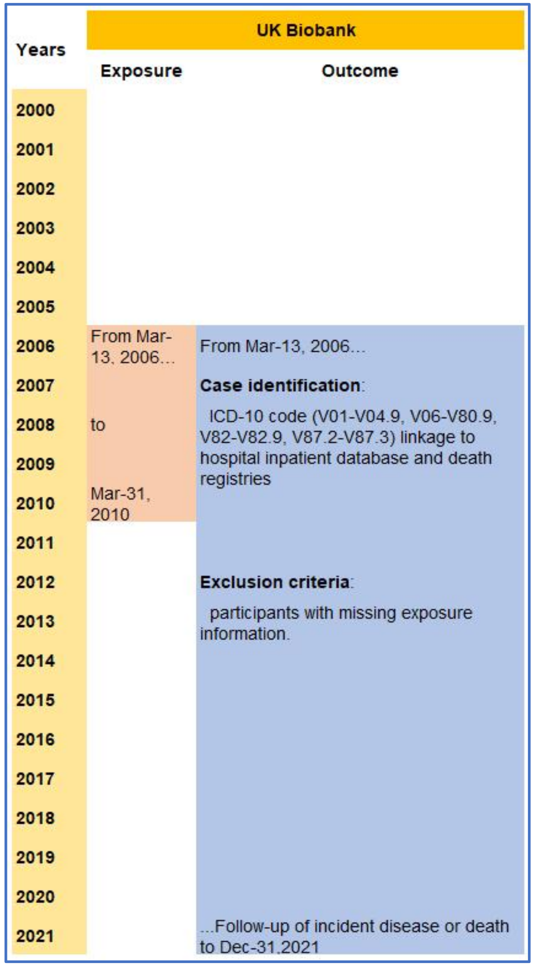

Supplementary Figure 34. Framework for road injuries in the present study.

Abbreviation: PPI, proton pump inhibitor; ICD, international classification of disease.

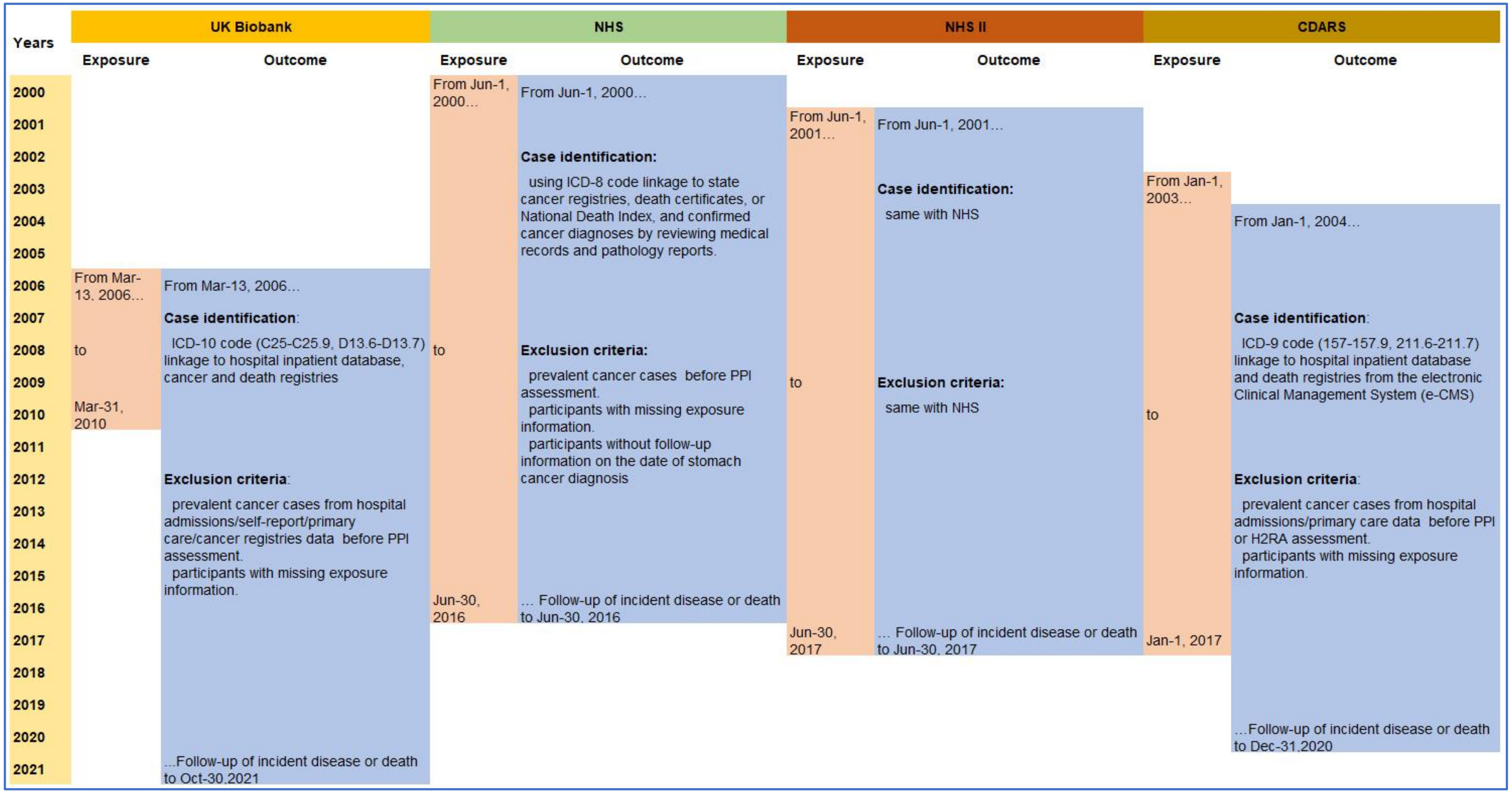

Supplementary Figure 35. Framework for pancreatic cancer in the present study.

Abbreviation: PPI, proton pump inhibitor; H2RA, H2 receptor antagonist; NHS, nurses' health study; HPFS, health professionals follow-up study; CDARS, clinical data analysis and reporting system; ICD, international classification of disease.

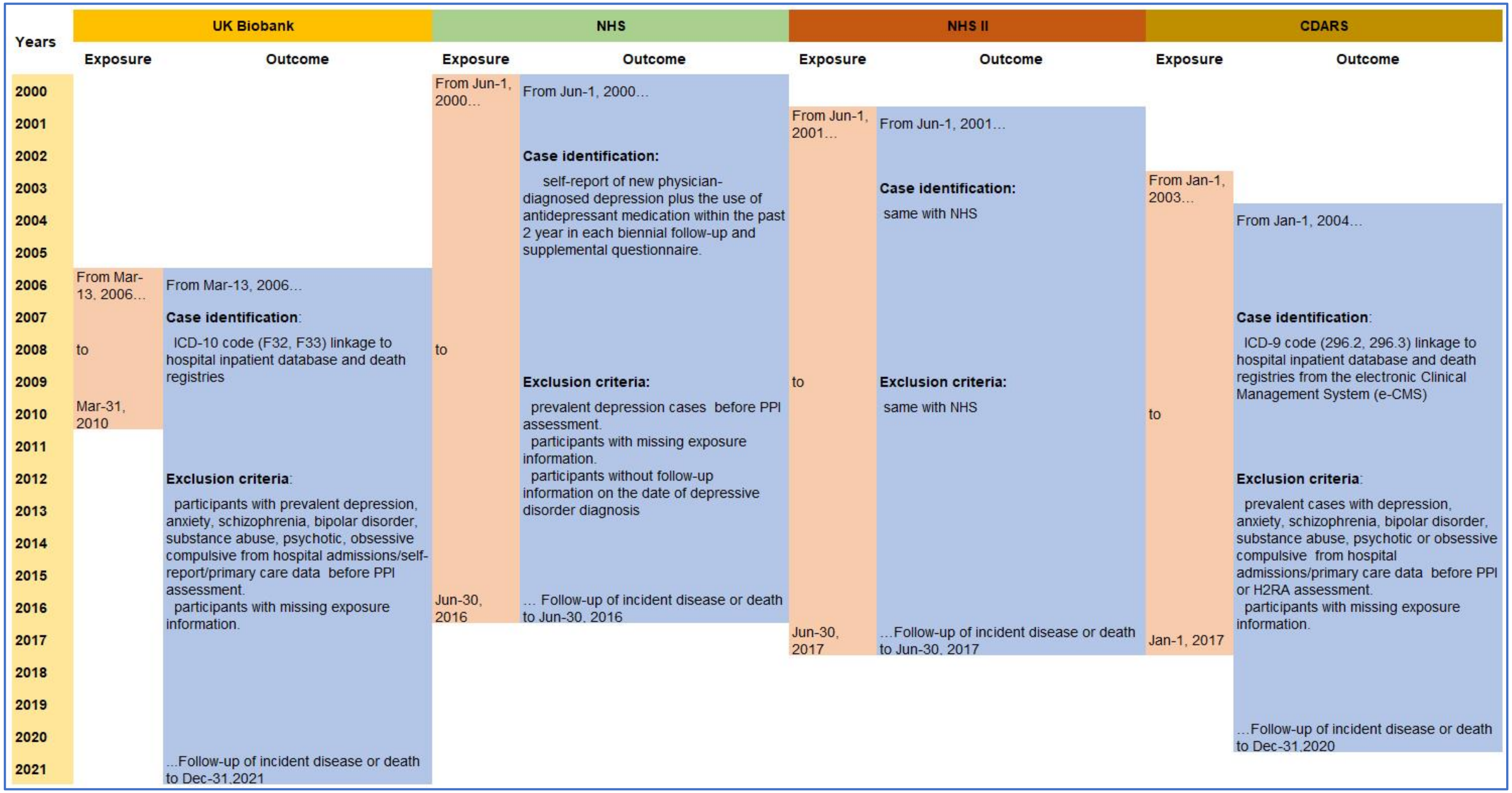

Supplementary Figure 36. Framework for depressive disorders in the present study.

Abbreviation: PPI, proton pump inhibitor; H2RA, H2 receptor antagonist; NHS, nurses' health study; HPFS, health professionals follow-up study; CDARS, clinical data analysis and reporting system; ICD, international classification of disease.

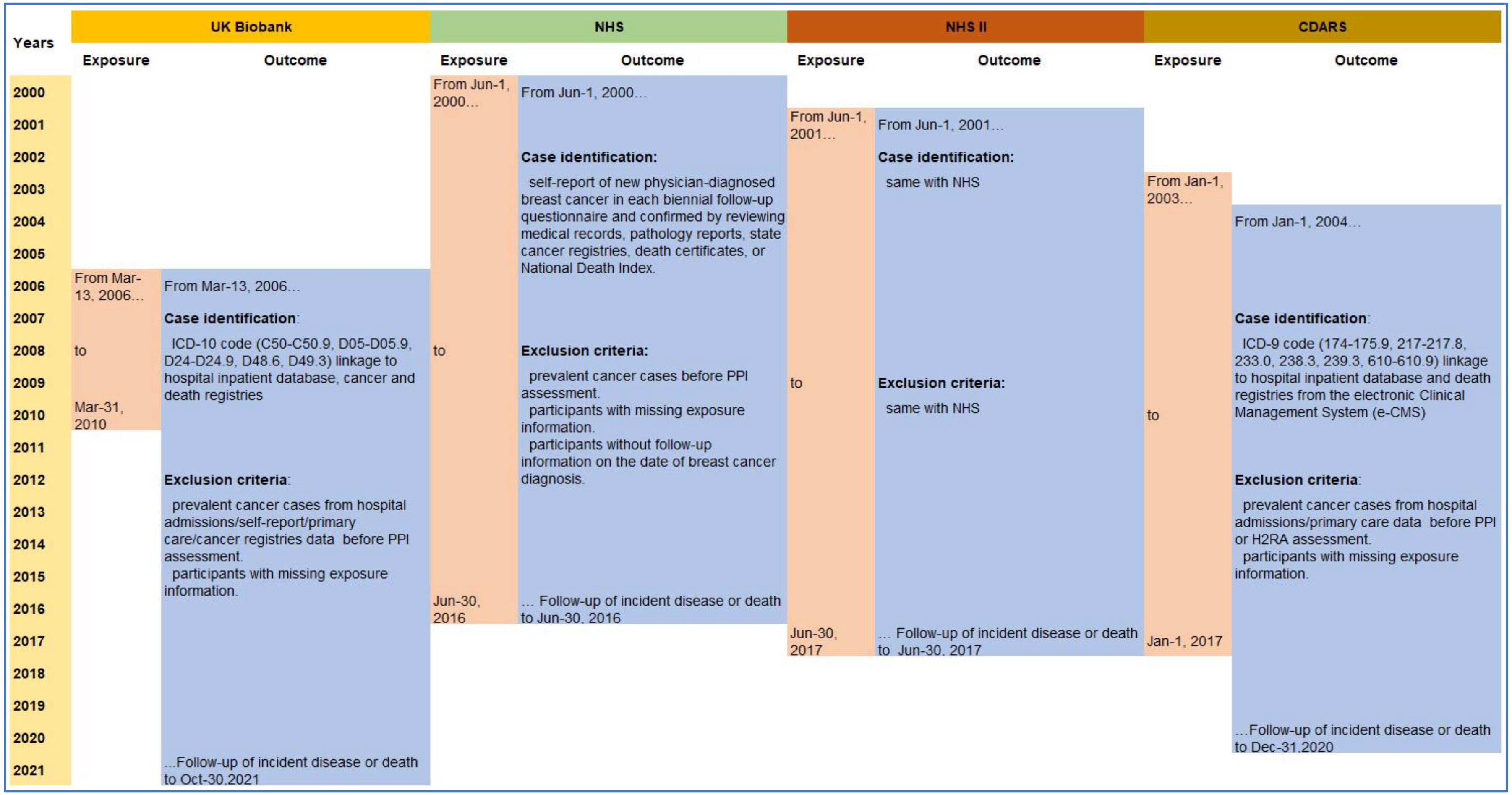

Supplementary Figure 37. Framework for breast cancer in the present study.

Abbreviation: PPI, proton pump inhibitor; H2RA, H2 receptor antagonist; NHS, nurses' health study; HPFS, health professionals follow-up study; CDARS, clinical data analysis and reporting system; ICD, international classification of disease.

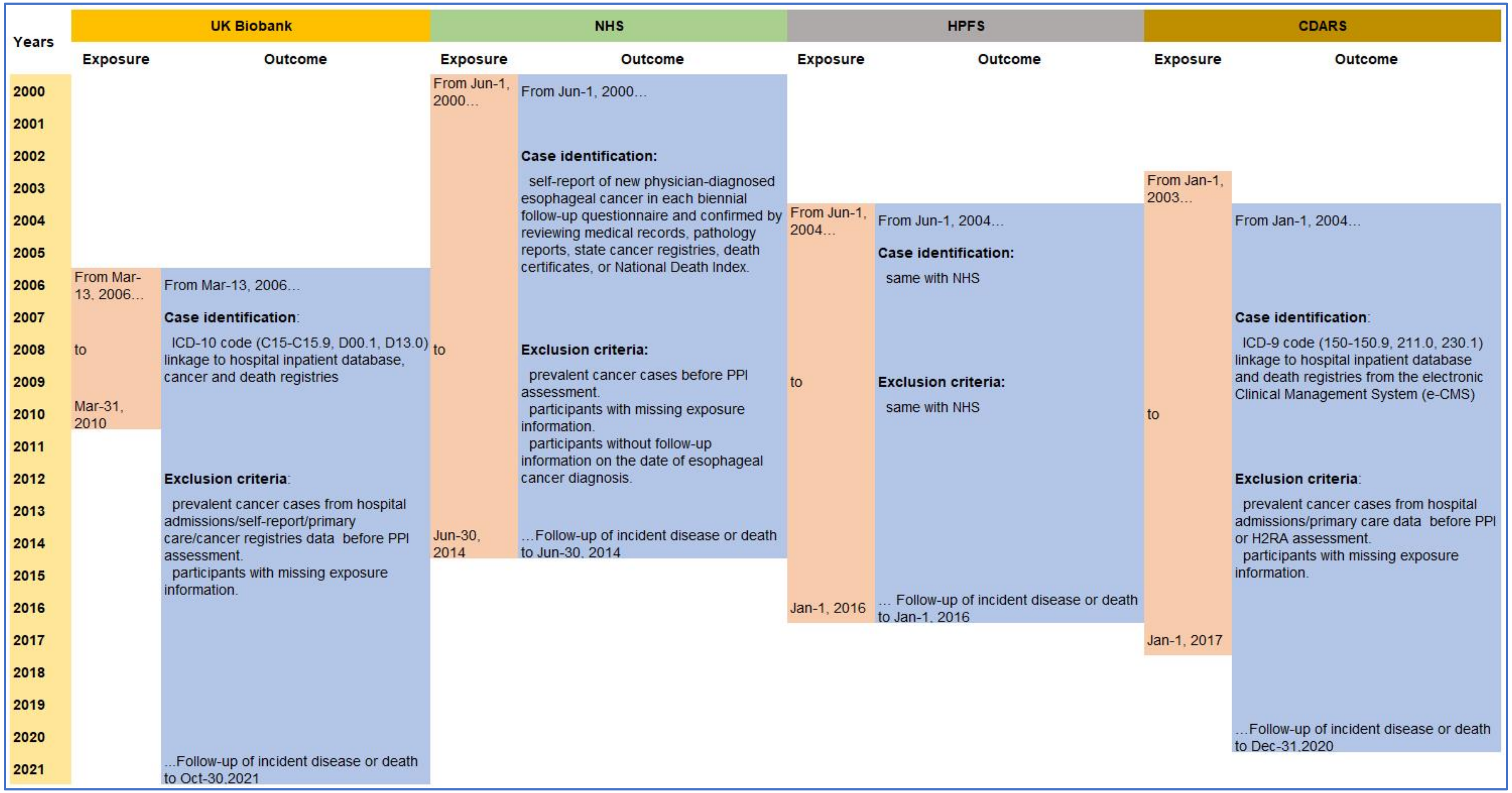

Supplementary Figure 38. Framework for esophageal cancer in the present study.

Abbreviation: PPI, proton pump inhibitor; H2RA, H2 receptor antagonist; NHS, nurses' health study; HPFS, health professionals follow-up study; CDARS, clinical data analysis and reporting system; ICD, international classification of disease.

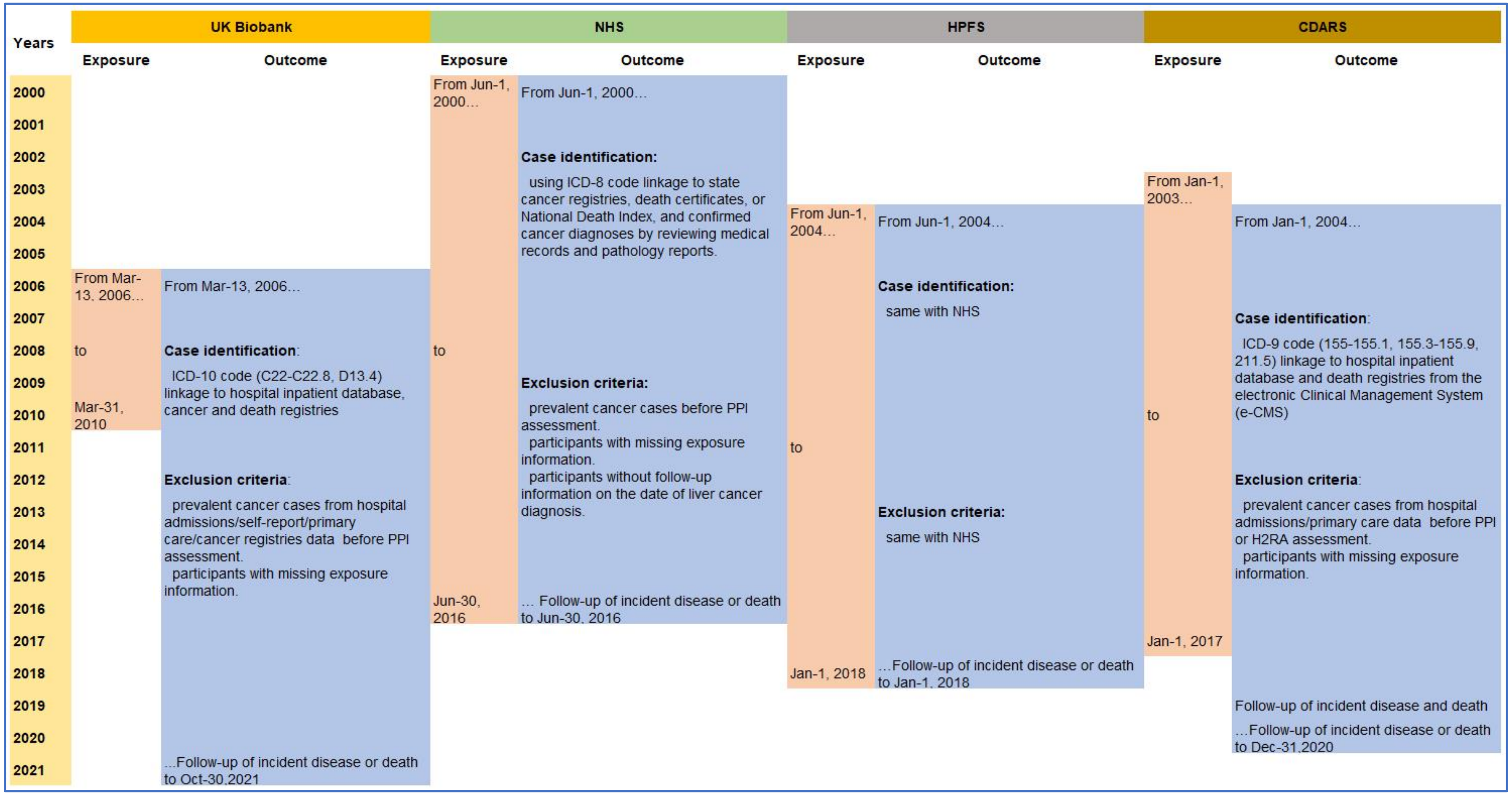

Supplementary Figure 39. Framework for liver cancer in the present study.

Abbreviation: PPI, proton pump inhibitor; H2RA, H2 receptor antagonist; NHS, nurses' health study; HPFS, health professionals follow-up study; CDARS, clinical data analysis and reporting system; ICD, international classification of disease.

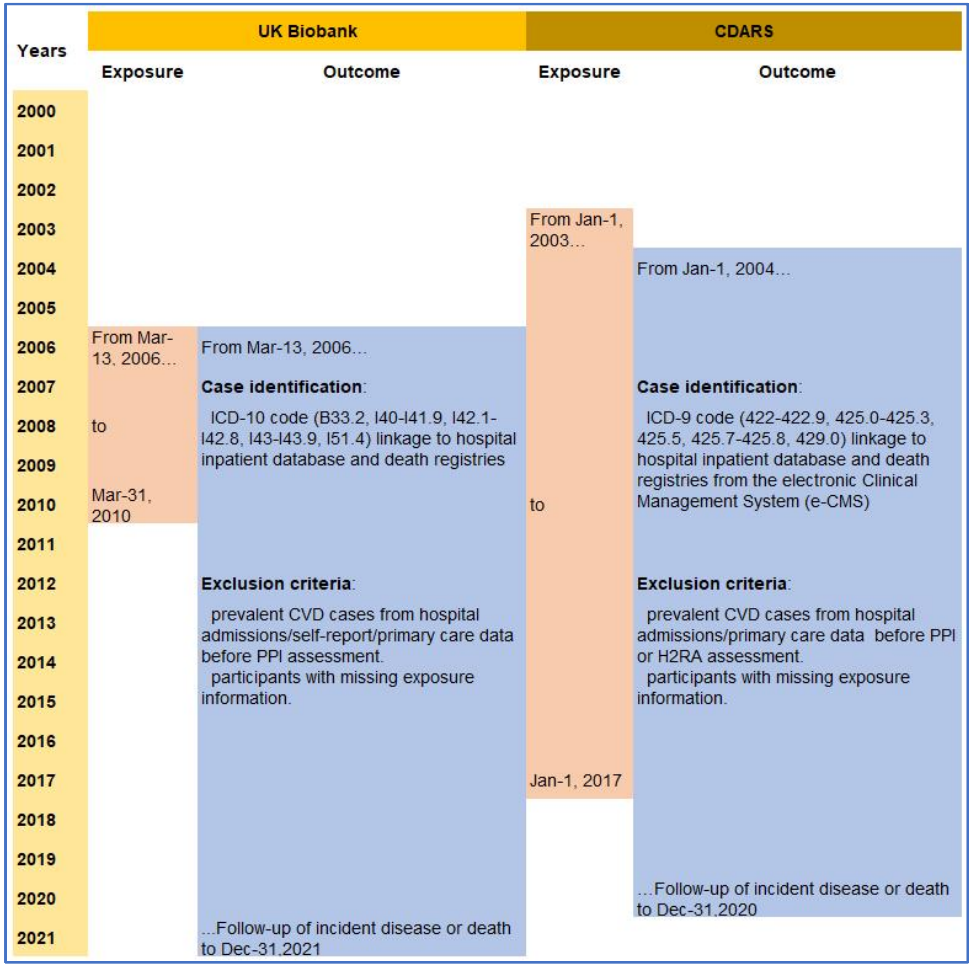

Supplementary Figure 40. Framework for cardiomyopathy and myocarditis in the present study.

Abbreviation: PPI, proton pump inhibitor; H2RA, H2 receptor antagonist; CDARS, clinical data analysis and reporting system; ICD, international classification of disease.

STROBE Statement—Checklist of items that should be included in reports of *cohort studies*

	Item No	Recommendation	page
**Title and abstract**	1	(*a*) Indicate the study's design with a commonly used term in the title or the abstract	1
		(*b*) Provide in the abstract an informative and balanced summary of what was done and what was found	3
**Introduction**			
Background/rationale	2	Explain the scientific background and rationale for the investigation being reported	4
Objectives	3	State specific objectives, including any prespecified hypotheses	4
**Methods**			
Study design	4	Present key elements of study design early in the paper	12 and supplementary file page 68
Setting	5	Describe the setting, locations, and relevant dates, including periods of recruitment, exposure, follow-up, and data collection	12 and supplementary file page 69-98
Participants	6	(*a*) Give the eligibility criteria, and the sources and methods of selection of participants. Describe methods of follow-up	12-14
		(*b*) For matched studies, give matching criteria and number of exposed and unexposed	12-13
Variables	7	Clearly define all outcomes, exposures, predictors, potential confounders, and effect modifiers. Give diagnostic criteria, if applicable	13-14
Data sources/ measurement	8*	For each variable of interest, give sources of data and details of methods of assessment (measurement). Describe comparability of assessment methods if there is more than one group	12-13
Bias	9	Describe any efforts to address potential sources of bias	15-16
Study size	10	Explain how the study size was arrived at	12-13
Quantitative variables	11	Explain how quantitative variables were handled in the analyses. If applicable, describe which groupings were chosen and why	15-17
Statistical methods	12	(*a*) Describe all statistical methods, including those used to control for confounding	15-17
		(*b*) Describe any methods used to examine subgroups and interactions	15
		(*c*) Explain how missing data were addressed	15-16
		(*d*) If applicable, explain how loss to follow-up was addressed	14-15
		(*e*) Describe any sensitivity analyses	15-17
**Results**			
Participants	13*	(a) Report numbers of individuals at each stage of study—eg	4-5

		numbers potentially eligible, examined for eligibility, confirmed eligible, included in the study, completing follow-up, and analysed	
		(b) Give reasons for non-participation at each stage	supplementary file page 69-98
		(c) Consider use of a flow diagram	supplementary file page 69-98
Descriptive data	14*	(a) Give characteristics of study participants (eg demographic, clinical, social) and information on exposures and potential confounders	5 and 25
		(b) Indicate number of participants with missing data for each variable of interest	5
		(c) Summarise follow-up time (eg, average and total amount)	supplementary file page 69-98
Outcome data	15*	Report numbers of outcome events or summary measures over time	5 and supplementary file page 10-39
Main results	16	(*a*) Give unadjusted estimates and, if applicable, confounder-adjusted estimates and their precision (eg, 95% confidence interval). Make clear which confounders were adjusted for and why they were included	5 and supplementary file page 10-39
		(*b*) Report category boundaries when continuous variables were categorized	5 and 26
		(*c*) If relevant, consider translating estimates of relative risk into absolute risk for a meaningful time period	5-6
Other analyses	17	Report other analyses done—eg analyses of subgroups and interactions, and sensitivity analyses	5-6 and supplementary file page 59-67
**Discussion**			
Key results	18	Summarise key results with reference to study objectives	6-7
Limitations	19	Discuss limitations of the study, taking into account sources of potential bias or imprecision. Discuss both direction and magnitude of any potential bias	9
Interpretation	20	Give a cautious overall interpretation of results considering objectives, limitations, multiplicity of analyses, results from similar studies, and other relevant evidence	6-10
Generalisability	21	Discuss the generalisability (external validity) of the study results	10
**Other information**			
Funding	22	Give the source of funding and the role of the funders for the present study and, if applicable, for the original study on which the present article is based	11

*Give information separately for exposed and unexposed groups.

Note: An Explanation and Elaboration article discusses each checklist item and gives methodological background and published examples of transparent reporting. The STROBE checklist is best used in conjunction with this article (freely available on the Web sites of PLoS Medicine at http://www.plosmedicine.org/, Annals of Internal Medicine at http://www.annals.org/, and Epidemiology at http://www.epidem.com/). Information on the STROBE Initiative is available at http://www.strobe-statement.org.
