# Supplementary material for: Individualized prevention of proton pump inhibitor related adverse events by risk stratification
Source: Nat Commun. 2024 Apr 27;15:3591. doi: 10.1038/s41467-024-48007-8 (PMC11055952; doi:10.1038/s41467-024-48007-8)
Supplement: Supplementary file 3 — Description of Additional Supplementary Files [file 41467_2024_48007_MOESM3_ESM.pdf]

Description of Additional Supplementary Files

Supplementary Data 1. Risk prediction models for the PPI-associated outcomes
This data presents literatures for appropriate prediction models and the prediction models for evaluating baseline risk.

Supplementary Data 2. Comparison the findings of the current study and the latest systematic reviews/original studies.
This data showed the most updated meta-analyses and recent original studies that were systematically searched using PubMed, EMBASE, and Web of Science from the inception to 1st December 2022. As shown, our results are consistent with previous meta-analyses/original studies which showed a positive association between PPI and stroke, lower respiratory infections, falls, CKD, diarrheal diseases, asthma, pancreatic cancer, Parkinson's disease, and depressive disorders.
