# Supplementary material for: Individualized prevention of proton pump inhibitor related adverse events by risk stratification
Source: Nat Commun. 2024 Apr 27;15:3591. doi: 10.1038/s41467-024-48007-8 (PMC11055952; doi:10.1038/s41467-024-48007-8)
Supplement: Supplementary file 6 — Reporting Summary [file 41467_2024_48007_MOESM6_ESM.pdf]

nature portfolio

Corresponding author(s): Jinqiu Yuan

Last updated by author(s): 16/03/24

Reporting Summary

Nature Portfolio wishes to improve the reproducibility of the work that we publish. This form provides structure for consistency and transparency in reporting. For further information on Nature Portfolio policies, see our Editorial Policies and the Editorial Policy Checklist.

Statistics

For all statistical analyses, confirm that the following items are present in the figure legend, table legend, main text, or Methods section.

n/a	Confirmed	
☐	☒	The exact sample size (*n*) for each experimental group/condition, given as a discrete number and unit of measurement
☐	☒	A statement on whether measurements were taken from distinct samples or whether the same sample was measured repeatedly
☐	☒	The statistical test(s) used AND whether they are one- or two-sided<br>*Only common tests should be described solely by name; describe more complex techniques in the Methods section.*
☐	☒	A description of all covariates tested
☐	☒	A description of any assumptions or corrections, such as tests of normality and adjustment for multiple comparisons
☐	☒	A full description of the statistical parameters including central tendency (e.g. means) or other basic estimates (e.g. regression coefficient) AND variation (e.g. standard deviation) or associated estimates of uncertainty (e.g. confidence intervals)
☐	☒	For null hypothesis testing, the test statistic (e.g. *F*, *t*, *r*) with confidence intervals, effect sizes, degrees of freedom and *P* value noted<br>*Give P values as exact values whenever suitable.*
☒	☐	For Bayesian analysis, information on the choice of priors and Markov chain Monte Carlo settings
☐	☒	For hierarchical and complex designs, identification of the appropriate level for tests and full reporting of outcomes
☒	☐	Estimates of effect sizes (e.g. Cohen's *d*, Pearson's *r*), indicating how they were calculated

Our web collection on statistics for biologists contains articles on many of the points above.

Software and code

Policy information about availability of computer code

Data collection	A total of 2 079 724 participants from UK Biobank (n=501 109), Nurses' Health Study (NHS, n=91 708), NHS II (n=99 641), Health Professionals Follow-Up Study (HPFS, n=30 933), and Clinical Data Analysis and Reporting System (CDARS, n=1 356 333) were included as the basic population for the current analyses. The NHS, NHS II and HPFS resource were approved by the Human Research Committee at the Brigham and Women's Hospital, Boston, MA. This study got access to the UK Biobank under application number 51671. The CDRAS database was approved by the Institutional Review Board of the University of Hong Kong and the West Cluster of the Hong Kong Hospital Authority.
Data analysis	All analyses were performed using the SAS software, Version 9.4 (SAS Institute, Cary, North Carolina, USA) and R software (R Foundation for Statistical Computing, Vienna, Austria, version 3.5.0)

For manuscripts utilizing custom algorithms or software that are central to the research but not yet described in published literature, software must be made available to editors and reviewers. We strongly encourage code deposition in a community repository (e.g. GitHub). See the Nature Portfolio guidelines for submitting code & software for further information.

Data

Policy information about availability of data

All manuscripts must include a data availability statement. This statement should provide the following information, where applicable:

- Accession codes, unique identifiers, or web links for publicly available datasets
- A description of any restrictions on data availability
- For clinical datasets or third party data, please ensure that the statement adheres to our policy

The UK Biobank data used in this study was available from UK Biobank under application number 51671. The CDARS database is under restricted access to researchers upon approval by the Institutional Review Board of the University of Hong Kong and the West Cluster of the Hong Kong Hospital Authority . NHS, NHS II and HPFS are available upon formal application to and approval by the Channing Division of Network Medicine at Brigham and Women's Hospital, and Harvard T.H. Chan School of Public Health. For the protection of confidentiality and privacy of cohort participants, written request for access to the data is required. The standard procedure for controlled access requires that applications to use the resources of the Nurses' Health Studies and Health Professionals Follow-up Study undergo a formal review by the cohort committee. The committee assesses the scientific aims, examines the suitability of the proposed methodology for the available data, and confirms that the proposed use aligns with the guidelines of the Ethics and Governance Framework. Further information including the procedures to obtain and access data from the NHS, NHS II and HPFS is described at https://www.nurseshealthstudy.org/researchers (email: nhsaccess@channing.harvard.edu) and https://sites.sph.harvard.edu/hpfs/for-collaborators/ .

Research involving human participants, their data, or biological material

Policy information about studies with human participants or human data. See also policy information about sex, gender (identity/presentation), and sexual orientation and race, ethnicity and racism.

Reporting on sex and gender	Throughout the entire manuscript, we use the term "sex" to denote biological differences. In the study design, we incorporated cohorts such as the NHS and NHS cohorts, consisting exclusively of females, and the HPFS dataset, comprising only males. Additionally, we included datasets like the UK Biobank and CDARS, which cover both sexes, allowing our findings to be extrapolated to both genders. The definition of "sex" in our study is based on self-reported information. We did not collect any information on "gender" in the various cohorts. In our research, we conducted subgroup analyses based on "sex," and the results consistently showed that our main findings hold true across different gender populations.
Reporting on race, ethnicity, or other socially relevant groupings	In our study, we collected self-reported racial information from participants in various cohorts and transformed this variable into a binary classification (white or other). During data analysis, we utilized it as a covariate, correcting for its influence in Cox regression models. This approach allowed us to account for potential racial disparities and enhance the robustness of our findings by considering race as a covariate in the statistical models.
Population characteristics	The characteristics of included participants by cohort were summarized in Table 1 of the manuscript. Overall, the participants had a mean age between 48.4 and 71.4 at baseline, and with a PPI usage rate from 5 to 15%. Compared with non-PPI users, regular PPI users were more likely to be older, obese, smoking, less physically active, with higher rates of comorbidities and medication usage
Recruitment	This is a prospective analysis of five population-based prospective cohort studies including the UK Biobank (UK), NHS (U.S.), the NHS II (U.S.), HPFS (U.S.), and CDARS (China). In the current analysis, we included participants who reported information about PPI usage and excluded those with outcomes of interest prior to baseline.<br>However, it is important to acknowledge certain sources of selection bias in this study. For instance, the NHS, NHS II, and HPFS cohorts exclusively include health professionals, potentially introducing a "healthy volunteer" effect. Additionally, a similar effect may prevail within the UK Biobank, impacting the generalizability of the findings to the broader adult population.
Ethics oversight	The UK Biobank was approved by the National Research Ethics Committee (REC ID: 16/NW/0274).The NHS, NHS II were approved by the Human Research Committee at the Brigham and Women's Hospital, and the HPFS was approved by the Human Subjects Committee by the Harvard T H Chan School of Public Health, Boston, Massachusetts, USA. The CDARS database was approved by the Institutional Review Board of the University of Hong Kong and the West Cluster of the Hong Kong Hospital Authority.

Note that full information on the approval of the study protocol must also be provided in the manuscript.

Field-specific reporting

Please select the one below that is the best fit for your research. If you are not sure, read the appropriate sections before making your selection.

☒ Life sciences ☐ Behavioural & social sciences ☐ Ecological, evolutionary & environmental sciences

For a reference copy of the document with all sections, see nature.com/documents/nr-reporting-summary-flat.pdf

Life sciences study design

All studies must disclose on these points even when the disclosure is negative.

Sample size	This analysis is derived from five pre-established large national population-based cohort studies. The sample size was determined based on

Sample size	the data availability from these major population cohorts, rendering it exceptionally large. Due to the extensive nature of these cohorts, there was no need for a separate sample-size calculation. The study leveraged existing data from these substantial cohorts, ensuring robust statistical power and representativeness.
Data exclusions	In the current analysis, we pre-established criteria for the inclusion and exclusion of participants. We included individuals who reported information about our exposure of interest (PPI usage) and excluded those with outcomes of interest prior to baseline. In the CDARS database, to eliminate the residual effects of previously used PPIs, we also excluded participants who had used any PPIs two years before cohort entry.
Replication	Our study is based on data from five publicly available large population cohorts. Researchers can obtain the data from each cohort's ethics committee by applying for access. The analysis code is available from the corresponding author upon request for verification purposes.
Randomization	Our study design involves a population-based cohort approach, categorizing individuals into 'Regular PPI users' and 'Non-regular PPI users' based on their PPI exposure status. Random allocation is not applicable in this context due to the observational nature of population-based cohort studies. To control for confounding variables, we employed multivariable Cox regression models in the statistical analyses. For instance, we applied multivariable time-dependent Cox regression models for the three US cohorts, stratified by age and time period (in 2-year intervals). Additionally, we adjusted for ethnicity, BMI, smoking status, alcohol consumption, physical activity, overall diet quality, portions of fruit and vegetable intake, family history of specific diseases, clinical indication for PPI use, medications, prevalence of comorbidities, and female-specific indicators (for NHS and NHS II only).
Blinding	Our study is an observational cohort study, and blinding was not feasible during both data collection and analysis due to the inherent nature of the study design. The absence of blinding is a common characteristic in observational studies, where investigators observe and analyze data without intervening in participant allocation or treatment assignment.

Behavioural & social sciences study design

All studies must disclose on these points even when the disclosure is negative.

Study description	
Research sample	
Sampling strategy	
Data collection	
Timing	
Data exclusions	
Non-participation	
Randomization	

Ecological, evolutionary & environmental sciences study design

All studies must disclose on these points even when the disclosure is negative.

Study description	
Research sample	
Sampling strategy	
Data collection	
Timing and spatial scale	
Data exclusions	
Reproducibility	
Randomization	
Blinding	

Did the study involve field work? ☐ Yes ☒ No

Field work, collection and transport

Field conditions	
Location	
Access & import/export	
Disturbance	

Reporting for specific materials, systems and methods

We require information from authors about some types of materials, experimental systems and methods used in many studies. Here, indicate whether each material, system or method listed is relevant to your study. If you are not sure if a list item applies to your research, read the appropriate section before selecting a response.

Materials & experimental systems

n/a	Involved in the study
☒	☐ Antibodies
☒	☐ Eukaryotic cell lines
☒	☐ Palaeontology and archaeology
☒	☐ Animals and other organisms
☒	☐ Clinical data
☒	☐ Dual use research of concern
☒	☐ Plants

Methods

n/a	Involved in the study
☒	☐ ChIP-seq
☒	☐ Flow cytometry
☒	☐ MRI-based neuroimaging

Antibodies

Antibodies used	
Validation	

Eukaryotic cell lines

Policy information about cell lines and Sex and Gender in Research

Cell line source(s)	
Authentication	
Mycoplasma contamination	
Commonly misidentified lines (See ICLAC register)	

Palaeontology and Archaeology

Specimen provenance	
Specimen deposition	
Dating methods	

☐ Tick this box to confirm that the raw and calibrated dates are available in the paper or in Supplementary Information.

Ethics oversight	

Note that full information on the approval of the study protocol must also be provided in the manuscript.

Animals and other research organisms

Policy information about studies involving animals; ARRIVE guidelines recommended for reporting animal research, and Sex and Gender in Research

Laboratory animals	
Wild animals	
Reporting on sex	
Field-collected samples	
Ethics oversight	

Note that full information on the approval of the study protocol must also be provided in the manuscript.

Clinical data

Policy information about clinical studies

All manuscripts should comply with the ICMJE guidelines for publication of clinical research and a completed CONSORT checklist must be included with all submissions.

Clinical trial registration	
Study protocol	
Data collection	
Outcomes	

Dual use research of concern

Policy information about dual use research of concern

Hazards

Could the accidental, deliberate or reckless misuse of agents or technologies generated in the work, or the application of information presented in the manuscript, pose a threat to:

No	Yes	
☐	☐	Public health
☐	☐	National security
☐	☐	Crops and/or livestock
☐	☐	Ecosystems
☐	☐	Any other significant area

Experiments of concern

Does the work involve any of these experiments of concern:

No	Yes	
☐	☐	Demonstrate how to render a vaccine ineffective
☐	☐	Confer resistance to therapeutically useful antibiotics or antiviral agents
☐	☐	Enhance the virulence of a pathogen or render a nonpathogen virulent
☐	☐	Increase transmissibility of a pathogen
☐	☐	Alter the host range of a pathogen
☐	☐	Enable evasion of diagnostic/detection modalities
☐	☐	Enable the weaponization of a biological agent or toxin
☐	☐	Any other potentially harmful combination of experiments and agents

Plants

Seed stocks

Our study is a population-based cohort investigation, and it does not involve seed stocks or plant material.

Novel plant genotypes

Our study is an observational cohort study, and it does not involve novel plant genotypes. Therefore, methods related to transgenic approaches, gene editing, mutagenesis, or hybridization are not applicable to our research.

Authentication

Authentication procedures, mutation effect experiments, and assessments for potential secondary effects are not applicable to our population-based cohort study, as it does not involve seed stocks, novel genotypes, or genetic manipulations.

ChIP-seq

Data deposition

- [ ] Confirm that both raw and final processed data have been deposited in a public database such as GEO.
- [ ] Confirm that you have deposited or provided access to graph files (e.g. BED files) for the called peaks.

Data access links
May remain private before publication.

Files in database submission

Genome browser session
(e.g. UCSC)

Methodology

Replicates

Sequencing depth

Antibodies

Peak calling parameters

Data quality

Software

Flow Cytometry

Plots

Confirm that:

- [ ] The axis labels state the marker and fluorochrome used (e.g. CD4-FITC).
- [ ] The axis scales are clearly visible. Include numbers along axes only for bottom left plot of group (a 'group' is an analysis of identical markers).
- [ ] All plots are contour plots with outliers or pseudocolor plots.
- [ ] A numerical value for number of cells or percentage (with statistics) is provided.

Methodology

Sample preparation

Instrument

Software

Cell population abundance

Gating strategy

☐ Tick this box to confirm that a figure exemplifying the gating strategy is provided in the Supplementary Information.

Magnetic resonance imaging

Experimental design

Design type

Design specifications

Behavioral performance measures

Acquisition

Imaging type(s)

Field strength

Sequence & imaging parameters

Area of acquisition

Diffusion MRI ☐ Used ☐ Not used

Preprocessing

Preprocessing software

Normalization

Normalization template

Noise and artifact removal

Volume censoring

Statistical modeling & inference

Model type and settings

Effect(s) tested

Specify type of analysis: ☐ Whole brain ☐ ROI-based ☐ Both

Statistic type for inference

(See Eklund et al. 2016)

Correction

Models & analysis

n/a	Involved in the study
☐	☐ Functional and/or effective connectivity
☐	☐ Graph analysis
☐	☐ Multivariate modeling or predictive analysis

Functional and/or effective connectivity

Graph analysis

Multivariate modeling and predictive analysis
